# *Plasmodium vivax* and *Plasmodium falciparum* infection dynamics: re-infections, recrudescences and relapses

**DOI:** 10.1186/s12936-018-2318-1

**Published:** 2018-04-17

**Authors:** Michael T. White, Stephan Karl, Cristian Koepfli, Rhea J. Longley, Natalie E. Hofmann, Rahel Wampfler, Ingrid Felger, Tom Smith, Wang Nguitragool, Jetsumon Sattabongkot, Leanne Robinson, Azra Ghani, Ivo Mueller

**Affiliations:** 1 MRC Centre for Outbreak Analysis & Modelling, Department of Infectious Disease Epidemiology, Imperial College London, Norfolk Place, London, W2 1PG UK; 2 Division of Population Health & Immunity, Walter and Eliza Hall Institute, 1G Royal Parade, Melbourne, VIC 3052 Australia; 3 Department of Parasites and Insect Vectors, Institut Pasteur, 25-28 Rue du Dr Roux, 75015 Paris, France; 4 Department of Medical Biology, University of Melbourne, Parkville, VIC 3010 Australia; 5 Vector Borne Diseases Unit, Papua New Guinea Institute of Medical Research, Madang and Maprik, Papua New Guinea; 6 Swiss Tropical and Public Health Institute, Socinstrasse 57, 4051 Basel, Switzerland; 7 University of Basel, Petersplatz 1, 4003 Basel, Switzerland; 8 Department of Molecular Tropical Medicine and Genetics, Faculty of Tropical Medicine, Mahidol University, 999 Phuttamonthon 4 Road, Salaya, Bangkok, 73170 Thailand; 9 Mahidol Vivax Research Unit, Faculty of Tropical Medicine, Mahidol University, 999 Phuttamonthon 4 Road, Salaya, Bangkok, 73170 Thailand; 10 ISGlobal, Barcelona Institute for Global Health, Hospital Clinic – Universitat de Barcelona, Rossello, 08036 Barcelona, Spain

**Keywords:** *Plasmodium vivax*, *Plasmodium falciparum*, Relapse, Genotype, Statistical model

## Abstract

**Background:**

In malaria endemic populations, complex patterns of *Plasmodium vivax* and *Plasmodium falciparum* blood-stage infection dynamics may be observed. Genotyping samples from longitudinal cohort studies for *merozoite surface protein* (*msp*) variants increases the information available in the data, allowing multiple infecting parasite clones in a single individual to be identified. *msp* genotyped samples from two longitudinal cohorts in Papua New Guinea (PNG) and Thailand were analysed using a statistical model where the times of acquisition and clearance of each clone in every individual were estimated using a process of data augmentation.

**Results:**

For the populations analysed, the duration of blood-stage *P. falciparum* infection was estimated as 36 (95% Credible Interval (CrI): 29, 44) days in PNG, and 135 (95% CrI 94, 191) days in Thailand. Experiments on simulated data indicated that it was not possible to accurately estimate the duration of blood-stage *P. vivax* infections due to the lack of identifiability between a single blood-stage infection and multiple, sequential blood-stage infections caused by relapses. Despite this limitation, the method and data point towards short duration of blood-stage *P. vivax* infection with a lower bound of 24 days in PNG, and 29 days in Thailand. On an individual level, *P. vivax* recurrences cannot be definitively classified into re-infections, recrudescences or relapses, but a probabilistic relapse phenotype can be assigned to each *P. vivax* sample, allowing investigation of the association between epidemiological covariates and the incidence of relapses.

**Conclusion:**

The statistical model developed here provides a useful new tool for in-depth analysis of malaria data from longitudinal cohort studies, and future application to data sets with multi-locus genotyping will allow more detailed investigation of infection dynamics.

**Electronic supplementary material:**

The online version of this article (10.1186/s12936-018-2318-1) contains supplementary material, which is available to authorized users.

## Background

*Plasmodium falciparum* and *Plasmodium vivax* malaria parasites cause persistent blood-stage infections in humans lasting for weeks, months, and occasionally years [1]. The processes of parasite growth (as asexual merozoites invade red blood cells and periodically replicate every 48 h), density-dependent regulation, and the acquisition of adaptive immune responses cause complex patterns of blood-stage parasitaemia and infection dynamics [2–5]. In addition to blood-stage parasite replication, *P. vivax* has an alternative strategy for persisting in humans via the reservoir of hypnozoites in the liver [6]. Following inoculation of *P. vivax* sporozoites from an infectious mosquito, a proportion of sporozoites will develop into hypnozoites and remain arrested in the liver for weeks to years [7, 8], until they activate to cause new blood-stage infections. Blood-stage infections arising from the activation of *P. vivax* hypnozoites are referred to as relapses, and are a key distinguishing feature between the biology of *P. vivax* and *P. falciparum* [9].

A recurrent infection is defined as a newly detectable episode of blood-stage parasitaemia occurring after a previous infection [7]. A *P. falciparum* recurrence can be due to: (i) re-infection from a new mosquito bite; or (ii) recrudescence, where blood-stage parasites originating from a previous infection persist at sub-patent densities where the probability of detection is low, before increasing in density to become detectable. A *P. vivax* recurrence can be due to: (i) re-infection; (ii) recrudescence; or (iii) relapse. It is usually not possible to definitively distinguish between types of recurrences without detailed genotyping information. Re-infections can be excluded if an individual has moved to an area with no malaria transmission [10]. Relapses can be excluded if individuals are treated with an effective regimen of primaquine [11]—the only licensed drug capable of eliminating hypnozoites from the liver [12]. However, even with directly observed treatment, primaquine does not guarantee clearance of hypnozoites in human patients with a low CYP2D6 metabolizer phenotype [13]. Recrudescences can be excluded with high-sensitivity testing for blood-stage parasites, for example if prior to a positive blood sample an individual tested negative by qPCR, then it’s unlikely that the positive sample was due to a recrudescence.

Qualitative and quantitative descriptions of *P. falciparum* and *P. vivax* infection dynamics have been obtained through analysis of data from historic malaria-therapy studies used for the treatment of neuro-syphilis [14, 15]. In these highly controlled studies, exposure to infectious mosquito bites was strictly regulated and blood-stage parasite densities measured frequently, often daily, allowing for detailed investigation of temporal patterns in blood-stage parasite densities [2]. A number of limitations prevent the findings of these studies from being extrapolated to malaria-endemic regions, notably that subjects were malaria-naïve adults without pre-existing immunity, and the absence of super-infection with new strains.

In contemporary times, the dynamics of malaria infections may be studied in endemic regions using longitudinal cohort studies, where participants are followed over time and sampled frequently [16, 17].

Participants will have varying degrees of naturally-acquired immunity and may be concurrently exposed to both *P. vivax* and *P. falciparum* infections. Genotyping of *Plasmodium* parasites increases the information available from samples, allowing for super-infections to be identified, and multiplicity of infection to be estimated [18, 19]. These data can be analysed using statistical and mathematical models to estimate epidemiological parameters of interest. The dynamics of genotyped *P. falciparum* infections from longitudinal studies have previously been analysed using triplet or immigration-death models [20, 21], where the frequencies of different patterns of samples are analysed to provide estimates of the duration of blood-stage infection; the detectability of genotypes; and the force of infection of each genotype. A limitation of these methods is the assumption that duration of infection is exponentially distributed. In an analysis of *P. falciparum* samples from a longitudinal study in Ghana, Bretscher et al. [22] overcame this limitation by fitting a range of distributions to the duration of blood-stage infection, but at the expense of assuming re-infection with the same genotype is a rare event. Accounting for the role of *P. vivax* relapses in longitudinal studies remains a considerable challenge. Using data on time to relapse from malaria-therapy studies and keeping duration of blood-stage infection constant, Ross et al. [17] fitted a population-level model to data from a cohort of Papua New Guinean children, and estimated that 80% of *P. vivax* infections were attributable to relapses.

In this analysis, infection dynamics were estimated by estimating the times of acquisition and clearance of *P. falciparum* and *P. vivax* single-locus genotypes in each individual. Data from infections in all individuals were analysed simultaneously allowing estimation of the average duration of blood-stage infection, the probability of genotype detectability, and for *P. vivax*: the duration of liver-stage infection with hypnozoites and relapse frequency. Although a definitive classification of *P. vivax* infections into re-infection, recrudescence or relapse is not possible, it was possible to assign a probabilistic phenotype to each positive *P. vivax* sample, facilitating detailed investigation of the role of relapses in epidemiological studies.

## Methods

### Longitudinal cohort data

#### Maprik, Papua New Guinea

Between 2009 and 2010, 529 children aged 5–10 years were recruited in Maprik District, East Sepik Province, Papua New Guinea where both *P. falciparum* and *P. vivax* are hyperendemic [23]. Full details of the study are provided in Robinson et al. [11]. In brief, after screening for G6PD deficiency, 504 children were randomized to receive 20 days of directly observed treatment (DOT) over 4 weeks of either: (i) chloroquine (CQ) (DOTs 1–3), artemether-lumefantrine (AL) (DOTs 11–13), and primaquine (PQ) (DOTs 1–20; 0.5 mg/kg); or (ii) CQ (DOTs 1–3), AL (DOTs 11–13), and placebo (PL) (DOTs 1–20). The high dose of primaquine in arm (i) ensured effective elimination of hypnozoites from the liver. Children were followed longitudinally with active monitoring for infection by PCR and illness every 2 weeks for the first 12 weeks. From weeks 14–32 children were actively monitored for illness every 2 weeks and blood-samples for PCR were collected every 4 weeks. Active monitoring was supplemented via passive surveillance for symptomatic episodes through local health centres and aid posts. Clinical malaria infections (i.e. fever and parasitaemia confirmed by rapid diagnostic test) were treated with AL, thus hypnozoites were not eliminated during follow-up in both arms. An overview of the data is provided in Table 1.

**Table 1:** Overview of longitudinal cohort data from Papua New Guinea and Thailand

	Papua New Guinea (*n* = 504)		Thailand (*n* = 999)
	Placebo arm (*n* = 257)	Primaquine arm (*n* = 247)	
Gender (male)	49.8% (128)	48.6% (120)	46.2% (462)
Age (years)	7.5 (4.9, 10.4)	7.6 (4.8, 10.4)	23 (1, 82)
Bednet usage	93.4% (240)	93.1% (230)	88.8% (888)
Duration of follow-up (days)	224 (56, 231)	224 (42, 229)	369 (360, 378)
Proportion of data missing	12.3%	10.1%	8.3%
Any *P. vivax* infection by qPCR	69.6% (179)	27.9% (69)	11.9% (119)
Any *P. vivax* infection by LM	54.5% (140)	21.9% (54)	–
Number of *P. vivax* genotypes	2.2 (0, 12)	0.7 (0, 7)	0.3 (0, 11)
Any *P. falciparum* infection by qPCR	37.0% (95)	34.0% (84)	2.4% (24)
Any *P. falciparum* infection by LM	23.7% (61)	27.9% (69)	–
Number of *P. falciparum* genotypes	0.6 (0, 9)	0.5 (0, 6)	0.03 (0, 6)
Any fever	53.7% (138)	62.3% (154)	24.7% (247)
Any fever with qPCR^+^ *P. vivax*	10.1% (26)	4.0% (10)	2.1% (21)
Any fever with qPCR^+^ *P. falciparum*	9.7% (25)	16.6% (41)	0.3% (3)

The data from PNG is for the period after the initial drug regimen. Missing data is defined as the proportion of samples scheduled in the study protocols missed. Data on age, numbers of genotypes detected during follow-up, and duration of follow-up are presented as mean and ranges. Light microscopy (LM) data was not available for the Thai samples

#### Kanchanaburi and Ratchaburi, Thailand

Between 2013 and 2014, 999 participants aged 1–82 years were recruited in Kanchanaburi and Ratchaburi provinces of western Thailand, where moderate levels of *P. vivax* transmission and low levels of *P. falciparum* are reported. This cohort had substantially lower levels of *P. vivax* and *P. falciparum* infection than the Papua New Guinean cohort. Full details of the study are provided in Longley et al. [24]. In this observational cohort study, participants were not treated upon recruitment and were followed longitudinally for 12 months, with blood samples collected every month (14 active case detection visits in total). Active monitoring was supplemented by samples recruited through passive case detection where participants with temperature ≥ 37.5 °C or with a history of fever were checked for malaria using a rapid diagnostic test (RDT). RDT positive individuals were referred to local health clinics where Thai malaria treatment guidelines applied: chloroquine and primaquine for *P. vivax* and artemisinin-based combination therapy (ACT) for *P. falciparum*. All samples were assessed by qPCR for the presence of *P. falciparum* or *P. vivax* parasites. qPCR positive samples were then genotyped. An overview of the data is presented in Table 1.

#### Simulated data

Simulated data sets on the dynamics of *P. falciparum* and *P. vivax* genotypes in longitudinal studies were generated across a range of parameter values for the duration of blood-stage infection, genotype detectability and time to relapse. Details are provided in Additional file 3. The simulated data sets were assumed to have demography matching the Papua New Guinean cohort.

#### Laboratory methods and genotyping

Laboratory studies were undertaken in Basel for the Papua New Guinean samples and in Bangkok for the Thai samples, with external quality control undertaken in The Walter and Eliza Hall Institute in Melbourne (C Koepfli). Finger-prick blood samples collected during follow-up were separated into plasma and red cell pellets and stored at − 80 and − 20 °C, respectively. DNA was extracted using the FavorPrep 96-Well Genomic DNA Extraction Kit (Favorgen, Taiwan) from the red cell pellet fraction of all samples. *Plasmodium* spp. infections were detected using a generic qPCR to detect all four species [25], after which species-specific (*P. falciparum*, *P. vivax*, *Plasmodium malariae*, and *Plasmodium ovale*) qPCRs were performed on *Plasmodium*-positive samples [26]. All PCRs were conducted on 4 µL DNA, corresponding to 4 µL blood. To determine the number of genetically distinct blood-stage clones in each sample, *P. vivax* and *P. falciparum* positive samples were genotyped. Size polymorphic molecular markers were amplified by a nested PCR, followed by capillary electrophoresis for sizing. For *P. vivax* the marker *msp1*F3 [19] was typed, and for *P. falciparum* the marker *msp2* [27]. These genotyping markers have expected heterozygosity in excess of 90%. The limit of detection of the molecular methods was 1–3 copy numbers/µL [28]. Some samples were qPCR positive for *P. falciparum* or *P. vivax*, but did not test positive for any genotype. These samples were assumed to be negative for all considered genotypes. For the present model the 14 most common alleles were included. In Thailand they accounted for 88% of all *P. falciparum* and 76% of all *P. vivax* clones, and in PNG for 88% of all *P. falciparum* and 86% of all *P. vivax* clones.

#### Data overview

**Fig. 1:**
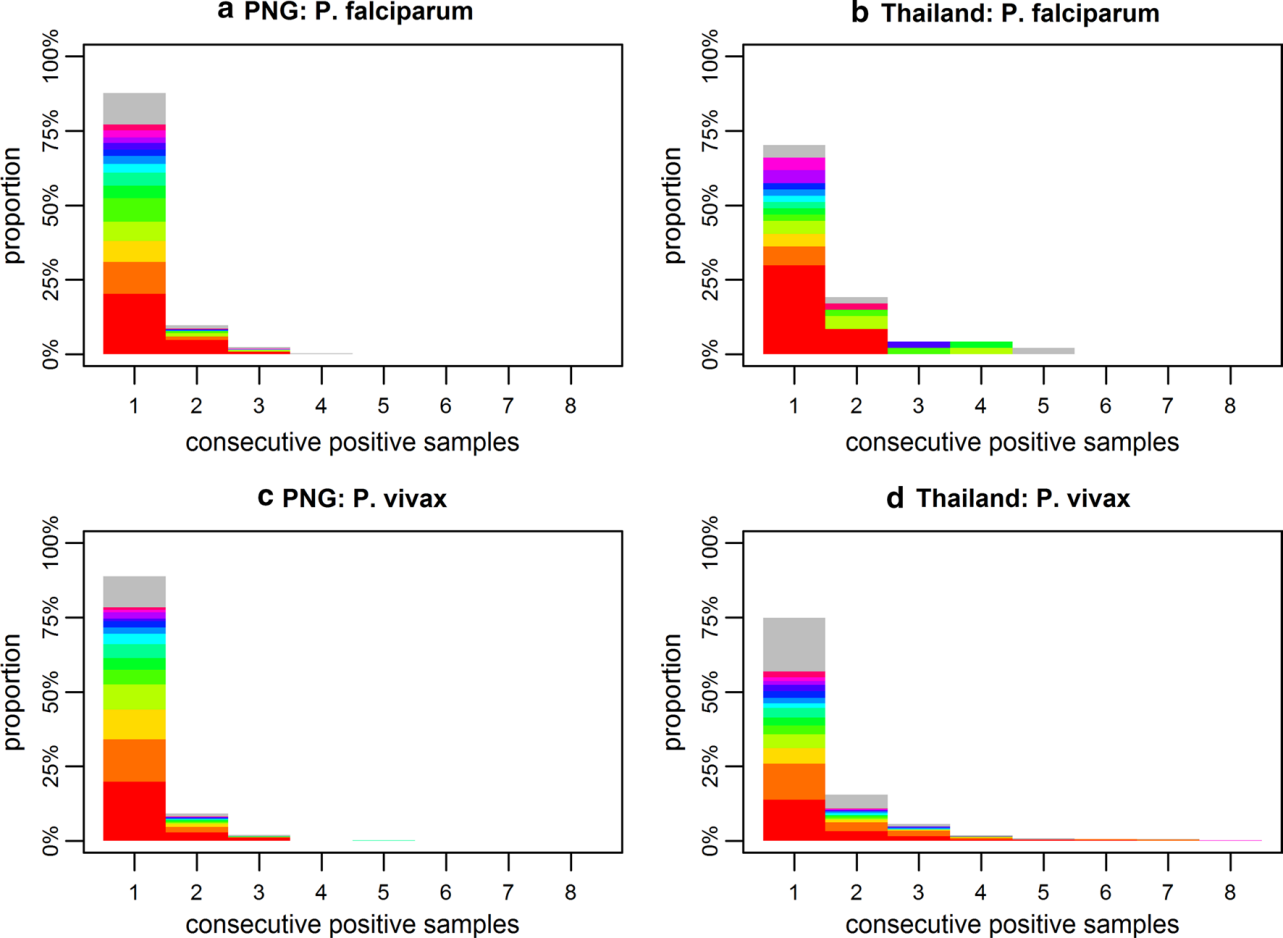
Distribution of number of consecutive positive samples for individuals infected with **a** *P. falciparum* in PNG; **b** *P. falciparum* in Thailand; **c** *P. vivax* in PNG; and **d** *P. vivax* in Thailand. The colours denote the frequency of the 14 most common genotypes. The grey region represents the frequency of the rest of the genotypes. The mean time between samples was 18 days in PNG, and 29 days in Thailand

For the 504 individuals in the PNG cohort, and the 999 individuals in the Thai cohort, a wide range patterns for the presence or absence of the *P. falciparum* and *P. vivax* genotypes were observed. Figure 1 shows a summary of these patterns in terms of the distribution of number of consecutive positive samples. The data patterns are further summarized in Additional file 1.

#### Statistical inference

The data and model have a three level structure. (i) On the individual-level, data from each genotype in each participant provides information on the times of acquisition and clearance of infections. (ii) The genotype-level combines data from all participants to provide information on the genotype-specific force of infection. (iii) The population-level combines data from all participants and all genotypes to provide information on duration of blood-stage and liver-stage infection, genotype detectability, and relapse frequency. A mixed-effects framework allows for a synthesis of information across all levels. For example, if samples are missing from one individual the infection dynamics during the period of missed sampling will depend on the population-level parameters. Statistical inference was implemented in a Bayesian framework with parameters updated according to a Metropolis–Hastings MCMC algorithm implemented in C++. Prior information on population-level parameters was assumed based on previously published studies (Table 2). Full details are provided in Additional file 2.

**Table 2:** Population-level parameter estimates for the *P. falciparum* and *P. vivax* infection dynamics models fitted to data from PNG and Thailand

Description	Parameter	Prior	*P. falciparum*		*P. vivax*	
			PNG	Thailand	PNG	Thailand
Blood-stage duration (days) [4]	*d*_*BS*_	60 (16, 132)	36 (29, 44)	135 (94, 191)	24 (21, 28)	29 (27, 32)
Shape parameter	*κ*	2.0 (0.5, 4.4)	0.50 (0.45, 0.57)	1.15 (0.79, 1.78)	0.54 (0.49, 0.62)	0.40 (0.38,0.42)
*Pf* detectability [43]	*q*	70% (64%, 76%)	66% (60%, 71%)	67% (61%, 73%)	–	–
*Pv* detectability [43]	*q*	48% (42%, 54%)	–	–	44% (40%, 49%)	38% (35%, 41%)
Time to relapse (days) [7]	1/*f*	50 (19, 96)	–	–	41 (35, 49)	55 (40, 80)
Liver-stage duration (days) [30]	1/*γ*_*L*_	250 (195, 312)	–	–	383 (313, 467)	226 (181, 278)

Notably, the estimates for blood-stage infection are for the duration when parasites are detectable by molecular genotyping. Prior parameter estimates are derived from the cited studies. Parameters are presented as estimated posterior medians with 95% credible intervals. The estimates for the *P. vivax* parameters should be interpreted in light of the results of the experiments on simulated data which indicated that it was not always possible to consistently and accurately estimate the duration of blood-stage infection and the time to relapse

#### Model overview

**Fig. 2:**
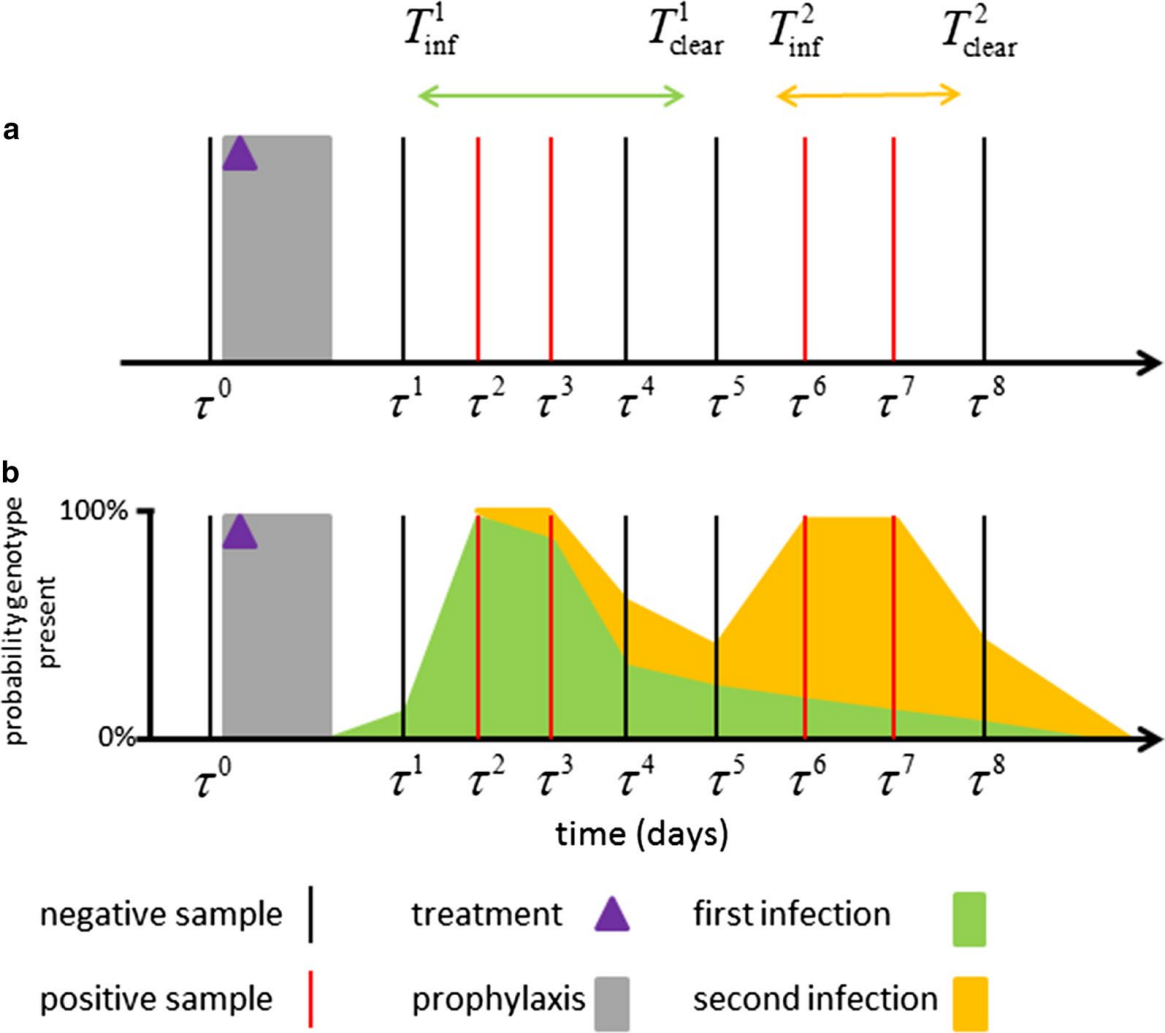
Schematic of infection dynamics of a single genotype from an individual in a longitudinal cohort study. **a** Samples are collected at times *τ*^*j*^, and are either negative (*x*^*j*^ = 0) or positive (*x*^*j*^ = 1). In this example the samples after the period of prophylaxis can be encoded as 01100110. Examples of possible times for the acquisition *T*_inf_ and clearance *T*_clear_ of two infections are shown. **b** The exact values of *T*_inf_ and *T*_clear_ are unknown and hence are treated as parameters whose distributions are estimated via data augmentation. The green region denotes the probability that the first infection is present at that time. The orange region denotes the probability that the second infection of the same genotype is present at that time

Genotyping of malaria infections in longitudinal cohorts allows for multiple infections in the same individual originating from different mosquito bites to be distinguished from one another. The infection dynamics of a *P. falciparum* or *P. vivax* single-locus genotype within an individual can be represented schematically as shown in Fig. 2. Sample *j* measured at time point $\tau^{j}$ can be either positive (*x*^*j*^= 1) or negative (*x*^*j*^= 0) for a given genotype. The sequence of positive and negative samples in Fig. 2 could be due to two separate infections of the same genotype, with the first infection acquired at $T_{\inf }^{1} \in (\tau^{1} ,\tau^{2} )$ and clearing at $T_{\text{clear}}^{1} \in (\tau^{3} ,\tau^{5} )$, and the second infection acquired at $T_{\inf }^{2} \in (\tau^{3} ,\tau^{5} )$ and clearing at $T_{\text{clear}}^{2} \in (\tau^{7} ,\tau^{8} )$. Alternatively, with a probability depending on the genotype detectability *q*, the four positive samples could be from a single infection that was not detected in the samples at $\tau^{4}$ and $\tau^{5}$. Importantly, the acquisition and clearance times are unknowable (the examples depicted in Fig. 2a are just one possible combination). Instead their distribution is estimated by treating them as parameters in a process of data augmentation.

Given *P. falciparum* genotype data in an individual, the likelihood that the samples are described by acquisition times $T_{\inf }^{k}$ and clearance times $T_{\text{clear}}^{k}$ can be calculated, where each separate infection is indexed by *k*. This requires us to assume values for the detectability *q*, the force of infection of that genotype λ, and a distribution for the duration of blood-stage infection of a single genotype (e.g., a Weibull distribution with mean *d*_*BS*_ and shape parameter *κ*_*WB*_) [22]. Notably, the duration of blood-stage infection $d_{BS}^{k} = T_{\text{clear}}^{k} - T_{\inf }^{k}$ will be limited to the period when it is detectable via molecular genotyping. The augmented parameters $T_{\inf }^{k}$ and $T_{\text{clear}}^{k}$ can then be updated using a Markov Chain Monte Carlo (MCMC) routine. Upon convergence, the MCMC chains will provide distributions for $T_{\inf }^{k}$ and $T_{\text{clear}}^{k}$.

When this method is applied to data from individual participants, knowledge of population-level parameters (*q*, λ, *d*_*BS*_, *κ*_*WB*_) is required. However, these population-level parameters are not necessarily known for a given transmission setting, so they are estimated simultaneously with the individual-level augmented parameters. Statistical inference is implemented in a multi-level framework, so that updating population-level parameters affects the likelihood for all individuals, and the augmented infection time parameters are updated for one individual at a time. When patterns from multiple individuals and multiple genotypes are combined, information on the infection dynamics on a population level can be obtained. For example, if there are many 00100 patterns this would indicate short durations of blood-stage infection (or low detectability). If there are many 01110 patterns this would indicate longer durations of blood-stage infection and higher detectability.

The *P. falciparum* model allows for probabilistic differentiation between re-infection (due to new mosquito bites) and recrudescence (due to a blood-stage sample that has previously gone undetected). In order to account for *P. vivax* relapses, population-level parameters describing the epidemiology of relapses need to be defined [29, 30]. Define *f* to be the relapse frequency. Relapses are assumed to occur at a constant rate so that the average time between primary attack and first relapse will be 1/*f*, the average time between first and second relapses will be 1/*f*, and so on as long as hypnozoites are present in the liver. Notably, a relapse can occur when blood-stage parasites from the primary infection are still circulating, but it will be impossible to detect if the parasites have the same genotype. Define *γ*_*L*_ to be the rate of clearance of liver-stage infection, such that the average time the liver spends infected with hypnozoites after an infectious bite is 1/*γ*_*L*_. Given the population-level parameters describing the epidemiology of relapses (*f* and *γ*_*L*_), it can be calculated that the likelihood that *P. vivax* genotype data from an individual is described by infection times $T_{\inf }^{k}$ and $T_{\text{clear}}^{k}$. Notably, each new infection can be due to either a re-infection or a relapse, but not a recrudescence which is considered to be persistence of a previous infection. The probability that a new infection is a relapse can be calculated and will depend on the relapse frequency *f* and the genotype-specific force of infection *λ*. Full details of the statistical methodology are provided in Additional file 2.

## Results

### Assessment of model performance with simulated datasets

**Fig. 3:**
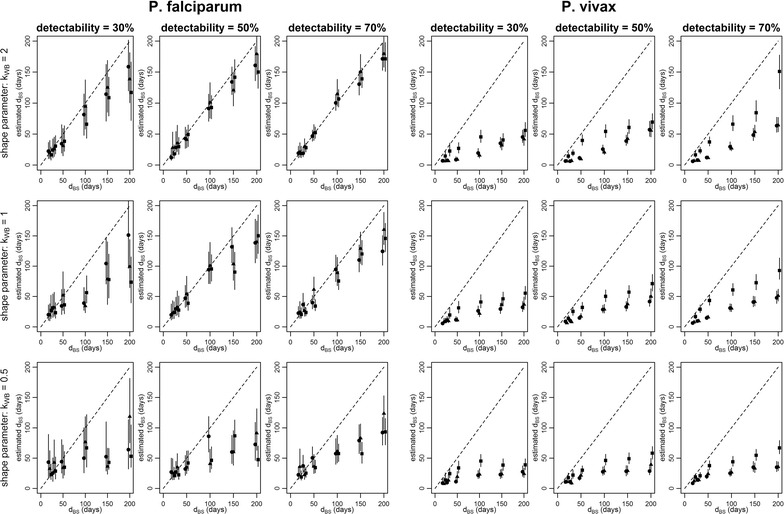
Results of model validation on simulated data. The x-axis shows the value for the duration of blood-stage infection (*d*_*BS*_) used to simulate the data, and the y-axis shows the estimated median and 95% credible intervals. Circles denote data simulated assuming no heterogeneity and seasonality of exposure to infectious mosquito bites. Triangles denote data simulated assuming heterogeneity in exposure. Squares denote data simulated assuming seasonality in exposure. Each panel represents a different combination of detectability (*q*) and shape parameter (*κ*_*WB*_) for either *P. falciparum* or *P. vivax*

The statistical methods were applied to simulated data sets with known values of the population-level parameters describing the infection dynamics of *P. falciparum* (*d*_*BS*_, *κ*_*WB*_, *q*, λ) and *P. vivax* (*d*_*BS*_, *κ*_*WB*_, *q*, *f*, *γ*_*L*_, λ), to investigate whether it was possible to accurately estimate these parameters (i.e. whether the method could reproduce these known parameter values). The full results are described in detail in Additional file 3. Data sets were simulated under assumptions of either: constant exposure to infectious mosquito bites; heterogeneity in exposure; or seasonality in exposure. Figure 3 shows the method’s performance for estimating the duration of blood-stage infection in simulated data. For *P. falciparum d*_*BS*_ was accurately estimated across large regions of parameter space. However, there was a notable tendency to underestimate *d*_*BS*_ when genotype detectability was low. Low genotype detectability leads to positive samples being missed, causing long sequences of consecutive samples to be split into several shorter sequences. *d*_*BS*_ was also underestimated for small values of *κ*_*WB*_—the shape parameter of the Weibull distribution. Smaller values of *κ*_*WB*_ correspond to greater variation in the duration of blood-stage infection, leading to many short infections and fewer long infections.

For the simulated *P. vivax* data, *d*_*BS*_ was consistently underestimated across all regions of parameter space. This leads to the conclusion that the method cannot reliably estimate the duration of blood-stage *P. vivax* infection because of a lack of identifiability between a blood-stage infection of long duration following a mosquito bite or a blood-stage infection of short duration rapidly followed by relapses. However, the estimates of *d*_*BS*_ can be viewed as a lower bound for the duration of blood-stage *P. vivax* infection. The underestimation of *d*_*BS*_ was accompanied by underestimation of the time to next relapse (1/*f*)—see Additional file 3. This issue of lack of identifiability of the duration of blood-stage *P. vivax* infection arises from the inability of the method to distinguish between a blood-stage infection with a single-locus genotype of long duration, and a blood-stage infection of short duration rapidly followed by relapses of the same genotype. The estimates of the duration of blood-stage *P. vivax* infections and the time to next relapse presented here should therefore be treated with caution.

### Single genotype infection dynamics

**Fig. 4:**
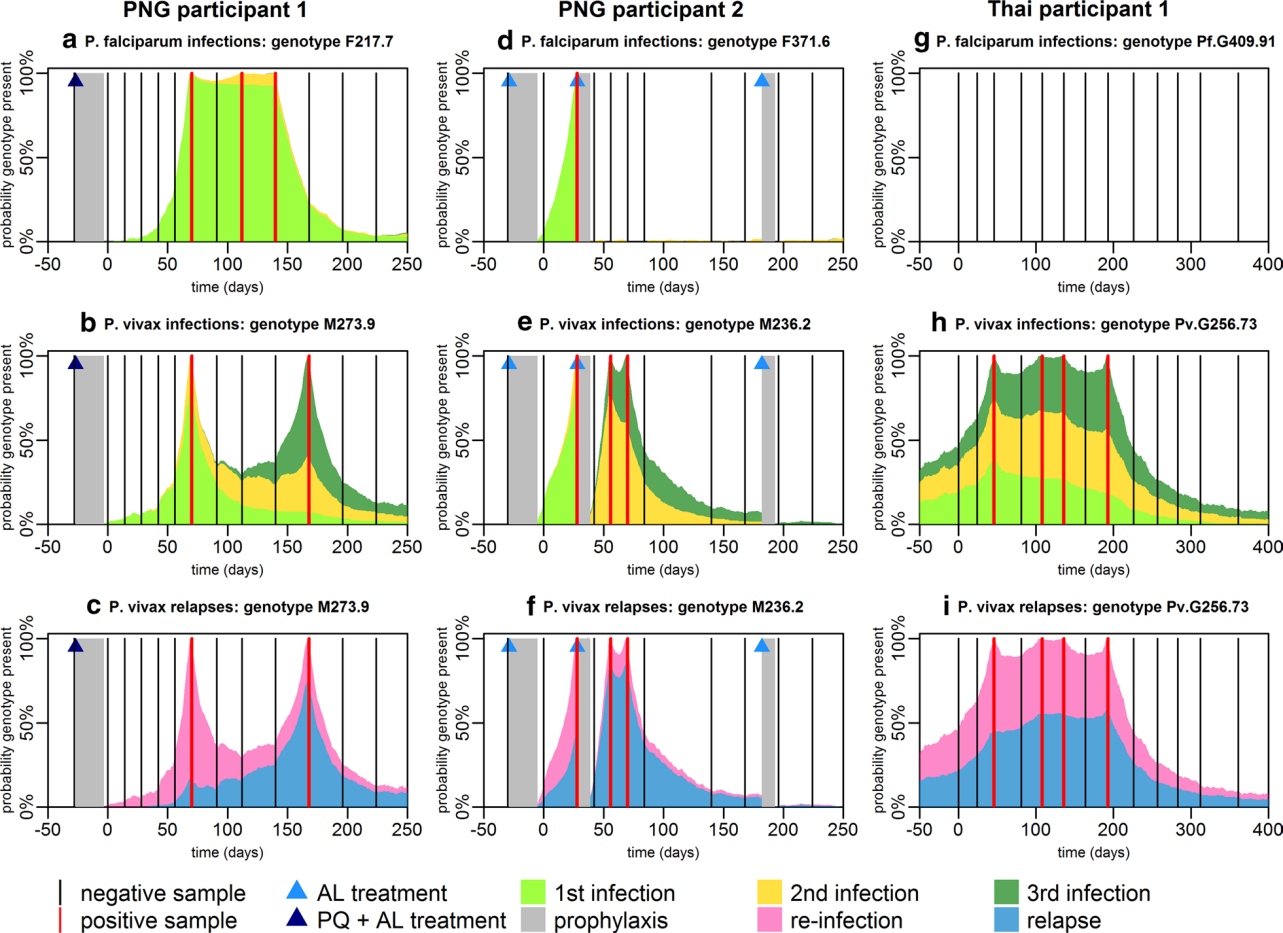
Probability of the presence of *P. falciparum* and *P. vivax* genotypes. **a**–**c** Infection dynamics in PNG participant 1. **d**–**f** Infection dynamics in PNG participant 2. **g**–**i** Infection dynamics in Thai participant 1. For each genotype within each individual, the colours denote whether that genotype is predicted to be present at a given time arose from that individual’s first, second or third infection. Each *P. falciparum* infection comes from a separate mosquito bite. Each *P. vivax* infection comes from either a new mosquito bite or a relapse. The bottom row depicts the probability that a new infection with a *P. vivax* genotype is due to re-infection from mosquito or relapse. The grey region denotes the period of prophylaxis when participants were treated with AL and chloroquine, and either primaquine or a placebo. During the period of follow-up AL treatment was administered by the investigators to some of the Papua New Guinean participants

For each of the 14 most common *P. falciparum* and *P. vivax* genotypes in all individuals in the PNG and Thai cohorts, the blood-stage infection dynamics were estimated. Figure 4 shows the infection dynamics of single *P. falciparum* and *P. vivax* genotypes in three individuals. For each positive sample, it is possible to read off from the figure the estimated probability that the detected parasites are from the first, second or third infection. In PNG participant 1, the three positive *P. falciparum* samples (Fig. 4a) are due to a single infection with 94% probability (i.e. a single infection spans the full duration of these samples), or two separate infections with 6% probability. The two positive *P. vivax* samples (Fig. 4b) are from the same infection with 8% probability or from separate infections with 92% probability. Each infection in Fig. 4b is due to either a re-infection or a relapse. Figure 4c shows these infections’ breakdown according to the probability of being a relapse or not. The first positive sample (70 days after the end of the primaquine course) is estimated to be a relapse with 17% probability. However the second positive sample at 168 days is a relapse with 74% probability. Figure 4d–f shows the results from an individual in the placebo arm of the PNG cohort. This individual received two AL treatments clearing blood-stage infections. In particular, after first treatment, the *P. vivax* genotype will reappear very rapidly, with a high probability of it being a relapse (Fig. 4f). Figure 4g–i shows the dynamics of a *P. falciparum* and *P. vivax* genotype in an individual in the Thai cohort. *P. falciparum* infection (of any genotype) was rare, and most participants in the Thai cohort remained free from infection. Figure 4 shows the infection dynamics for 2/504 Papua New Guinean participants and 1/999 Thai participants. The infection dynamics for an additional six participants are shown in Additional file 4.

### Multiple genotype infection dynamics

**Fig. 5:**
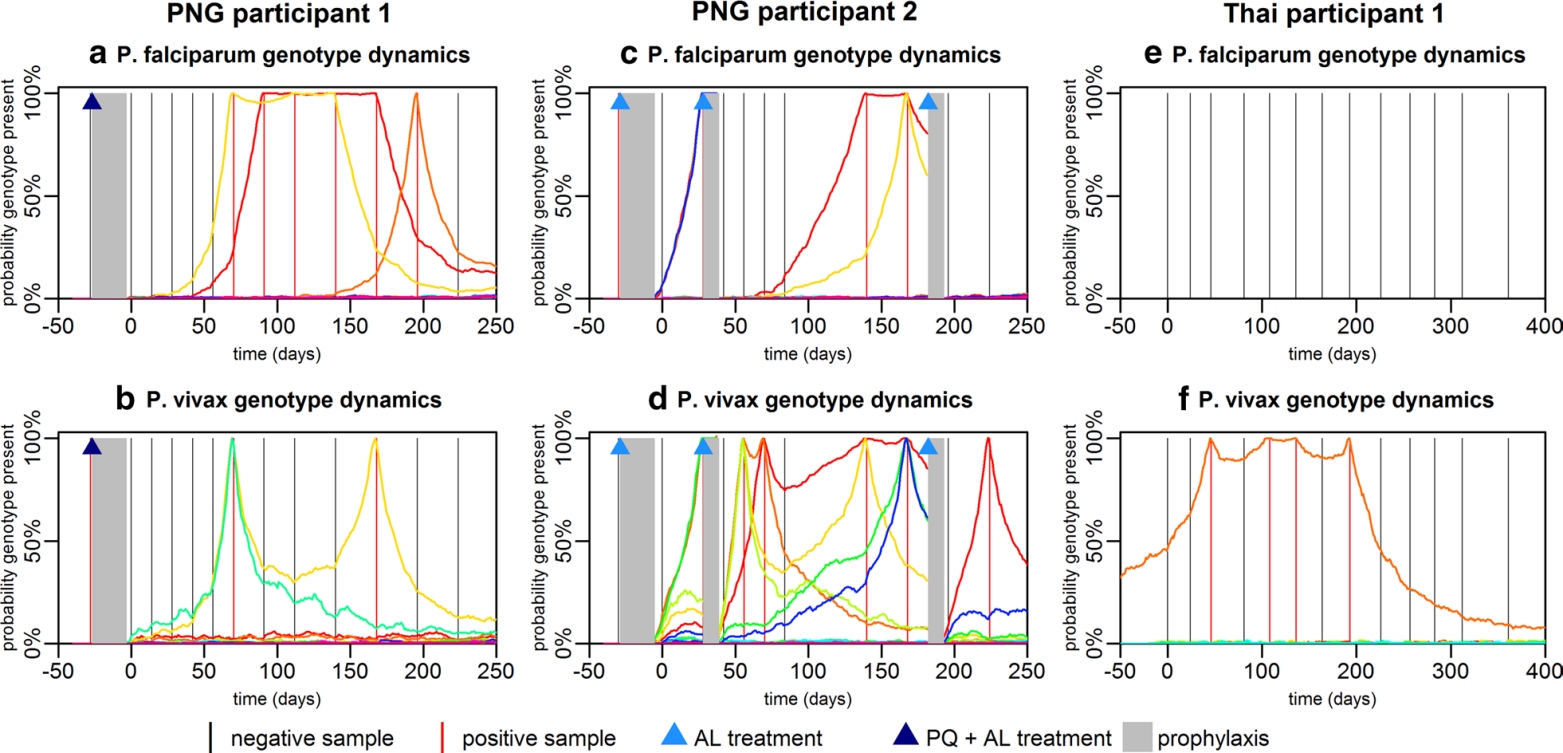
Infection dynamics of multiple *P. falciparum* and *P. vivax* genotypes. **a**, **b** Infection dynamics in PNG participant 1. **c**, **d** Infection dynamics in PNG participant 2. **e**, **f** Infection dynamics in Thai participant 1. For both *P. falciparum* and *P.* vivax each genotype is represented by a different colour

For each individual the infection dynamics of each genotype can be combined to describe the diversity of infection. Figure 5 shows the infection dynamics for the 14 most common genotypes for the same three individuals depicted in Fig. 4. What may appear to be single *P. falciparum* or *P. vivax* blood-stage infections when samples are tested with light microscopy or PCR, is often a complex series of infections. In particular, for PNG participant 2 in the placebo arm where liver-stage hypnozoites were not removed, a very high diversity of *P. vivax* infection was observed, even after blood-stage parasites are removed with AL treatment. In Thailand, where transmission intensity was much lower, fewer genotypes within each blood-stage infection were observed. The infection dynamics of multiple *P. falciparum* and *P. vivax* genotypes for an additional six individuals are shown in Additional file 4.

### Population-level infection dynamics

**Fig. 6:**
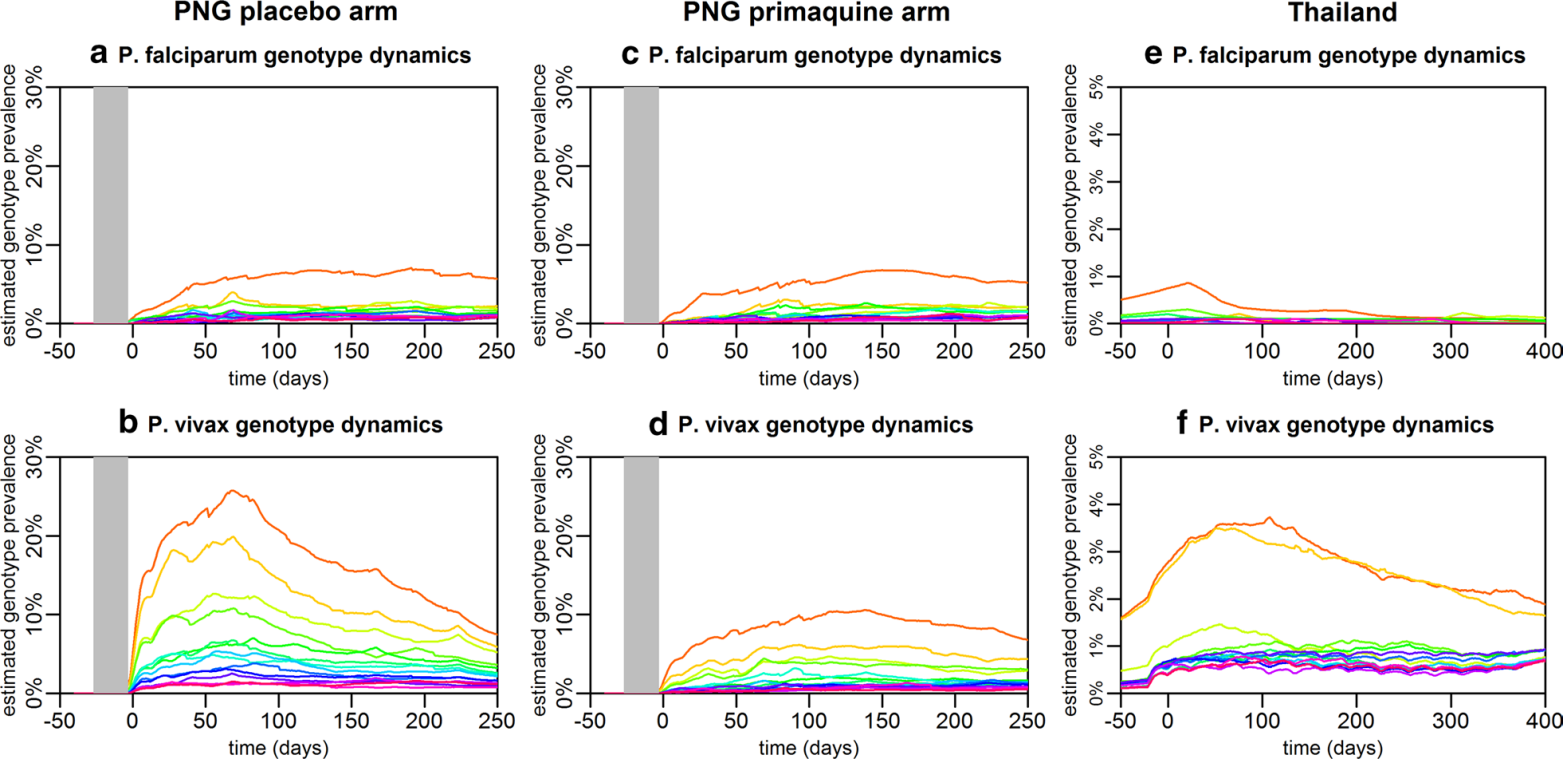
Predicted prevalence of the 14 most common *P. falciparum* and *P. vivax* genotypes in **a**, **b** the PNG placebo arm; **c**, **d** the PNG primaquine arm; and **e**, **f** Thailand. For both *P. falciparum* and *P. vivax* each genotype is represented by a different colour. The period of prophylactic protection in PNG is shown in grey

The infection dynamics for each genotype in each individual can be aggregated to provide an overview of the infection dynamics on the population level. Figure 6 shows estimated genotype prevalence in both cohorts. The estimated prevalence is systematically greater than the observed prevalence (Additional file 4: Fig. S4.5) due to imperfect genotype detection. The temporal variation in *P. vivax* genotype prevalence in Thailand (Fig. 6f) is most likely due to seasonal variation in malaria transmission [24]—a pattern also seen for *P. falciparum* (Fig. 6e), albeit more weakly due to the small number of positive samples. In contrast, there is no clear seasonal pattern in *P. falciparum* genotype prevalence in PNG (Fig. 6a, c), despite previous observations of seasonal variation in exposure to infectious mosquito bites in the study region [23]. In the placebo arm of the PNG study, a notable peak in *P. vivax* genotype prevalence around day 70 was observed (Fig. 6b). As a similar peak is not observed in *P. vivax* genotype prevalence in the primaquine arm (Fig. 6a), or of *P. falciparum* genotype prevalence in either arm (Fig. 6c, d), it is likely that the delayed peak in *P. vivax* following blood-stage treatment is due to a factor other than seasonality. This peak most likely arises from relapsing hypnozoites originating from mosquito bites before or during the period of prophylactic protection. However, if exposure to *P. vivax* infectious mosquitoes is assumed to be constant, then the incidence of relapses in a population is also expected to be constant, and hence a reduction in *P. vivax* geneotype prevalence after the peak at day 70 is not expected. The reduction in *P. vivax* prevalence following the peak at day 70 must, therefore, be due to some factor not explicitly accounted for in this model. Some plausible hypotheses for this pattern are: (i) the initial treatment regimen before day 0 triggers relapses [31]; (ii) blood-stage parasites suppress the development of liver-stage parasites [32], and clearance of blood-stage parasitaemia increases the development rate of liver-stage parasites; (iii) short-lived genotype-specific immune responses wane during the period of prophylactic protection leading to a rebound in infection rates [33]; or (iv) blood-stage parasites originating from relapses are suppressed but not cleared by the prophylactic effects of AL and CQ, leading to an accumulation of low density infections that rebound once drug concentrations wane.

### Population-level parameter estimates

Table 2 presents posterior estimates of the population-level parameters. In PNG children living in an area with hyperendemic *P. falciparum* transmission, the duration of blood-stage infection detectable by molecular genotyping was 36 (95% credible interval (CrI): 29, 44) days. In Thailand where *P. falciparum* infection was rare, the duration of blood-stage infection was 135 (95% CrI 94, 191) days. The shape parameter for the distribution of the duration of *P. falciparum* infection in PNG was 0.50 (95% CrI 0.45, 0.57) < 1, suggesting over-dispersion in infection, i.e. many short-lived infections with a small number of infections of long duration. In Thailand, the estimated shape parameter for *P. falciparum* was 1.15 (95% CrI 0.79, 1.78). Notably a shape parameter of 1 corresponds to an Exponential distribution for the duration of infection. The duration of blood-stage *P. vivax* infections was shorter than the duration of blood-stage *P. falciparum* infection in both settings: 24 (95% CrI 21, 28) days in PNG, and 29 (95% CrI 27, 32) days in Thailand. However, these estimates of the duration of blood-stage *P. vivax* infection should be interpreted in light of the results from the model validation on simulated data, where it was found that this parameter was not always identifiable. In PNG, which had intense levels of *P. vivax* transmission the average duration of liver-stage infection was 383 (95% CrI 313, 467) days. In Thailand, where *P. vivax* transmission was low, the estimate of the duration of liver-stage infection did not differ significantly from the prior distribution for this parameter (Table 2).

### Proportion of *P. vivax* infections attributable to relapses

There are at least two perspectives on how the proportion of recurrent *P. vivax* infections attributable to relapses can be estimated. Firstly, the probability that an individual’s next infection is a relapse can be considered. Secondly, the proportion of total infections that are due to relapses can be considered (possibly including repeated acquisition of the same genotype due to multiple relapses). If no liver-stage hypnozoites are present in an individual, the next infection must be a primary infection from a mosquito bite. An infectious mosquito bite will be followed by a period of blood-stage parasitaemia. During this period, relapses of the same genotype may occur (i.e. hypnozoites activate), however this event will be impossible to detect as it is not possible distinguish between clonal blood-stage parasites from primary infection or relapses using currently available methods. Furthermore, as the activation of hypnozoites is likely to be highest immediately after primary infection [6] when infected individuals are also likely to have the greatest number of hypnozoites, the proportion of relapses hidden by blood-stage parasitaemia from the primary infection may be quite substantial. As many relapses are likely to be undetectable, the estimated proportion of total infections that are due to relapses will be higher than the probability that a recurrent blood-stage infection is initiated by a relapse.

**Fig. 7:**
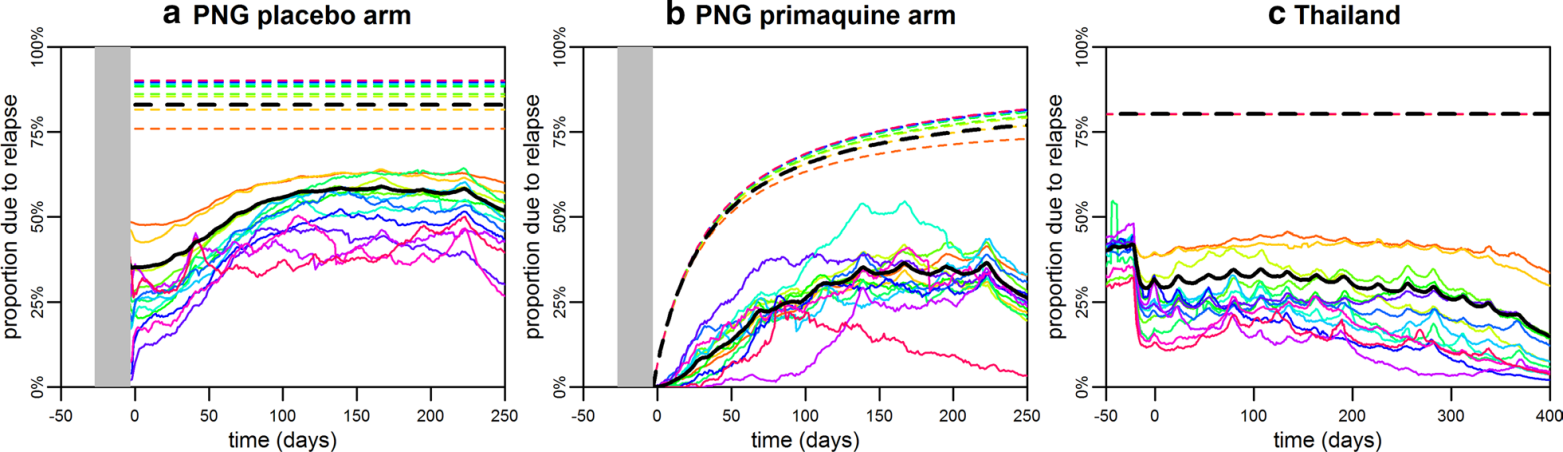
Population-level proportion of *P. vivax* relapses in **a** the PNG placebo arm; **b** the PNG primaquine arm; and **c** Thailand. Solid lines show the proportion of new *P. vivax* infections due to relapses, and dashed lines show the proportion of total *P. vivax* infections due to relapses. The coloured lines depict the result for each genotype and the black lines show the average across all genotypes. The difference between the solid and dashed lines is due to relapses that are undetectable because they occur when blood-stage parasites of the same genotype are already circulating. If an individual has not been recently exposed, then their next *P. vivax* infection is more likely to be from a mosquito bite. For example, in low transmission Thailand, if an individual has had no detectable blood-stage infection for > 9 months, the probability that a new infection is due to a relapse is low, as liver-stage infection of this duration without relapsing is unlikely. However, once a new infection does occur, it is likely to be followed by multiple relapses hence the high proportion of total infections due to relapses

For a new *P. vivax* infection of a given genotype in an individual, the probability that it was initiated by a relapse can be estimated (Fig. 4). Figure 7 shows how these probabilities can be aggregated across all individuals to provide estimates of the proportion of infections due to relapses of each genotype on the population-level (solid lines). When the ratio of relapses to total infections is estimated (dashed lines in Fig. 7), relapses constituted 76–90% of total infections in the placebo arm of the PNG cohort, and 79% in the Thai cohort. In the primaquine arm of the PNG cohort, the proportion of relapses is predicted to be zero immediately after clearance of liver-stage hypnozoites, increasing over the course of the study to the same levels as observed in the placebo arm. Notably, for more common genotypes, a lower proportion of infections is expected to be attributable to relapses, because re-infection with the same genotype is more likely.

## Discussion

The statistical model presented here allows infection dynamics to be investigated on both an individual and a population-level. Data from two populations with different study designs were analysed: (i) a treatment-reinfection study in Papua New Guinean children, with and without clearance of liver-stage hypnozoites by primaquine; and (ii) a longitudinal observational cohort study in Thai participants of all ages. The average duration of *P. vivax* blood-stage infection in PNG was estimated as 24 (95% CrI 21, 28) days, slightly shorter than in Thailand (29 (95% CrI 27, 32) days), which had lower levels of *P. vivax* transmission. In PNG, which had moderate levels of *P. falciparum* transmission, the average duration of *P. falciparum* blood-stage infection was 36 (95% CrI 29, 44) days, substantially shorter than has been reported in some African populations in higher transmission settings, where it has been estimated to be 70–179 days [34]. In Thailand where infection with *P. falciparum* was rare, the average duration of blood-stage infection was 135 (95% CrI 94, 191) days. The variation in the estimated duration of blood-stage infection between different populations may be due to differences in levels of naturally-acquired immunity, population genetic differences in host response to infection, variation in strains selected for differing local environments, or the choice of analysis method.

When the performance of the model was assessed against simulated *P. falciparum* data, it was able to reliably estimate the population-level parameters (duration of blood-stage infection and detectability) across a wide range of parameter space. However, assessment of the statistical methods on simulated *P. vivax* data sets reveals a substantial limitation, that the duration of blood-stage *P. vivax* infection and the time to next relapse cannot be reliably estimated. This arises from a lack of identifiability between long blood-stage infections of a single-locus genotype and a short blood-stage infection rapidly followed by relapses of the same genotype. When a cohort was simulated assuming a long duration of blood-stage *P. vivax* infection, the duration of blood-stage infection was substantially underestimated. For example, for a blood-stage infection lasting 90 days, the method assigns a higher likelihood that it is instead a short infection of duration 30 days, rapidly followed by two relapses each causing blood-stage infections of duration 30 days. This inability to consistently identify the parameters for the dynamics of *P. vivax* infections is a major limitation of the method described here. It is possible that the model is mis-specified when applied to single-locus micro-satellite data with imperfect heterozygosity [19, 27]. This issue may be resolved by an update of the model to account for the genetic relatedness in an inoculum of sporozoites and the resulting relatedness between parasites from a primary infection and relapse [35], applied to genotyping data from more sophisticated technologies such as familial panels of microsatellites, amplicon sequencing, or genome-wide SNP panels [36, 37].

Despite the inability of the method to accurately estimate the duration of blood-stage *P. vivax* infection, the range of the estimates (24–29 days) can be considered consistent with the high proportion of individuals where no more than one consecutive sample was observed (Fig. 1). An alternative approach is to fit the model for *P. falciparum* infection dynamics to the *P. vivax* data. Although, this model does not account for relapses, estimates of the duration of blood-stage infection remain similarly short: 19 (95% CrI 16, 23) days in PNG and 34 (95% CrI 31, 39) days in Thailand. In contrast, data from malaria-therapy studies indicate that in non-immune adults blood-stage infections can persist for 60–70 days, and possibly even longer at sub-microscopic densities [4, 14]. Based on these observations, it is hypothesized that a primary *P. vivax* blood-stage infection may have a long duration (especially in malaria-naïve individuals), with relapses having a substantially shorter duration of infection. This concurs with other data from malaria-therapy studies demonstrating a reduction in parasite density between primary *P. vivax* infection and secondary infection with a homologous strain [14]. In individuals in the PNG and Thai cohorts with naturally-acquired immunity against *P. vivax* parasites, it is likely that the duration of the primary blood-stage infection is also reduced. Further evidence can be obtained from observations of *Plasmodium cynomolgi*: a simian malaria parasite that is genetically closely related to *P. vivax*, with many biological similarities most notably the presence of relapses. In controlled infections in rhesus macaques, untreated blood-stage infections due to *P. cynomolgi* had considerably shorter duration (approximately 10 days) than primary infection [38].

Addressing the knowledge gap on the duration of blood-stage *P. vivax* infections could have substantial clinical and public health value. The effectiveness of drug treatment strategies for reducing malaria transmission will depend on the duration of blood-stage infection: if the duration of infection is unknown, then the number of transmission events of gametocytes from humans to mosquitoes prevented by treatment can’t be assessed [39, 40]. If blood-stage *P. vivax* parasitaemia is sustained by multiple short infections, then clearing a single infection with a blood-stage drug such as artemisinin combination therapy (ACT) will cause very little reduction in transmission. In contrast, if the majority of *P. vivax* infections have longer duration expected, increased ACT treatment can be seen causing a reduction in transmission, similar to what has been seen for *P. falciparum* [41].

Another substantial limitation concerns the use of single-locus genotype markers. A single infectious mosquito bite may inoculate batches of parasites with different genotypes that may be unrelated or meiotic siblings that differ at the locus of interest [35, 37]. Furthermore, unrelated parasites may have the same genotype at the locus considered, although the expected heterozygosity of the genotyping markers is > 90%. It is also possible that a relapse may have a different genotype to its primary infection, but still be a half-sibling. To overcome these challenges, multiple-locus genotype markers are being developed, which should provide an even richer pattern of *P. falciparum* and *P. vivax* infection dynamics. In particular, the utilization of multi-locus genotype markers may resolve the issue of identifiability faced here by allowing a relapse to be distinguished from a primary blood-stage infection in cases where they are meiotic siblings.

There are a number of other limitations to the analytic methods applied here. Firstly, the role of heterogeneity and seasonality in exposure to mosquito bites are not accounted for, which is likely to increase the prevalence of co-infection with multiple *P. falciparum* and *P. vivax* genotypes. However, application of the methods to simulated data assuming heterogeneity or seasonality in exposure suggested that population-level parameters can still be estimated. Secondly, it is assumed that during co-infection, infections of different genotypes (both within and between species) are independent of one another. However it is likely that they interact due to induced innate immune responses and density-dependent regulation [42]. Thirdly, it is assumed relapses have a tropical phenotype [7, 8] and occur at a constant rate following primary infection, thus the authors do not account for the variation in relapse rate that may arise due to variation in the number of hypnozoites in the liver [29]. In particular, it is assumed that time to next relapse is exponentially distributed when the data may be better described by a more flexible distribution such as a Weibull distribution [17].

The predicted results may also depend on the frequency of sampling. To investigate this, alternate samples from individuals in the PNG cohort were excluded, and after repeating the analysis found only limited differences in the estimated population-level parameters (see Additional file 4: Fig. S4.2). The results may also depend on genotype detectability. Prior information from a series of experiments was relied upon where two blood samples were taken 24 h apart and the presence of genotypes compared between the two samples [43]. Higher parasite densities were found to increase genotype detectability, and increased multiplicity of infection (MOI) was found to reduce detectability. In addition, data from malaria-therapy studies has demonstrated a reduction in parasite density between primary *P. vivax* infection and secondary infection with a homologous strain [14]. Therefore, the assumption of constant genotype detectability may be a limitation of the method, leading to underestimation of the proportion of *P. vivax* relapses which may go undetected because of their lower blood-stage parasite densities. Finally, 100% specificity of genotype detection is also assumed. Imperfect specificity and density-dependent sensitivity will be investigated in future work.

Despite increasingly detailed observations of hypnozoites in vivo [44], the biological processes regulating hypnozoite activation remain unknown [9]. The combination of longitudinal data collection, genotyping of samples, and statistical modelling presented here provides a new approach for investigation of *P. vivax* recurrences. Whilst recurrences cannot be definitively classified into re-infections, recrudescences or relapses, the probability of each can be estimated, allowing a probabilistic relapse phenotype to be assigned to a *P. vivax* positive sample and, therefore, investigate the statistical association between relapses and epidemiological covariates.

The association between anti-malarial treatment and the incidence of *P. vivax* relapses in a population is complex, and depends on the duration of prophylactic protection provided by the drug [27]. After the initial treatment regimen of AL plus CQ in the placebo arm of the PNG cohort, a rebound in *P. vivax* genotype prevalence is observed (Fig. 6b). Similar findings have been reported by Tarning et al. [45], where a surge in the incidence of *P. vivax* relapses is predicted at 3, 6 and 9 weeks following treatment with dihydroartemisinin-piperaquine. It is hypothesized that this is due to the suppression by treatment prophylaxis of blood-stage parasites originating from relapses, with parasite densities rebounding after drug concentrations have waned.

The statistical model presented here provides a useful tool for detailed analysis of *P. falciparum* and *P. vivax* infection dynamics in longitudinal cohort studies. There are potentially important contributions to be made in the study of *P. vivax*, where infections can be assigned a probabilistic relapse phenotype allowing investigation of the association between epidemiological covariates and the incidence of relapses, facilitating a better understanding of the contribution of relapses to *P. vivax* transmission.

## Conclusion

The dynamics of *P. vivax* infections remain poorly understood, with no reliable estimates of the duration of blood-stage infection in populations in endemic areas with naturally-acquired immunity. *P. vivax* and *P. falciparum* infection dynamics can be investigated by analysing single-locus genotyping data from participants in longitudinal studies in malaria-endemic areas. A mathematical model where the times of infection and clearance of blood-stage parasites allows for the infection dynamics of *P. falciparum* to be estimated. However, such a model was not able to reliably estimate the parameters describing the infection dynamics of *P. vivax*. It is anticipated that further development of these methods and application to more informative genotyping markers will allow for a deeper understanding of *P. vivax* infection dynamics.

## Additional files

**Additional file 1.** Breakdown of malaria infections by genotype.

**Additional file 2.** Analysis of infection dynamics with augmented infection times.

**Additional file 3.** Model validation on simulated data.

**Additional file 4.** Supplementary results.

## References

[1] Ashley EA, White NJ (2014). The duration of *Plasmodium falciparum* infections. Malar J.

[2] Molineaux L, Diebner HH, Eichner M, Collins WE, Jeffery GM (2001). *Plasmodium falciparum* parasitaemia described by a new mathematical model. Parasitology.

[3] Recker M, Nee S, Bull PC, Kinyanjui S, Marsh K, Newbold S (2004). Transient cross-reactive immune responses can orchestrate antigenic variation in malaria. Nature.

[4] Kerlin DH, Gatton ML (2015). A simulation model of the within-host dynamics of *Plasmodium vivax* infection. Malar J.

[5] McQueen PG, McKenzie FE (2008). Host control of malaria infections: constraints on immune and erythropoietic response kinetics. PLoS Comp Biol.

[6] White NJ (2011). Determinants of relapse periodicity in *Plasmodium vivax* malaria. Malar J.

[7] Battle KE, Karhunen MS, Bhatt S, Gething PW, Howes RE, Golding N (2014). Geographical variation in *Plasmodium vivax* relapse. Malar J.

[8] Lover AA, Coker RJ (2013). Quantifying effect of geographic location on epidemiology of *Plasmodium vivax* malaria. Emerg Inf Dis.

[9] Mueller I, Galinski MR, Baird JK, Carlton JM, Kochar DK (2009). Key gaps in the knowledge of *Plasmodium vivax*, a neglected human malaria parasite. Lancet Inf Dis.

[10] Chen N, Auliff A, Rieckmann K, Gatton M, Cheng Q (2007). Relapses of *Plasmodium vivax* infection result from clonal hypnozoites activated at predetermined intervals. J Inf Dis.

[11] Robinson LJ, Wampfler R, Betuela I, Karl S, White MT, Li Wai Suen CSN (2015). Strategies for understanding and reducing the *Plasmodium vivax* and *Plasmodium ovale* hypnozoite reservoir in Papua New Guinean children: a randomised placebo-controlled trial and mathematical model. PLoS Med.

[12] Baird JK (2012). Reinventing primaquine for endemic malaria. Exp Opin Emerg Drugs.

[13] Bennett JW, Pybus BS, Yadava A, Tosh D, Sousa JC, McCarthy WF (2013). Primaquine failure and cytochrome P-450 2D6 in *Plasmodium vivax* malaria. New Eng J Med.

[14] Collins WE, Jeffery GM, Roberts JM (2004). A retrospective examination of reinfection of humans with *Plasmodium vivax*. Am J Trop Med Hyg.

[15] Collins WE, Jeffery GM (1999). A retrospective examination of the patterns of recrudescence in patients infected with *Plasmodium falciparum*. Am J Trop Med Hyg.

[16] Felger I, Maire M, Bretscher MT, Falk N, Tiaden A, Sama W (2012). The dynamics of natural *Plasmodium falciparum* infections. PLoS ONE.

[17] Ross A, Koepfli C, Schoepflin S, Timinao L, Siba P, Smith T (2016). The incidence and differential seasonal patterns of *Plasmodium vivax* primary infections and relapses in a cohort of children in Papua New Guinea. PLoS Negl Trop Dis.

[18] Koepfli C, Ross A, Kiniboro B, Smith TA, Zimmerman PA, Siba P (2011). Multiplicity and diversity of *Plasmodium vivax* infections in a highly endemic region in Papua New Guinea. PLoS Negl Trop Dis.

[19] Koepfli C, Mueller I, Marfurt J, Goroti M, Sie A, Oa O (2009). Evaluation of *Plasmodium vivax* genotyping markers for molecular monitoring in clinical trials. J Inf Dis.

[20] Smith T, Felger I, Fraser-Hurt N, Beck HP (1999). Effect of insecticide-treated bed nets on the dynamics of multiple *Plasmodium falciparum* infections. Trans Roy Soc Trop Med Hyg.

[21] Sama W, Owusu-Agyei S, Felger I, Vounatsou P, Smith T (2005). An immigration-death model to estimate the duration of malaria infection when detectability of the parasite is imperfect. Stat Med.

[22] Bretscher MT, Maire N, Chitnis N, Felger I, Owusu-Agyei S, Smith T (2011). The distribution of *Plasmodium falciparum* infection durations. Epidemics.

[23] Lin E, Kiniboro B, Gray L, Dobbie S, Robinson L, Laumaea A (2010). Differential patterns of infection and disease with *P. falciparum* and *P. vivax* in young Papua New Guinean children. PLoS ONE.

[24] Longley RJ, Reyes-Sandoval A, Montoya-Diaz E, Dunachie S, Kumpitak C, Nguitragool W (2016). Acquisition and longevity of antibodies to *Plasmodium vivax* preerythrocytic antigens in Western Thailand. Clin Vaccine Immunol.

[25] Wampfler R, Mwingira F, Javati S, Robinson L, Betuela I, Siba P (2013). Strategies for detection of *Plasmodium* species gametocytes. PLoS ONE.

[26] Rosanas-Urgell A, Mueller D, Betuela I, Barnadas C, Iga J, Zimmerman PA (2010). Comparison of diagnostic methods for the detection and quantification of the four sympatric *Plasmodium* species in field samples from Papua New Guinea. Malar J.

[27] Schoepflin S, Valsangiacomo F, Lin E, Kiniboro B, Mueller I, Felger I (2009). Comparison of *Plasmodium falciparum* allelic frequency distribution in different endemic settings by high-resolution genotyping. Malaria J.

[28] Mueller I, Widmer S, Michel D, Maraga S, McNamara DT, Kiniboro B (2009). High sensitivity detection of *Plasmodium* species reveals positive correlations between infections of different species, shifts in age distribution and reduced local variation in Papua New Guinea. Malar J.

[29] White MT, Karl S, Battle KE, Hay SI, Mueller I, Ghani AC (2014). Modelling the contribution of the hypnozoite reservoir to *Plasmodium vivax* transmission. Elife.

[30] White MT, Shirreff G, Karl S, Ghani AC, Mueller I (2016). Variation in relapse frequency and the transmission potential of *Plasmodium vivax* malaria. Proc Roy Soc B.

[31] Douglas NM, Nosten F, Ashley EA, Phaiphun L, van Vugt M, Singhasivanon P (2011). *Plasmodium vivax* recurrence following *falciparum* and mixed species malaria: risk factors and effect of antimalarial kinetics. Clin Inf Dis.

[32] Portugal S, Carret C, Recker M, Armitage AE, Goncalves LA, Epiphanio S (2011). Host-mediated regulation of superinfection in malaria. Nat Med.

[33] Soares IS, da Cunha MG, Silva MN, Souza JM, del Portillo HA, Rodrigues MM (1999). Longevity of naturally acquired antibody responses to the N- and C-terminal regions of *Plasmodium vivax* merozoite surface protein I. Am J Trop Med Hyg.

[34] Bretscher MT, Maire N, Felger I, Owusu-Agyei S, Smith T (2015). Asymptomatic *Plasmodium falciparum* infections may not be shortened by acquiring immunity. Malar J.

[35] Nkhoma SC, Nair S, Cheeseman IH, Rohr-Allegrini C, Singlam S, Nosten F (2012). Close kinship within multiple-genotype malaria parasite infections. Proc Roy Soc B.

[36] Koepfli C, Mueller I (2017). Malaria epidemiology at the clone level. Trends Parasitol.

[37] Bright AT, Manary MJ, Tewhey R, Arango EM, Wang T, Schork NJ (2014). A high resolution case study of a patient with recurrent *Plasmodium vivax* infections shows that relapses were caused by meiotic siblings. PLoS Negl Trop Dis.

[38] Joyner C, Moreno A, Meyer EVS, Cabrera-Mora M (2016). *Plasmodium cynomolgi* infections in rhesus macaques display clinical and parasitological features pertinent to modelling *vivax* malaria pathology and relapse infections. Malar J.

[39] Wampfler R, Hoffmann NE, Karl S, Betuela I, Kinboro B, Lorry L (2017). Effects of liver-stage clearance by primaquine on gametocyte carriage of *Plasmodium vivax* and *P. falciparum*. PLoS Negl Trop Dis.

[40] Johnston GL, Smith DL, Fidock DA (2013). Malaria’s missing number: calculating the human component of R_0_ by a within-host mechanistic model of *Plasmodium falciparum* infection and transmission. PLoS Comput Biol.

[41] Okell LC, Drakeley CJ, Bousema T, Whitty CJM, Ghani AC (2008). Modelling the impact of artemisinin combination therapy and long-acting treatments on malaria transmission intensity. PLoS Med.

[42] Bruce MC, Donnelly CA, Alpers MP, Galinski MR, Barnwell JW, Walliker D (2000). Cross-species interactions between malaria parasites in humans. Science.

[43] Koepfli C, Schoepflin S, Bretscher M, Lin E, Kiniboro B, Zimmerman PA (2011). How much remains undetected? Probability of molecular detection of human *Plasmodia* in the field. PLoS ONE.

[44] Mikolajczak SA, Vaughan AM, Kangwanrangsan N, Roobsoong W, Fishbaugher M, Yimamnuaychok N (2015). *Plasmodium vivax* liver stage development and hypnozoite persistence in human liver-chimeric mice. Cell Host Microbe.

[45] Tarning J, Thana P, Phyo AP, Lwin KM, Hanpithakpong W, Ashley EA (2014). Population pharmacokinetics and antimalarial pharmacodynamics of piperaquine in patients with *Plasmodium vivax* malaria in Thailand. CPT Pharmacometrics Syst Pharmacol.

