# Supplementary material for: Plasmodium vivax and Plasmodium falciparum infection dynamics: re-infections, recrudescences and relapses
Source: Malar J. 2018 Apr 17;17:170. doi: 10.1186/s12936-018-2318-1 (PMC5905131; doi:10.1186/s12936-018-2318-1)
Supplement: Supplementary file 1 — Additional file 1. Breakdown of malaria infections by genotype. [file 12936_2018_2318_MOESM1_ESM.docx]

***Plasmodium vivax* and *Plasmodium falciparum* infection dynamics: re-infections, recrudescences and relapses**

*Michael White, Stephan Karl, Cristian Koepfli, Rhea Longley, Natalie E. Hofmann, Rahel Wampfler, Ingrid Felger, Tom Smith, Wang Nguitragool, Jetsumon Sattabongkot, Leanne Robinson, Azra Ghani, Ivo Mueller*

**Additional file 1: Breakdown of malaria infections by genotype**

**1.1. *P. falciparum* samples from Papua New Guinea**

Table S1.1: The 20 most commonly detected *P. falciparum* genotypes in Maprik, Papua New Guinea. N_pos_ denotes the number of participants with at least one sample positive for that genotype. The genotype prevalence (genPr) denotes the percentage of PCR-positive samples positive for that genotype. The pattern count provides a breakdown of the frequency of infections with varying numbers of consecutively positive samples. Note that a single individual may have a more complex pattern such 01010 which would count as two instances of 010. The 14 most common genotypes included in the model fitting are shaded grey.

|  | ***P. falciparum* placebo arm (*n* = 257)** | | | | | | | ***P. falciparum* primaquine arm (*n* = 247)** | | | | | | |
| --- | --- | --- | --- | --- | --- | --- | --- | --- | --- | --- | --- | --- | --- | --- |
|  |  |  | **pattern count** | | | | |  |  | **pattern count** | | | | |
| **genotype** | **N_pos_** | **genPr** | **010** | **0110** | **01110** | **011110** | **0111110** | **N_pos_** | **genPr** | **010** | **0110** | **01110** | **011110** | **0111110** |
| F359.1 | 33 | 27.2% | 22 | 9 | 4 | 4 | 0 | 29 | 23.3% | 27 | 7 | 3 | 3 | 0 |
| F371.6 | 21 | 10.2% | 22 | 2 | 0 | 1 | 0 | 20 | 9.4% | 19 | 3 | 0 | 0 | 0 |
| D415.9 | 15 | 6.5% | 14 | 1 | 1 | 0 | 0 | 12 | 6.0% | 10 | 3 | 0 | 0 | 0 |
| D217.7 | 14 | 9.5% | 12 | 2 | 2 | 0 | 0 | 7 | 6.0% | 6 | 5 | 0 | 0 | 0 |
| F336.9 | 10 | 6.1% | 10 | 1 | 2 | 0 | 0 | 11 | 7.5% | 12 | 2 | 0 | 1 | 0 |
| D270.9 | 8 | 3.1% | 9 | 0 | 0 | 0 | 0 | 6 | 3.4% | 6 | 0 | 1 | 0 | 0 |
| F517.9 | 7 | 2.7% | 6 | 1 | 0 | 0 | 0 | 7 | 3.8% | 6 | 2 | 0 | 0 | 0 |
| D336.6 | 6 | 4.1% | 7 | 1 | 1 | 0 | 0 | 2 | 1.1% | 1 | 1 | 0 | 0 | 0 |
| D381.2 | 5 | 3.1% | 5 | 2 | 0 | 0 | 0 | 3 | 1.5% | 2 | 1 | 0 | 0 | 0 |
| D300.7 | 4 | 2.7% | 3 | 0 | 0 | 0 | 0 | 3 | 1.9% | 3 | 1 | 0 | 0 | 0 |
| D334.4 | 4 | 2.0% | 6 | 0 | 0 | 0 | 0 | 2 | 1.5% | 2 | 1 | 0 | 0 | 0 |
| D339.6 | 4 | 2.0% | 2 | 2 | 0 | 0 | 0 | 2 | 0.8% | 2 | 0 | 0 | 0 | 0 |
| D341.7 | 4 | 2.7% | 5 | 0 | 1 | 0 | 0 | 3 | 1.9% | 5 | 0 | 0 | 0 | 0 |
| D375.9 | 4 | 1.7% | 3 | 1 | 0 | 0 | 0 | 2 | 1.9% | 3 | 1 | 0 | 0 | 0 |
| F407.6 | 4 | 2.7% | 4 | 2 | 0 | 0 | 0 | 2 | 1.1% | 1 | 1 | 0 | 0 | 0 |
| D347.6 | 3 | 1.0% | 3 | 0 | 0 | 0 | 0 | 3 | 1.1% | 3 | 0 | 0 | 0 | 0 |
| D364.3 | 3 | 2.4% | 3 | 2 | 0 | 0 | 0 | 1 | 0.4% | 1 | 0 | 0 | 0 | 0 |
| D422.3 | 3 | 1.7% | 3 | 1 | 0 | 0 | 0 | 2 | 0.8% | 2 | 0 | 0 | 0 | 0 |
| D330.0 | 2 | 1.4% | 0 | 2 | 0 | 0 | 0 | 2 | 1.6% | 1 | 0 | 1 | 0 | 0 |
| F298.7 | 2 | 1.0% | 1 | 1 | 0 | 0 | 0 | 0 | 0.0% | 0 | 0 | 0 | 0 | 0 |

**1.2. *P. vivax* samples from Papua New Guinea**

Table S1.2: The 31 most commonly detected *P. vivax* genotypes in Maprik, Papua New Guinea. N_pos_ denotes the number of participants with at least one sample positive for that genotype. The genotype prevalence (genPr) denotes the percentage of PCR-positive samples positive for that genotype. The pattern count provides a breakdown of the frequency of infections with varying numbers of consecutively positive samples. Note that a single individual may have a more complex pattern such 01010 which would count as two instances of 010. The 14 most common genotypes included in the model fitting are shaded grey.

|  | ***P. vivax* placebo arm (*n* = 257)** | | | | | | | ***P. vivax* primaquine arm (*n* = 247)** | | | | | | |
| --- | --- | --- | --- | --- | --- | --- | --- | --- | --- | --- | --- | --- | --- | --- |
|  |  |  | **pattern count** | | | | |  |  | **pattern count** | | | | |
| **genotype** | **N_pos_** | **genPr** | **010** | **0110** | **01110** | **011110** | **0111110** | **N_pos_** | **genPr** | **010** | **0110** | **01110** | **011110** | **0111110** |
| M262.9 | 106 | 30.5% | 119 | 24 | 9 | 2 | 0 | 42 | 33.2% | 41 | 9 | 3 | 1 | 0 |
| M236.2 | 90 | 21.4% | 104 | 16 | 2 | 0 | 0 | 25 | 14.8% | 27 | 2 | 1 | 0 | 0 |
| M273.9 | 62 | 14.2% | 74 | 10 | 0 | 0 | 0 | 18 | 10.5% | 22 | 1 | 0 | 0 | 0 |
| M256.6 | 53 | 9.8% | 59 | 3 | 0 | 0 | 0 | 17 | 10.5% | 17 | 2 | 1 | 0 | 0 |
| M316.3 | 39 | 7.2% | 38 | 5 | 0 | 0 | 0 | 9 | 4.3% | 8 | 1 | 0 | 0 | 0 |
| M319.2 | 32 | 6.8% | 34 | 4 | 1 | 0 | 0 | 5 | 3.1% | 3 | 2 | 0 | 0 | 0 |
| M366.2 | 26 | 6.2% | 32 | 3 | 1 | 0 | 0 | 11 | 7.9% | 13 | 0 | 0 | 0 | 1 |
| M250.5 | 24 | 4.8% | 26 | 3 | 0 | 0 | 0 | 7 | 4.8% | 8 | 0 | 1 | 0 | 0 |
| M313.1 | 18 | 3.3% | 18 | 2 | 0 | 0 | 0 | 6 | 3.1% | 7 | 0 | 0 | 0 | 0 |
| M325.2 | 16 | 3.2% | 15 | 3 | 0 | 0 | 0 | 5 | 2.2% | 5 | 0 | 0 | 0 | 0 |
| M270.9 | 10 | 2.0% | 8 | 1 | 1 | 0 | 0 | 2 | 0.9% | 2 | 0 | 0 | 0 | 0 |
| M306.9 | 9 | 2.4% | 9 | 0 | 0 | 0 | 0 | 6 | 2.6% | 6 | 0 | 0 | 0 | 0 |
| M337.2 | 8 | 1.5% | 8 | 1 | 0 | 0 | 0 | 1 | 0.4% | 1 | 0 | 0 | 0 | 0 |
| M370.8 | 8 | 1.5% | 8 | 1 | 0 | 0 | 0 | 1 | 0.4% | 2 | 0 | 0 | 0 | 0 |
| M310.2 | 6 | 1.2% | 6 | 1 | 0 | 0 | 0 | 4 | 1.7% | 4 | 0 | 0 | 0 | 0 |
| M328.3 | 6 | 1.2% | 4 | 2 | 0 | 0 | 0 | 0 | 0.0% | 0 | 0 | 0 | 0 | 0 |
| M277.7 | 5 | 0.9% | 6 | 0 | 0 | 0 | 0 | 3 | 2.2% | 5 | 0 | 0 | 0 | 0 |
| M346.7 | 5 | 0.9% | 4 | 1 | 0 | 0 | 0 | 0 | 0.0% | 0 | 0 | 0 | 0 | 0 |
| M322.2 | 5 | 0.8% | 5 | 0 | 0 | 0 | 0 | 1 | 1.3% | 3 | 0 | 0 | 0 | 0 |
| M340.4 | 4 | 1.1% | 3 | 2 | 0 | 0 | 0 | 0 | 0.0% | 0 | 0 | 0 | 0 | 0 |
| M294.6 | 3 | 0.5% | 3 | 0 | 0 | 0 | 0 | 1 | 1.3% | 0 | 0 | 1 | 0 | 0 |
| M300.7 | 3 | 0.6% | 4 | 0 | 0 | 0 | 0 | 0 | 0.0% | 0 | 0 | 0 | 0 | 0 |
| M368.1 | 3 | 0.5% | 3 | 0 | 0 | 0 | 0 | 1 | 0.4% | 1 | 0 | 0 | 0 | 0 |
| M373.5 | 3 | 0.5% | 3 | 0 | 0 | 0 | 0 | 0 | 0.0% | 0 | 0 | 0 | 0 | 0 |
| M359.5 | 3 | 0.5% | 3 | 0 | 0 | 0 | 0 | 1 | 0.4% | 1 | 0 | 0 | 0 | 0 |
| M288.5 | 2 | 0.3% | 2 | 0 | 0 | 0 | 0 | 4 | 2.6% | 3 | 0 | 1 | 0 | 0 |
| M331.2 | 2 | 0.6% | 1 | 0 | 1 | 0 | 0 | 0 | 0.00% | 0 | 0 | 0 | 0 | 0 |
| M285.6 | 2 | 0.8% | 3 | 1 | 0 | 0 | 0 | 4 | 2.2% | 5 | 0 | 0 | 0 | 0 |
| M357.0 | 2 | 0.3% | 3 | 0 | 0 | 0 | 0 | 1 | 0.4% | 1 | 0 | 0 | 0 | 0 |
| M297.5 | 2 | 0.8% | 2 | 2 | 0 | 0 | 0 | 1 | 0.4% | 1 | 0 | 0 | 0 | 0 |
| M280.5 | 2 | 0.5% | 1 | 1 | 0 | 0 | 0 | 0 | 0.00% | 0 | 0 | 0 | 0 | 0 |

**1.3. *P. falciparum* samples from Thailand**

Table S1.3: The 17 most commonly detected *P. falciparum* genotypes in Thailand. N_pos_ denotes the number of participants with at least one sample positive for that genotype. The genotype prevalence (genPr) denotes the percentage of PCR-positive samples positive for that genotype. The pattern count provides a breakdown of the frequency of infections with varying numbers of consecutively positive samples. Note that a single individual may have a more complex pattern such 01010 which would count as two instances of 010. The 14 most common genotypes included in the model fitting are shaded grey.

|  | ***P. falciparum* (*n* = 999)** | | | | | | |
| --- | --- | --- | --- | --- | --- | --- | --- |
|  |  |  | **pattern count** | | | | |
| **genotype** | **N_pos_** | **genPr** | **010** | **0110** | **01110** | **011110** | **0111110** |
| Pf.B335.11 | 10 | 27.8% | 14 | 4 | 0 | 0 | 0 |
| Pf.G185.81 | 3 | 3.8% | 3 | 0 | 0 | 0 | 0 |
| Pf.G397.43 | 3 | 3.8% | 3 | 0 | 0 | 0 | 0 |
| Pf.G409.91 | 3 | 12.7% | 2 | 2 | 0 | 1 | 0 |
| Pf.B371.18 | 2 | 13.9% | 1 | 1 | 1 | 0 | 1 |
| Pf.G434.11 | 2 | 7.6% | 2 | 0 | 0 | 1 | 0 |
| Pf.B261.17 | 1 | 1.3% | 1 | 0 | 0 | 0 | 0 |
| Pf.B297.73 | 1 | 1.3% | 1 | 0 | 0 | 0 | 0 |
| Pf.G208.95 | 1 | 1.3% | 1 | 0 | 0 | 0 | 0 |
| Pf.G255.15 | 1 | 1.3% | 1 | 0 | 0 | 0 | 0 |
| Pf.G336.63 | 1 | 3.8% | 0 | 0 | 1 | 0 | 0 |
| Pf.G341.26 | 1 | 2.5% | 2 | 0 | 0 | 0 | 0 |
| Pf.G345.18 | 1 | 2.5% | 2 | 0 | 0 | 0 | 0 |
| Pf.G347.09 | 1 | 2.5% | 0 | 1 | 0 | 0 | 0 |
| Pf.G386.66 | 1 | 1.3% | 1 | 0 | 0 | 0 | 0 |
| Pf.G407.21 | 1 | 3.8% | 1 | 1 | 0 | 0 | 0 |
| Pf.G421.93 | 1 | 6.3% | 0 | 0 | 0 | 0 | 1 |

**1.4. *P. vivax* samples from Thailand**

Table S1.4: The 24 most commonly detected *P. vivax* genotypes in Thailand. N_pos_ denotes the number of participants with at least one sample positive for that genotype. The genotype prevalence (genPr) denotes the percentage of PCR-positive samples positive for that genotype. The pattern count provides a breakdown of the frequency of infections with varying numbers of consecutively positive samples. Note that a single individual may have a more complex pattern such 01010 which would count as two instances of 010. The 14 most common genotypes included in the model fitting are shaded grey.

|  | ***P. vivax* (*n* = 999)** | | | | | | |
| --- | --- | --- | --- | --- | --- | --- | --- |
|  |  |  | **pattern count** | | | | |
| **genotype** | **N_pos_** | **genPr** | **010** | **0110** | **01110** | **011110** | **0111110** |
| Pv.G262.71 | 62 | 39.1% | 78 | 17 | 8 | 3 | 3 |
| Pv.G256.73 | 52 | 38.3% | 62 | 17 | 9 | 3 | 0 |
| Pv.B189.04 | 21 | 8.8% | 26 | 4 | 1 | 1 | 0 |
| Pv.G307.14 | 17 | 8.2% | 24 | 2 | 2 | 1 | 0 |
| Pv.B204.7 | 16 | 6.0% | 18 | 5 | 0 | 0 | 0 |
| Pv.B231.81 | 11 | 3.9% | 14 | 2 | 0 | 0 | 0 |
| Pv.B216.25 | 10 | 3.4% | 16 | 0 | 0 | 0 | 0 |
| Pv.B196.79 | 9 | 4.1% | 8 | 4 | 1 | 0 | 0 |
| Pv.B224.22 | 9 | 5.6% | 11 | 3 | 3 | 0 | 0 |
| Pv.B247.29 | 9 | 2.6% | 12 | 0 | 0 | 0 | 0 |
| Pv.B212.61 | 8 | 6.0% | 12 | 3 | 2 | 1 | 0 |
| Pv.B255.15 | 8 | 1.9% | 7 | 1 | 0 | 0 | 0 |
| Pv.B258.87 | 7 | 3.4% | 6 | 1 | 0 | 0 | 0 |
| Pv.B177.14 | 6 | 2.6% | 10 | 1 | 0 | 0 | 0 |
| Pv.B239.88 | 6 | 2.8% | 8 | 1 | 1 | 0 | 0 |
| Pv.B266.87 | 6 | 2.2% | 6 | 2 | 0 | 0 | 0 |
| Pv.B180.97 | 5 | 2.6% | 5 | 1 | 0 | 0 | 1 |
| Pv.B208.49 | 5 | 2.6% | 7 | 1 | 1 | 0 | 0 |
| Pv.B227.98 | 5 | 2.2% | 4 | 1 | 0 | 1 | 0 |
| Pv.B243.44 | 5 | 1.1% | 5 | 0 | 0 | 0 | 0 |
| Pv.B251.12 | 5 | 3.0% | 6 | 0 | 1 | 0 | 1 |
| Pv.B219.92 | 4 | 3.0% | 3 | 2 | 0 | 0 | 0 |
| Pv.B369.4 | 4 | 0.9% | 4 | 0 | 0 | 0 | 0 |
| Pv.G250.63 | 4 | 2.6% | 3 | 3 | 1 | 0 | 0 |
