# Supplementary material for: Plasmodium vivax and Plasmodium falciparum infection dynamics: re-infections, recrudescences and relapses
Source: Malar J. 2018 Apr 17;17:170. doi: 10.1186/s12936-018-2318-1 (PMC5905131; doi:10.1186/s12936-018-2318-1)
Supplement: Supplementary file 2 — Additional file 2. Analysis of infection dynamics with augmented infection times. [file 12936_2018_2318_MOESM2_ESM.docx]

***Plasmodium vivax* and *Plasmodium falciparum* infection dynamics: re-infections, recrudescences and relapses**

*Michael White, Stephan Karl, Cristian Koepfli, Rhea Longley, Natalie E. Hofmann, Rahel Wampfler, Ingrid Felger, Tom Smith, Wang Nguitragool, Jetsumon Sattabongkot, Leanne Robinson, Azra Ghani, Ivo Mueller*

**Additional file 2:** **Analysis of infection dynamics with augmented infection times**

**2.1. Overview**

For each participant followed longitudinally for infection with a given genotype the data can be described schematically as shown in Figure S2.1. Sample *j* measured at time point$\tau^{j}$can be either positive$\left(x^{j}=1\right)$or negative$\left(x^{j}=0\right)$. The approach of the triplet model [[1](#_ENREF_1)] is to cut this data up into groups of three consecutive samples (e.g. (0,1,1), (1,1,0), (1,0,1), (0,1,0), (1,0,0), (0,0,0),(0,0,1)) and then analyse the relative frequency of each triplet.

Figure S2.1: Schematic of infection dynamics of a single genotype in a participant based on longitudinal samples at times$\tau^{j}$. Example times for the acquisition$\left(T_{\inf}\right)$ and clearance $\left(T_{\text{clear}}\right)$of two infections are shown. The exact values of$T_{\inf}$and $T_{\text{clear}}$ are unknown and hence are treated as parameters for data augmentation.

Here we pursue a slightly different approach to the triplet model where we estimate the time of acquisition and clearance of each infection. For example, in Figure S2.1 the sequence of positive and negative samples could be due to two separate infections. The first infection is acquired at time$T_{\inf}^{1}$in the interval$\left[\tau^{1},\tau^{2}\right]$ and clears at time$T_{\text{clear}}^{1}$in the interval $\left[\tau^{5},\tau^{6}\right]$. This means that infection was present at time $\tau^{4}$ but was missed due to imperfect detectability of the genotyping method. We then have a second infection acquired at time$T_{\inf}^{2}$ and clearing at time$T_{\text{clear}}^{2}$.

Importantly, the acquisition and clearance times in Figure S2.1 are only one of many combinations that can produce the observed pattern of positive and negative samples. The true times of acquisition and clearance are unknowable. They are instead treated as parameters to be augmented. Although we don’t know the exact value of $T_{\inf}^{1}$we can estimate its distribution. We know for certain that $T_{\inf}^{1}<\tau^{2}$ when infection was first detected. We also know that $T_{\inf}^{1}$ must have occurred after the period of prophylactic protection (in grey).

The example in Figure S2.1 is from a single genotype in a single individual. When we combine patterns from multiple individuals and multiple genotypes we can obtain information on what infection dynamics look like on a population level. For example, if we see lots of 00100 patterns this would indicate short durations of blood-stage infection (or low dectability). If we see lots of 01110 patterns this would indicate longer durations of blood-stage infection and higher detectability.

**2.2. *P. falciparum* infection dynamics without treatment**

We first consider a model for the presence or absence of blood-stage infections in the absence of anti-malarial treatment. Relapses are not accounted for, thus the model is appropriate for analysing *P. falciparum* data. Table S2.15 gives an overview of the key parameters and definitions.

Table S2.1: Overview of key parameters for a model of *P. falciparum* infection dynamics without treatment

| **parameter** | **description** | **notes** |
| --- | --- | --- |
| *data* | | |
| $\tau^{jgn}$ | time of sample *j* of genotype *g* in participant *n* |  |
| $x^{jgn}$ | status of sample *j* of genotype *g* in participant *n* |  |
|  |  |  |
| *data augmented parameters* | | |
| $T_{\inf}^{kgn}$ | acquisition time of *k*^th^ infection of genotype *g* in participant *n* | estimated |
| $T_{\text{clear}}^{kgn}$ | clearance time of *k*^th^ infection of genotype *g* in participant *n* | estimated |
| $d_{\text{BS}}^{kgn}$ | duration of *k*^th^ infection of genotype *g* in participant *n* | $d_{\text{BS}}^{kgn}=T_{\text{clear}}^{kgn}-T_{\inf}^{kgn}$ |
|  |  |  |
| *genotype-specific force of infection* | | |
| $\lambda^{g}$ | force of infection of genotype *g* | estimated |
|  |  |  |
| *duration of infection and detectability (across all genotypes)* | | |
| *d_BS_* | average duration of blood-stage infection | estimated |
| *d*_BS,min_ | minimum duration of blood-stage infection | fixed (5 days) |
| κ | Weibull shape parameter | estimated |
| ω | Weibull scale parameter | $\omega=\frac{d_{BS}-d_{BS,\min}}{\Gamma\left(1+1/\kappa\right)}$ |
| *q* | detectability: probability of detecting genotype | estimated |

We define notation for the data as follows. For genotype *g* in participant *n*, we have$J^{gn}$samples at time points$\tau^{jgn}$. Let$x^{jgn}$denote the status of the sample at time$\tau^{jgn}$, such that

$$x^{jgn}=\left\{\begin{matrix} 0 & \text{genotype }g\text{ not detected at }\tau^{jgn} \\ 1 & \text{genotype }g\text{ detected at }\tau^{jgn} \end{matrix}\right.$$

We consider the infection dynamics due to $K^{gn}$blood-stage infections arising from separate mosquito bites. We assume that infection *k* is acquired at time $T_{\inf}^{kgn}$ and clears at time$T_{\text{clear}}^{kgn}$, giving rise to a blood-stage infection of duration$d_{\text{BS}}^{kgn}=T_{\inf}^{kgn}-T_{\text{clear}}^{kgn}$ . The exact values of $T_{\inf}^{kgn}$ and$T_{\text{clear}}^{kgn}$are unknown and hence we treat them as parameters for data augmentation.

The method for detection of genotypes has imperfect detectability due to variation in blood-stage parasite densities and other factors. We denote *q* to be the sensitivity of genotype detection. The likelihood of observing sample$x^{jgn}$ at time point$\tau^{jgn}$ from infection *k* accounting for the sensitivity of detection is given by:

$$\left(\underbrace{\mathbf{1}\left(T_{\inf}^{kgn}<\tau^{jgn}<T_{\text{clear}}^{kgn}\right)\left(1-q\right)^{1-x^{jgn}}q^{x^{jgn}}}_{\text{sample }j\text{ falls within infection }k}+\underbrace{1-\mathbf{1}\left(T_{\inf}^{kgn}<\tau^{jgn}<T_{\text{clear}}^{kgn}\right)}_{\text{sample }j\text{ falls outside infection }k}\right)$$

where is an indicator function. We can then calculate the sampling likelihood for infection *k* by multiplying over all samples$J^{gn}$

$$\mathbf{S}\left(T_{\inf}^{kgn},T_{\text{clear}}^{kgn}\right)=\prod_{j=1}^{J^{gn}} \left(\underbrace{\mathbf{1}\left(T_{\inf}^{kgn}<\tau^{jgn}<T_{\text{clear}}^{kgn}\right)\left(1-q\right)^{1-x^{jgn}}q^{x^{jgn}}}_{\text{sample }j\text{ falls within infection }k}+\underbrace{1-\mathbf{1}\left(T_{\inf}^{kgn}<\tau^{jgn}<T_{\text{clear}}^{kgn}\right)}_{\text{sample }j\text{ falls outside infection }k}\right)$$

The acquisition times $T_{\inf}^{kgn}$ will depend on the genotype-specific force of infection$\lambda^{g}$. If we assume the force of infection is constant in time and constant across individuals then the likelihood of acquisition times can be described by an exponential distribution:

$$\mathbf{P}\left(T_{\inf}^{kgn}\right)=\lambda^{g}e^{-\lambda^{g}\left(T_{\inf}^{kgn}-T_{\text{clear}}^{(k-1)gn}\right)}$$

Note that we assume there is no seasonality in the genotype-specific force of infection. In cases where the data indicate seasonality in new infections, an average force of infection will be estimated. The time between the clearance of infection$k-1$ and the acquisition of infection$k$ is exponentially distributed. Note that in the PNG cohort which had a treatment re-infection design, participants are clear of blood-stage parasites once the period of prophylaxis at the start of follow-up has ended. It is thus convenient to set$T_{\text{clear}}^{0gn}=\tau^{0gn}+1+T_{\text{prop},0}$ . It is assumed that treatment is administered one day after sample $\tau^{0gn}$ and provides prophylactic protection against new infection for a duration $T_{\text{prop},0}$ days.

In practice, to ensure numerical stability of the Metropolis-Hastings MCMC algorithm, we need to impose an upper limit on the acquisition times$T_{\text{inf}}^{kgn}$ . Denote this$T_{\text{max}}$ . This causes the exponential distribution above to be truncated, and hence we must normalise the likelihood as follows:

$$\mathbf{P}\left(T_{\inf}^{kgn}\right)=\frac{\lambda^{g}e^{-\lambda^{g}\left(T_{\inf}^{kgn}-T_{\text{clear}}^{(k-1)gn}\right)}}{1-e^{-\lambda^{g}\left(T_{\text{max}}-T_{\text{clear}}^{(k-1)gn}\right)}}$$

For$k>0$ , the clearance times $T_{\text{clear}}^{kgn}$ will depend on the duration of blood-stage infection$d_{\text{BS}}^{kgn}$. We assume that the duration of blood-stage infection can be described by an offset Weibull distribution

$$\mathbf{P}\left(d_{\text{BS}}^{kgn}\right)=\frac{\kappa}{\omega}\left(\frac{d_{\text{BS}}^{kgn}-d_{BS,\min}}{\omega}\right)^{\kappa-1}e^{-\left(\frac{d_{\text{BS}}^{kgn}-d_{BS,\min}}{\omega}\right)^{\kappa}}$$

where $\kappa>0$ is the Weibull shape parameter and $\omega=\frac{d_{BS}-d_{BS,\min}}{\Gamma\left(1+1/\kappa\right)}$ is the Weibull scale parameter. $d_{BS}$is the mean duration of blood-stage infection across all participants and across all genotypes. It is a population-level parameter, in contrast to $d_{\text{BS}}^{kgn}$ which is an individual-level parameter corresponding only to infection$k$of genotype$g$ in participant$n$.

The Weibull distribution has previously been used to model the duration of *P. falciparum* blood-stage infections [[2](#_ENREF_2)]. It is an appropriate choice of distribution as it can capture a range of qualitatively different distributions of durations. Note that when$\kappa<1$the mode of the distribution (without the offset) is zero. An increasing proportion of the probability mass is close to zero for smaller$\kappa$. This results in a high proportion of infections of very short duration, e.g. < 3 days. It is unclear if this is biologically meaningful, and even if it were, whether the data are powered to detect such infections. We thus choose to offset the Weibull distribution by imposing some minimum duration of infection$d_{BS,\min}$ . Examples of Weibull distributions with offset = 5 days are shown in Figure S2.2 for a range of$\kappa$ .


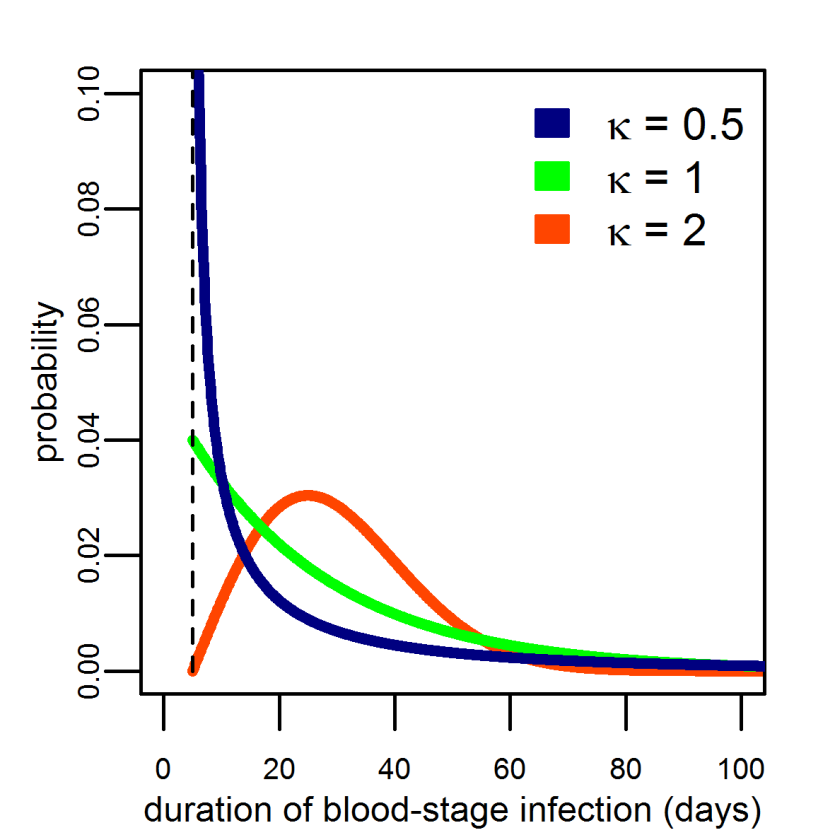


Figure S2.2: Weibull distribution for the duration of blood-stage infections. The distribution has been offset to ensure a minimum duration of infection of *d*_BS,min_ = 5 days (dashed line). The mean duration of infection in this example is 30 days. Different Weibull shape parameters give qualitatively different distributions. When *κ* = 1 the Weibull distribution simplifies to an exponential distribution. When *κ* < 1 the modal value of the distribution is *d*_BS,min_.

The three components of the likelihood in equations (3), (5) and (6) can be combined to provide an expression for the likelihood of the data augmented parameters $T_{\inf}^{kgn}$ and$T_{\text{clear}}^{kgn}$given the observed samples$x^{jgn}$and the global parameters$q$,$d_{BS}$ ,$\kappa$ and $\lambda^{g}$.

$$L^{gn}=\prod_{k=1}^{K} \left(\underbrace{\mathbf{P}\left(T_{\inf}^{kgn}\right)}_{\text{infection likelihood}}\underbrace{\mathbf{P}\left(d_{\text{BS}}^{kgn}\right)}_{\text{duration likelihood}}\underbrace{\mathbf{S}\left(T_{\inf}^{kgn},T_{\text{clear}}^{kgn}\right)}_{\text{sampling likelihood}}\right)$$

Finally the total likelihood can be obtained by multiplying the likelihoods for all genotypes from all participants

$$L=\prod_{n=1}^{N} \prod_{g=1}^{G} L^{gn}$$

**2.3. *P. falciparum* infection dynamics with treatment**

Anti-malaria treatment administered during the period of longitudinal follow-up will clear blood-stage infections, resulting in the observation of shorter infections than would be expected under conditions in the absence of treatment. Failure to account for the effects of treatment may lead to under-estimation of the duration of blood-stage infections. Treatment is assumed to have two effects: (i) clearance of existing blood-stage infections; and (ii) provision of a period of prophylactic protection. We have data on the times at which individuals were provided with anti-malarials; denote the time of *i*^th^ treatment as$\xi^{i}$.

The schematic in Figure S2.3 gives an overview of how treatment can be incorporated into the data augmentation framework described previously. With treatment, the duration of infection $k=2$is$d_{\text{BS}}^{2}=T_{\text{clear}}^{2}-T_{\inf}^{2}$ . However, if there were no treatment, it is likely that there would be a longer duration of infection$d_{\text{BS}}^{2\dagger}=T_{\text{clear}}^{2\dagger}-T_{\inf}^{2}$ .

Our approach is to sample the data augmented parameters $T_{\inf}^{k}$ and$T_{\text{clear}}^{k\dagger}$during the MCMC update. If a treatment is recorded to have been provided at time $\xi^{i}$ between $T_{\inf}^{k}$ and$T_{\text{clear}}^{k\dagger}$then we adjust the clearance time such that $T_{\text{clear}}^{k}=\xi^{i}$

$$T_{\text{clear}}^{k}=\left\{\begin{matrix} \xi^{i} & \exists\text{ }i\text{ }T_{\inf}^{k}<\xi^{i}<T_{\text{clear}}^{k\dagger} \\ T_{\text{clear}}^{k\dagger} & \text{otherwise} \end{matrix}\right.$$

Table S2.2 provides an overview of the key parameters and definitions.

Figure S2.3: Schematic of infection dynamics of a single genotype in a participant accounting for the effects of treatment prophylaxis. In the absence of treatment, the second infection would clear at time$T_{\text{clear}}^{2\dagger}$. However treatment can reduce the duration of infection, causing clearance at time$T_{\text{clear}}^{2}$.

Table S2.2: Additional parameters to account for the effects of treatment.

| **parameter** | **description** | **notes** |
| --- | --- | --- |
| *data* | | |
| $\xi^{in}$ | time of treatment *i* in participant *n* |  |
|  |  |  |
| *data augmented parameters* | | |
| $T_{\text{clear}}^{kgn\dagger}$ | clearance time of *k*^th^ infection of genotype *g* in participant *n* | estimated |
| $d_{\text{BS}}^{kgn\dagger}$ | duration of *k*^th^ infection of genotype *g* in participant *n* | $d_{\text{BS}}^{kgn\dagger}=T_{\text{clear}}^{kgn\dagger}-T_{\inf}^{kgn}$ |
|  |  |  |
| *treatment prophylaxis* | | |
| *T*_prop_ | duration of treatment prophylaxis following AL | fixed (10 days) |
| *T*_prop,0_ | duration of treatment prophylaxis at start | fixed (24 days) |

**2.4. *P. vivax* infection dynamics with hypnozoite clearance at start of follow-up**

**Model of relapses**

The models described above do not contain an explicit representation of relapses. They can still be applied to *P. vivax* data, but they fail to capture some important elements of the biology. For example, if relapses are not accounted for, then a 101 pattern could be due to either a single infection with failure to detect the second sample (i.e. a recrudescence), or a second new infection from a mosquito bite (i.e. a re-infection). However, for *P. vivax* there is an important third possibility: clearance of primary infection followed by a relapse.

We must first describe how hypnozoite infection of the liver and relapses can be described mathematically. We follow the approach of White *et al* [[3](#_ENREF_3)] where liver-stage infection is incorporated into a Ross-MacDonald framework of the transmission of *P. vivax* parasites between humans and mosquitoes. In Figure S2.4, $S$denotes individuals without blood-stage infection (i.e. susceptible) and $I$denotes individuals with blood-stage infection. The sub-script 0 denotes that an individual is free from liver-stage hypnozoites, and the subscript *L* dentoes that the individual is infected with liver-stage hypnzoites.

Figure S2.4: Compartmental representation of the incorporation of relapses into a Ross-MacDonald model after White *et al* [[3](#_ENREF_3)].

In the system corresponding to the compartmental model in Figure S4: all individuals can become infected from mosquito bites at rate$\lambda$; individuals with hypnozoites (sub-script *L*) can relapse at rate$f$; individuals can clear liver-stage hypnozoites at rate$\gamma_{L}$; and individuals can clear blood-stage parasites at rate$r$.

Individuals infected with both blood-stage and liver-stage parasites can still experience relapses, however in practice these are unlikely to be dectable because of existing circulating blood-stage parasites. The standard assumption in compartmental models is that all waiting times are exponential. Thus the duration of blood-stage infection is related to the rate of recovery as follows$d_{BS}=1/r$. For the purposes below, this restrictive assumption isn’t always necessary and we can asume that infection durations can be described by more flexible Weibull distributions.

A description of the epidemiology of *P. vivax* requires the specification of two more parameters as outlined in Table S2.5.

Table S2.5: Parameters describing the epidemiology of *P. vivax* relapses.

| **parameter** | **description** | **notes** |
| --- | --- | --- |
| *relapses* | | |
| *f* | relapse frequency (⬄ 1/time to next relapse) | estimated |
| *γ_L_* | rate of clearance of liver-stage infection | estimated |
| *h* | number of relapses per primary infection | *h = f*/*γ_L_* |

**Time to next infection**

Here we describe how the above framework can be extended to account for relapses characteristic of *P. vivax* as depicted in Figure S2.4. Each new infection $T_{\inf}^{k}$may be due to reinfection with force of infection$\lambda$(we drop the superscript *g* for specification of genotype for ease of notation), or a relapse which can occur at rate$f$if hypnozoites are present. Reinfection is assumed to be possible at all times (except during periods of prophylactic protection) and occurs at rate$\lambda$. Relapses may occur due to hypnozoites that persist from the previous infection.

If an infection is acquired at time $T_{\inf}^{k-1}$then we assume that hypnozoites are present at that time. Either infection *k* – 1 was a relapse in which case there are hypnozoites (we don’t assume hypnozoite depletion), or infection *k* - 1 was from a new mosquito bite which caused more hypnozoites. We can thus write down the probability that infection *k* was acquired at time$T_{\inf}^{k}$as follows:

$$\mathbf{P}_{\text{rel}}\left(T_{\inf}^{k}\right)=\mathbf{P}\left(\text{hpz at }T_{\text{clear}}^{k-1}\right)\mathbf{P}\left(\left.T_{\inf}^{k}\right|\text{hpz at }T_{\text{clear}}^{k-1}\right)+\mathbf{P}\left(\text{no hpz at }T_{\text{clear}}^{k-1}\right)\mathbf{P}\left(\left.T_{\inf}^{k}\right|\text{no hpz at }T_{\text{clear}}^{k-1}\right)$$

The components of this expression can be calculated as follows. Given that an individual was infected at time$T_{\text{inf}}^{k-1}$, the probability that they are still carrying hypnozoites when the blood-stage infection clears at time$T_{\text{clear}}^{k-1}$ is

$$\mathbf{P}\left(\text{hpz at }T_{\text{clear}}^{k-1}\right)=\frac{\lambda+\gamma_{L}e^{-\gamma_{L}d_{BS}^{k-1}}}{\lambda+\gamma_{L}}$$

where$d_{\text{BS}}^{k-1}=T_{\text{clear}}^{k-1}-T_{\text{inf}}^{k-1}$. This expression accounts for the possibility of acquiring new liver-stage hypnozoites from mosquito bites during blood-stage infection$k-1$. Note that if there was no mosquito-borne transmission (i.e.$\lambda=0$) this would simplify to$e^{-\gamma_{L}d_{BS}^{k-1}}$. And similarly, the probability that there are no hypnozoites at$T_{\text{clear}}^{k-1}$ is

$$\mathbf{P}\left(\text{no hpz at }T_{\text{clear}}^{k-1}\right)=1-\mathbf{P}\left(\text{hpz at }T_{\text{clear}}^{k-1}\right)=\frac{\gamma_{L}}{\lambda+\gamma_{L}}\left(1-e^{-\gamma_{L}d_{BS}^{k-1}}\right)$$

If there are no hypnozoites at the time of clearance of blood-stage infection, then the next infection must be due to re-infection via mosquito bite and cannot be due to a relapse. We can thus write

$$\mathbf{P}\left(\left.T_{\inf}^{k}\right|\text{no hpz at }T_{\text{clear}}^{k-1}\right)=\lambda e^{-\lambda\left(T_{\inf}^{k}-T_{\text{clear}}^{k-1}\right)}$$

Finally, we need to calculate the probability distribution of the incidence of infection when hypnozoites remain present at the time of clearance of blood-stage infection. This instance is slightly more complicated because the next infection can be due to either a new mosquito bite or a relapse. This problem can be described using the notation outlined in Figure S2.4. At time$T_{\text{clear}}^{k-1}$ a participant will have just cleared their blood-stage parasites but will still have hypnozoites, so they will be in state$S_{L}$. From this state, an individual can (i) relapse at rate$f$, $\left(S_{L}\to I_{L}\right)$; (ii) receive a new infection at rate$\lambda$,$\left(S_{L}\to I\right)$; or (iii) clear hypnozoites at rate$\gamma_{L}$ and then receive a new infection$\left(S_{L}\to S_{0}\to I\right)$. All of these transitions can be described mathematically as follows

$$\begin{matrix} \frac{dS_{L}}{dt}=-\left(\lambda+f+\gamma_{L}\right)S_{L} \\ \frac{dS_{0}}{dt}=\gamma_{L}S_{L}-\lambda S_{0} \\ \frac{dI}{dt}=\left(\lambda+f\right)S_{L}+\lambda S_{0} \\ \left\{S_{L}\left(T_{\text{clear}}^{k-1}\right)=1,S_{0}\left(T_{\text{clear}}^{k-1}\right)=0,\mathrm{I}\left(T_{\text{clear}}^{k-1}\right)=0\right\} \end{matrix}$$

The initial conditions of equation (14) indicate that following the clearance of the previous blood-stage infection, individuals are assumed to have liver-stage parasites but no blood-stage parasites (i.e. they are in state *S_L_*). This system of equations can be solved analytically to calculate the probability that an individual is infected with blood-stage parasites at time *t*:

$$I\left(t\right)=\frac{\gamma_{L}\left(1-e^{-\lambda\left(t-T_{\text{clear}}^{k-1}\right)}\right)+f\left(1-e^{-\left(f+\gamma_{L}+\lambda\right)\left(t-T_{\text{clear}}^{k-1}\right)}\right)}{f+\gamma_{L}}$$

And we can differentiate to get the incidence:

$$\mathbf{P}\left(\left.T_{\inf}^{k}\right|\text{hpz at }T_{\text{clear}}^{k-1}\right)=\left.\frac{dI(t)}{dt}\right|_{T_{\inf}^{k}}=\frac{\gamma_{L}\lambda e^{-\lambda\left(T_{\inf}^{k}-T_{\text{clear}}^{k-1}\right)}+f\left(f+\gamma_{L}+\lambda\right)e^{-\left(f+\gamma_{L}+\lambda\right)\left(T_{\inf}^{k}-T_{\text{clear}}^{k-1}\right)}}{f+\gamma_{L}}$$

Inserting equations (11), (12), (13) and (16) into equation (10) gives

$$\mathbf{P}_{\text{rel}}\left(T_{\inf}^{k}\right)=\left(\frac{\lambda+\gamma_{L}e^{-\gamma_{L}d_{BS}^{k-1}}}{\lambda+\gamma_{L}}\right)\left(\frac{\gamma_{L}\lambda e^{-\lambda\left(T_{\inf}^{k}-T_{\text{clear}}^{k-1}\right)}+f\left(f+\gamma_{L}+\lambda\right)e^{-\left(f+\gamma_{L}+\lambda\right)\left(T_{\inf}^{k}-T_{\text{clear}}^{k-1}\right)}}{f+\gamma_{L}}\right)+\left(\frac{\gamma_{L}}{\lambda+\gamma_{L}}\left(1-e^{-\gamma_{L}d_{BS}^{k-1}}\right)\right)\left(\lambda e^{-\lambda\left(T_{\inf}^{k}-T_{\text{clear}}^{k-1}\right)}\right)$$

And simplifying gives:

$$\mathbf{P}_{\text{rel}}\left(T_{\inf}^{k}\right)=\frac{\gamma_{L}\lambda\left(f+\gamma_{L}+\lambda-fe^{-\gamma_{L}d_{\text{BS}}^{k-1}}\right)e^{-\lambda\left(T_{\inf}^{k}-T_{\text{clear}}^{k-1}\right)}+f\left(f+\gamma_{L}+\lambda\right)\left(\lambda+\gamma e^{-\gamma_{L}d_{\text{BS}}^{k-1}}\right)e^{-\left(f+\gamma_{L}+\lambda\right)\left(T_{\inf}^{k}-T_{\text{clear}}^{k-1}\right)}}{\left(\lambda+\gamma_{L}\right)\left(f+\gamma_{L}\right)}$$

And including the normalising constant by integrating over the interval$\left[T_{\text{clear}}^{k-1},T_{\text{max}}\right]$gives

$$\mathbf{P}_{\text{rel}}\left(T_{\inf}^{k}\right)=\frac{\gamma_{L}\lambda\left(f+\gamma_{L}+\lambda-fe^{-\gamma_{L}d_{\text{BS}}^{k-1}}\right)e^{-\lambda\left(T_{\inf}^{k}-T_{\text{clear}}^{k-1}\right)}+f\left(f+\gamma_{L}+\lambda\right)\left(\lambda+\gamma e^{-\gamma_{L}d_{\text{BS}}^{k-1}}\right)e^{-\left(f+\gamma_{L}+\lambda\right)\left(T_{\inf}^{k}-T_{\text{clear}}^{k-1}\right)}}{\gamma_{L}\left(f+\gamma_{L}+\lambda-fe^{-\gamma_{L}d_{\text{BS}}^{k-1}}\right)\left(1-e^{-\lambda\left(T_{\max}-T_{\text{clear}}^{k-1}\right)}\right)+f\left(\lambda+\gamma e^{-\gamma_{L}d_{\text{BS}}^{k-1}}\right)\left(1-e^{-\left(f+\gamma_{L}+\lambda\right)\left(T_{\max}-T_{\text{clear}}^{k-1}\right)}\right)}$$

Equation (19) depicts the probability distribution for the time to next infection accounting for both new infections and relapses. Figure S2.5 shows a comparison of the probability distribution for new infections plus relapse (equation (19)) with new infection only (equation (6)).


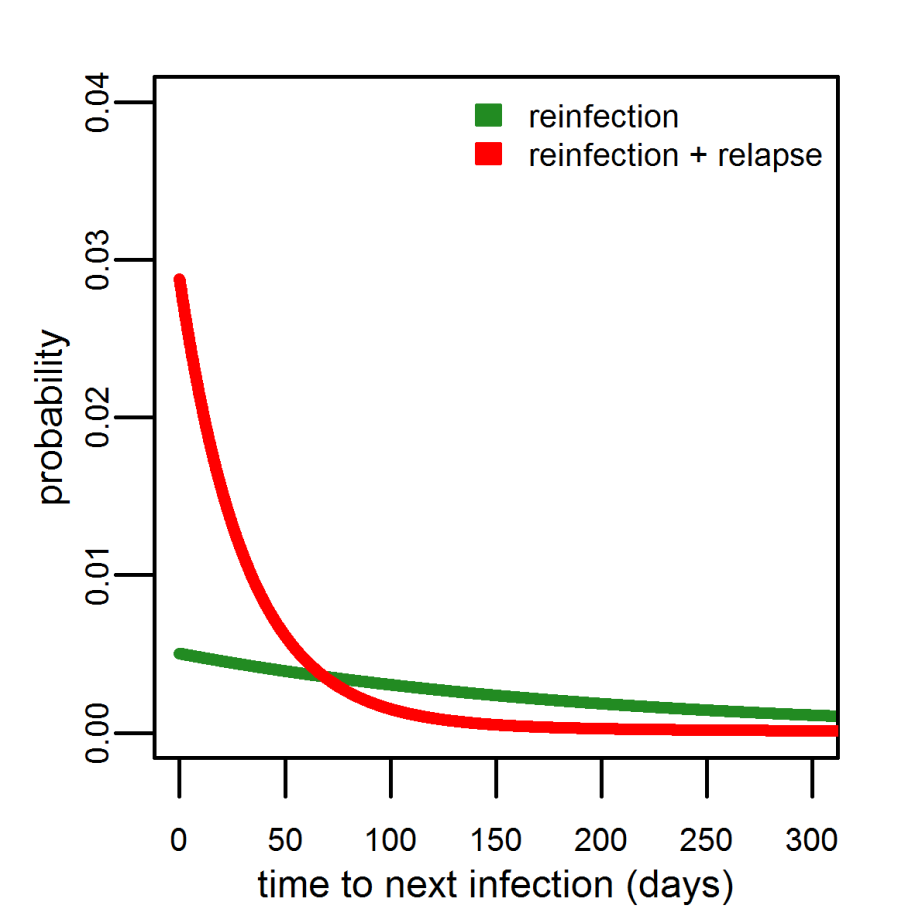


Figure S2.5: Probability distribution for time to next infection $\mathbf{P}\left(T_{\inf}^{k}\right)$ for a model with and without relapse.

**2.5. *P. vivax* infection dynamics without hypnozoite clearance at start of follow-up**

**Accounting for infection status before treatment**

In a treatment re-infection study, the presence or absence of *Plasmodium* parasites before treatment does not affect the *P. falciparum* infection dynamics during the subsequent period of longitudinal follow-up, as all participants will be in the same state once the effects of treatment prophylaxis have waned, namely, everyone is free of *P. falciparum* blood-stage parasites. Note that we do not account for the potential role of heterogeneity in exposure to mosquito bites. It is possible that the presence of *Plasmodium* parasites before treatment is indicative of higher exposure, and an increased probability of acquiring new genotypes during the period of longitudinal follow up. Similarly for *P. vivax*, if the treatment re-infection study includes primaquine such that all parasites (both liver-stage and blood-stage) are cleared at the beginning of the study, then the presence or absence of *P. vivax* blood-stage parasites before treatment will not affect the *P. vivax* infection dynamics during longitudinal follow up.

If the treatment regimen in a treatment re-infection study does not include primaquine, then the presence or absence of *P. vivax* blood-stage parasites before treatment may affect the *P. vivax* infection dynamics once prophylaxis has waned. If blood-stage *P. vivax* parasites are detected before treatment is administered, then there is a higher probability that hypnozoites are also present leading to relapses. In contrast, if no *P. vivax* blood-stage parasites are detected before treatment, there is a lower probability that hypnozoites are present in the liver.

The probability of hypnozoite infection given the presence or absence of blood-stage *P. vivax* parasites can be calculated by finding the equilibrium solution of the system of differential equations corresponding to the model in Figure S2.4.

$$\begin{matrix} \frac{dS_{0}}{dt}=-\lambda S_{0}+rI_{0}+\gamma_{L}S_{L} \\ \frac{dI_{0}}{dt}=-\lambda I_{0}-rI_{0}+\gamma_{L}I_{L} \\ \frac{dS_{L}}{dt}=-\lambda S_{L}+rI_{L}-fS_{L}-\gamma_{L}S_{L} \\ \frac{dI_{L}}{dt}=\lambda\left(S_{0}+I_{0}+S_{L}\right)-rI_{L}+fS_{L}-\gamma_{L}I_{L} \end{matrix}$$

It can be shown that subject to a constant force of infection of $\lambda$from mosquitoes, that the system has the following equilibrium solution:

$$\begin{matrix} S_{0}^{*}=\frac{r\gamma_{L}\left(f+r+\gamma_{L}+2\lambda\right)}{\left(r+\lambda\right)\left(\gamma_{L}+\lambda\right)\left(f+r+\gamma_{L}+\lambda\right)} \\ I_{0}^{*}=\frac{\gamma_{L}\lambda\left(f+\gamma_{L}+\lambda\right)}{\left(r+\lambda\right)\left(\gamma_{L}+\lambda\right)\left(f+r+\gamma_{L}+\lambda\right)} \\ S_{L}^{*}=\frac{r\lambda\left(r+\lambda\right)}{\left(r+\lambda\right)\left(\gamma_{L}+\lambda\right)\left(f+r+\gamma_{L}+\lambda\right)} \\ I_{L}^{*}=\frac{\lambda\left(r+\lambda\right)\left(f+\gamma_{L}+\lambda\right)}{\left(r+\lambda\right)\left(\gamma_{L}+\lambda\right)\left(f+r+\gamma_{L}+\lambda\right)} \end{matrix}$$

The solution above depends on a number of assumptions. A child will begin life with no blood or liver-stage parasites. It is assumed that force of infection is constant with age and that participants in the trial are old enough so that equilibrium has been reached. This solution also assumes that the duration of blood-stage infection is exponentially distributed with mean 1/*r*. Alternative distributions for the duration of blood-stage infection may affect the equilibrium derived above, but the difference is likely to be negligible.

We use the equilibrium expressions in equation (21) to estimate the probability of hypnozoites being present at the beginning of follow-up, depending on whether that individual had detectable blood-stage parasites. First we calculate the probability that given a negative measurement of blood-stage parasites before follow-up (i.e.$x^{0}=0$at $\tau^{0}$) hypnozoites are present.

$$\begin{matrix} \mathbf{P}\left(\text{hpz at }\tau^{0}\left|x^{0}=0\right.\right)=\frac{\mathbf{P}\left(\text{hpz at }\tau^{0}\cap x^{0}=0\right)}{\mathbf{P}\left(x^{0}=0\right)} \\ =\frac{S_{L}^{*}+\left(1-q\right)I_{L}^{*}}{S_{0}^{*}+\left(1-q\right)I_{0}^{*}+S_{L}^{*}+\left(1-q\right)I_{L}^{*}} \\ =\frac{\lambda\left(r+\lambda\right)\left(r+\left(1-q\right)\left(f+\gamma_{L}+\lambda\right)\right)}{\left(\gamma_{L}+\lambda\right)\left(r+\gamma_{L}+\lambda\right)\left(r+\lambda\left(1-q\right)\right)+f\left(\lambda\left(\gamma_{L}+\lambda\right)\left(1-q\right)+r\left(\gamma_{L}+\lambda\left(1-q\right)\right)\right)} \end{matrix}$$

Next we calculate the probability that hypnozoites are present at$\tau^{0}$given that blood-stage parasites were detected at that time$\left(x^{0}=1\right)$.

$$\begin{matrix} \mathbf{P}\left(\text{hpz at }\tau^{0}\left|x^{0}=1\right.\right)=\frac{\mathbf{P}\left(\text{hpz at }\tau^{0}\cap x^{0}=1\right)}{\mathbf{P}\left(x^{0}=1\right)} \\ =\frac{qI_{L}^{*}}{qI_{0}^{*}+qI_{L}^{*}} \\ =\frac{r+\lambda}{\gamma_{L}+r+\lambda} \end{matrix}$$

Given the presence of detected *P. vivax* parasites at enrolment$y=1$, or the absence of *P. vivax* parasites$y=0$, the expression for the likelihood of the time to infection is given by:

$$\mathbf{P}\left(T_{\inf}^{1}\left|x^{0}=y\right.\right)=\mathbf{P}\left(\text{hpz at }\tau^{0}\left|x^{0}=y\right.\right)\mathbf{P}_{\text{rel}}\left(T_{\inf}^{1}\right)+\left(1-\mathbf{P}\left(\text{hpz at }\tau^{0}\left|x^{0}=y\right.\right)\right)\mathbf{P}\left(T_{\inf}^{1}\right)$$

As both $\mathbf{P}\left(T_{\inf}^{1}\right)$ and$\mathbf{P}_{\text{rel}}\left(T_{\inf}^{1}\right)$ are normalised, equation (24) is also normalised.

**2.6. Application to data from longitudinal cohort studies without a treatment re-infection design**

The methods described above are applicable for samples collected in a longitudinal study with a teatment re-infection design where participants begin the study by being cleared of blood-stage parasites (and cleared of *P. vivax* liver-stage parasites in the case when primaquine is administered). The data from Thailand comes from a longitudinal observational study: samples taken once a month for 14 months without the administration of treatment. As such, the infection status of participants at the start of the study is unkown. If parasites are detected, we don’t know when those parasites are acquired. If parasites are not detected, we can’t rule out that parasites were present but were not detected. To account for this in the Thai cohort, we simulated infections in Thai participants for a year before the first sample was collected. In particular, we assumed that infections could occur from$T_{\text{start}}=\tau^{0}-365$ and that no blood-stage parasites were present at this time. During the period between *T_start_* and *τ_0_* it is possible that infections may be acquired and cleared.

For the case of *P. vivax*, we don’t know whether hypnozoites were present at$T_{\text{start}}$ , but we can follow a similar methodology as above to say:

$$\begin{matrix} \mathbf{P}\left(\text{hpz at }T_{\text{start}}\right)=S_{L}^{*}+I_{L}^{*} \\ =\frac{\lambda}{\lambda+\gamma_{L}} \end{matrix}$$

**2.7. Estimation of the proportion of *P. vivax* infections due to relapse**

Figure 7 of the main manuscript shows a comparison of the proportion of new *P. vivax* infections due to relapses and the proportion of total *P. vivax* infections due to relapses. Here we show how these proportions were estimated.

We first show how the probability that individual *n* was infected with genotype *g* at time *t* (this is applicable for both *P. falciparum* and *P. vivax*). Assume that we have *M* samples from the estimated posterior distributions of the data augmented parameters for the times of acquisition and clearance of *k*^th^ infection. We can estimate the probability that infection *k* was present at time *t* by averaging over the posterior as follows (the superscripts *g* and *n* have been dropped for convenience):

$$\mathbf{Z}^{k}\left(t\right)=\frac{1}{M}\sum_{m} \mathbf{1}\left(T_{\inf}^{k}\left(m\right)<t<T_{\text{clear}}^{k}\left(m\right)\right)$$

And we can estimate the probability that an individual was infected with any of *K* infections by summing as follows

$$\mathbf{Z}\left(t\right)=\sum_{k}^{K} \frac{1}{M}\sum_{m} \mathbf{1}\left(T_{\inf}^{k}\left(m\right)<t<T_{\text{clear}}^{k}\left(m\right)\right)$$

We can now estimate the probability that when infection *k* was acquired at time $T_{\inf}^{k}$, that it was due to a relapse as follows.

$$\text{R}_{\text{new}}^{k}\left(t\right)=\frac{1}{M}\sum_{m} \mathbf{1}\left(T_{\inf}^{k}\left(m\right)<t<T_{\text{clear}}^{k}\left(m\right)\right)\frac{f\left(m\right)}{\lambda\left(m\right)+f\left(m\right)}\frac{\lambda\left(m\right)+\gamma_{L}\left(m\right)e^{-\gamma_{L}\left(m\right)d_{\text{BS}}^{k-1}\left(m\right)}}{\lambda\left(m\right)+\gamma_{L}\left(m\right)}e^{-\gamma_{L}\left(m\right)\left(T_{\text{inf}}^{k}\left(m\right)-T_{\text{clear}}^{k-1}\left(m\right)\right)}$$

And averaging over *K* infections gives:

$$\mathbf{R}_{\text{new}}\left(t\right)=\sum_{k}^{K} \mathbf{R}_{\text{new}}^{k}\left(t\right)$$

Equation (29) provides the probability that *P. vivax* parasites at time *t* first originated from a relapse. This accounts for the fact that a first infection is more likely to be a re-infection from a mosquito bite than subsequent infections. Notably, during the period of blood-stage parasitaemia arising from the primary infection, there are likely to be subsequent relapses of the same genotype that go undetected. The following formula allows us to compare the relative incidence of re-infection (rate $\lambda$) and relapse (rate$f\left(S_{L}\left(t\right)+I_{L}\left(t\right)\right)$). This provides an expression for the proportion of total infections due to relapses:

$$\mathbf{R}_{\text{total}}\left(t\right)=\frac{f\left(S_{L}\left(t\right)+I_{L}\left(t\right)\right)}{\lambda+f\left(S_{L}\left(t\right)+I_{L}\left(t\right)\right)}$$

If we assume that individuals begin with no blood-stage or liver-stage parasites then expressions for$S_{L}\left(t\right)$ and$I_{L}\left(t\right)$ can be derived via equation (20) to provide the following:

$$\mathbf{R}_{\text{total}}\left(t\right)=\frac{f\left(1-e^{-\left(\lambda+\gamma_{L}\right)t}\right)}{\lambda+\gamma_{L}+f\left(1-e^{-\left(\lambda+\gamma_{L}\right)t}\right)}$$

Notable this equation has an equilibrium value of$\frac{f}{\lambda+\gamma_{L}+f}$ which gives the steady state proportion of total infections due to relapse.

The probability that a new infection is attributable to a relapse as shown in equation (29) is based on the estimated posterior distributions. Figure 6 plots the association between these estimate relapse probabilities and epidemiological covariates such as the time of fevers and treatments.

**2.8. MCMC estimation procedure**

The data and model can be organised in a structured, hierarchical manner with statistical inference undertaken in a mixed effects framework. Figure S2.6 shows a schematic representation of how the model is structured across three levels.


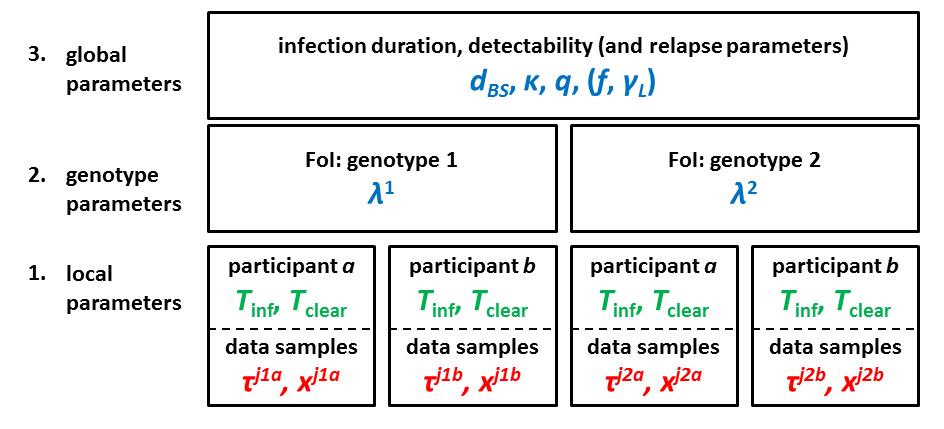


Figure S2.6: Schematic overview of the model structure and data. For each genotype *g* within each individual *n* there will be data samples *x^jgn^* at times τ^jgn^. This data (shown in red) will be described by local data-augmented parameters *T*_inf_ and *T*_clear_ representing the time of acquisition and clearance of k infections (shown in green). At the next level up the genotype specific parameters for the force of infection λ­^g^ will depend on data samples of genotype *g* from all participants. Finally the global parameters for infection duration, detectability and relapse characteristics (for the case of *P. vivax*) will depend on all the data.

The model was fitted to the data using Markov Chain Monte Carlo (MCMC) methods. Parameters were updated at each MCMC iteration using a random walk Metropolis-Hastings algorithm with three update stages illustrated below. A ′ indicates an attempted update.

1. **Individual-level parameter update.**Parameters are updated for each participant$n$ and each genotype$g$ .
   The sample data for genotype$g$in participant$n$is denoted$D_{\text{ind}}^{gn}=\left\{x^{jgn},\tau^{jgn}\left|1\leq j\leq J^{gn}\right.\right\}$. The data augmented parameters for the times of acquisition and clearance of blood-stage infection also exist on an individual level and are denoted$\theta_{\text{ind}}^{gn}=\left\{T_{\inf}^{kgn},T_{\text{clear}}^{kgn}\left|1\leq k\leq K^{gn}\right.\right\}$.

- Propose from a multivariate Normal distribution
- Account for censoring due to treatment
- Calculate updated mixed effects likelihood
- Accept the parameter update with probability

1. **Genotype-level parameter update.**Parameters are updated for all individuals and each genotype$g$. The data are denoted as follows$D^{g}=\left\{D^{gn}\left|1\leq n\leq N\right.\right\}$. There is only a single parameter for each genotype, namely the force of infection$\theta^{g}=\left\{\lambda^{g}\right\}$.
   - Propose from a Normal distribution
   - Calculate updated likelihood
   - Accept the parameter update with probability
2. **Population-level parameter update**Population-level parameters affect the likelihood from all participants and all genotypes. The data are denoted as follows$D=\left\{D^{g}\left|1\leq g\leq G\right.\right\}$. The parameters are denoted as$\theta_{\text{pop}}=\left\{d_{\text{BS}},\kappa,q\right\}$ for *P. falciparum* and $\theta_{\text{pop}}=\left\{d_{\text{BS}},\kappa,q,f,\gamma_{L}\right\}$ for *P. vivax*.

- Propose from a multivariate Normal distribution$\theta_{\text{pop}}^{'}\sim MVN\left(\theta_{\text{pop}},\Sigma_{\text{pop}}\right)$
  - Calculate updated likelihood$L\left(\theta_{\text{pop}}^{'}\left|D\right.\right)$
  - Accept the parameter update with probability $\min\left(1,\frac{L\left(\theta_{\text{pop}}^{'}\left|D\right.\right)P\left(\theta_{\text{pop}}^{'}\right)}{L\left(\theta_{\text{pop}}\left|D\right.\right)P\left(\theta_{\text{pop}}\right)}\right)$

**Computational implementation of MCMC fitting procedure**

The hierarchical structure of the data and model depicted in Figure S2.6 aids in the generation of converged and well mixing MCMC chains. This allows for the data augmented parameters on the individual-level to be tuned first, followed by the genotype-level parameters followed by population-level parameters. The proposal distribution for the Metropolis-Hastings step is adaptively tuned via two mechanisms. Firstly, a Robbins-Munro algorithm is used to tune the magnitude of the proposed step, so that the MCMC acceptance rate is approximately 23%. Secondly, the covariance matrix of the multivariate Normal distribution is adaptively updated using samples from the estimated posterior distribution.

In practice, the tuning of the system is implemented as follows:

1. **Individual-level parameter tuning (1,000,000 iterations)**For each genotype and each individual the data augmented parameters for the times of acquisition and clearance of infection$\theta_{\text{ind}}^{gn}=\left\{T_{\inf}^{kgn},T_{\text{clear}}^{kgn}\left|1\leq k\leq K^{gn}\right.\right\}$are updated in parallel. The genotype-level and population-level parameters are kept fixed. For each genotype in each individual, the covariance matrix of the multivariate Normal proposal distribution $\left(\Sigma_{\text{ind}}^{gn}\right)$ is tuned.
2. **Genotype-level parameter tuning (1,000,000 iterations)**For each genotype *g*, the genotype-specific force of infection $\lambda^{g}$ is tuned. The proposal distributions for the individual-level data augmented parameters are fixed according to the previous step. Individual-level parameters are updated but population-level parameters are kept fixed. As there is only one genotype-level parameter per genotype, only the standard deviation of the Normal proposal distribution $\left(\sigma^{g}\right)$ needs to be tuned. This is done using the Robbins-Munro algorithm.

1. **Population-level parameter tuning (1,000,000 iterations)**The individual-level and genotype-level proposal distributions are fixed according to the previous step. Individual-level, genotype-level and population-level parameters are all updated simultaneously. The covariance matrix of the multivariate Normal proposal distribution $\left(\Sigma_{\text{pop}}\right)$ is tuned.
2. **Statistical inference (1,000,000 + 20,000,000 iterations)**Once the proposal distributions for each of the 3 levels have been tuned through the previous steps, they are fixed. Parameters across each of the 3 levels are simultaneously updated for 1,000,000 iterations to allow for burn-in. The MCMC algorithm is then run for another 20,000,000 iterations, which are used to provide samples from the estimated posterior distributions.

The 20,000,000 iterations used for statistical inference were trimmed to provide 10,000 samples from the posterior. Due to the large memory requirements to store the output of the posterior for each of the (504 or 999)*14 individuals, the individual-level parameters were trimmed to provide 1,000 samples. For each model applied to data from each cohort, the MCMC procedure was implemented twice. Chains were examined for convergence using the Gelman-Rubin statistic$R_{c}$[[4](#_ENREF_4)]. The chains corresponding to the population-level and genotype-level parameters were checked to ensure$R_{c}<1.1$. The chains corresponding to the individual-level parameters were checked to ensure$R_{c}<1.2$.

We had data from 14 genotypes in 504 participants from the Papua New Guinean cohort and 999 participants in the Thai cohort. For participant *n* with at least one positive sample of genotype *g*, we sampled *K^gn^* = 3 infections. A large proportion of individuals did not have any positive samples. For participant *n* with no positive samples of genotype *g*, we only sampled *K^gn^* = 1 infection for computational efficiency. This allows for the possibility that even in individuals with no positive samples, infection may have occurred but gone undetected.

**References**

1. Luxemburger C, van Vugt M, Jonathan S, McGready R, Looareesuwan S, et al. (1999) Treatment of vivax malaria on the western border of Thailand. Transactions of the Royal Society of Tropical Medicine and Hygiene 93: 433-438.

2. Bretscher MT, Maire N, Chitnis N, Felger I, Owusu-Agyei S, et al. (2011) The distribution of Plasmodium falciparum infection durations. Epidemics 3: 109-118.

3. White MT, Shirreff G, Karl S, Ghani AC, Mueller I (2016) Variation in relapse frequency and the transmission potential of Plasmodium vivax malaria. Proc Roy Soc B.

4. Brooks SP, Gelman A (1998) General methods for monitoring convergence of iterative simulations. Journal of Computational and Graphical Statistics 7: 434-455.
