# Supplementary material for: Plasmodium vivax and Plasmodium falciparum infection dynamics: re-infections, recrudescences and relapses
Source: Malar J. 2018 Apr 17;17:170. doi: 10.1186/s12936-018-2318-1 (PMC5905131; doi:10.1186/s12936-018-2318-1)
Supplement: Supplementary file 3 — Additional file 3. Model validation on simulated data. [file 12936_2018_2318_MOESM3_ESM.docx]

***Plasmodium vivax* and *Plasmodium falciparum* infection dynamics: re-infections, recrudescences and relapses**

*Michael White, Stephan Karl, Cristian Koepfli, Rhea Longley, Natalie E. Hofmann, Rahel Wampfler, Ingrid Felger, Tom Smith, Wang Nguitragool, Jetsumon Sattabongkot, Leanne Robinson, Azra Ghani, Ivo Mueller*

**Additional file 3: Model validation on simulated data**

The limitations of the mathematical models for *P. falciparum* and *P. vivax* infection dynamics and the accompanying methods for statistical inference were investigated by applying them to simulated data sets with known values of global parameters. Fixed values for the global parameters describing the epidemiology of *P. falciparum* and *P. vivax* are used to create cohorts of individuals with simulated infection histories for the presence or absence of each genotype. The method for statistical inference is then applied to test whether the original global parameters can be estimated from the individual-level data.

A key assumption of the methods described here is that there is homogeneous exposure to infectious mosquito bites. In reality, exposure to mosquito bites is likely to be heterogeneous between individuals and over time. For each set of global parameters, two data sets were simulated assuming either homogeneous or heterogeneous exposure. The same method was then applied to both data sets to test whether statistical inference is possible in the presence of heterogeneity.

**3.1. Testing the *P. falciparum* model on simulated data**

Hypothetical cohorts of individuals were simulated to investigate the ability of the method to accurately estimate the parameters describing the population-level epidemiology of blood-stage *P. falciparum* infection. The cohorts were assumed to have similar characteristics to the Papua New Guinean cohort, with each individual treated at the beginning of follow-up to clear any existing blood-stage infections. Following a month of prophylactic protection, the stochastic acquisition and clearance of blood-stage *P. falciparum* infections were simulated for 8 months. We assumed samples were taken every 2 weeks for the first 3 months, and every month for the next 5 months. We assumed a cohort of 500 individuals, with testing for 3 different single locus *P. falciparum* genotypes.

We simulated data sets across the following range of parameter values: $d_{BS}\in\left\{20,30,50,100,150,200\right\}$ days, and$q\in\left\{30\%,50\%,70\%\right\}$. We included scenarios where the duration of blood-stage infections followed an exponential distribution, or where they followed a Weibull distribution with shape parameter$k_{WB}\in\left\{0.5,1,2\right\}$. We assumed genotype-specific forces of infection, whereby the three genotype are expected to infect 40%, 10% and 5% of individuals in a year. For each parameter set, three data sets were simulated: (i) assuming a homogeneous force of infection; and (ii) assuming heterogeneity in exposure to mosquito bites described by a log-Normal distribution with standard deviation on a log-scale$\sigma^{2}=1.67$; and (iii) assuming seasonality in expsoure to mosquito bites with the force of infection of genotype *g* varying seasonally according to $\lambda^{g}\left(t\right)=\lambda^{g}\left(1+0.75\sin\left(\frac{2\pi t}{365}\right)\right)$ .

The results of estimating the parameters describing the epidemiology of *P. falciparum* infections from simulated data assuming exponentially distributed duration of blood-stage infeections are presented in Figure S3.1. In general the method accurately estimates the parameter values for data generated according to both homogeneous and heterogeneous infection processes. However, there are some regions of parameter space where the global parameters were not reliably estimated:

- Long durations of blood-stage infection (*d_BS_* > 100 days) are underestimated. This is attributable to the long duration of infection in comparison to the duration of longitudinal sampling (~ 8 months). This was tested by simulating data sets with longer duration of follow-up (~15 months) where longer values of infection duration can be more reliably estimated (simulations not shown).
- Combinations of short infections with high sensitivity of genotype detection are not consistently identifiable. This is due to a lack of identifiablity between a short infection, high detectability scenario and a high force of infection, low detectability scenario. For example, a 01010 pattern can be explained by two short infections (requiring a higher FoI) or by a single longer infection (requiring lower sensitivity).


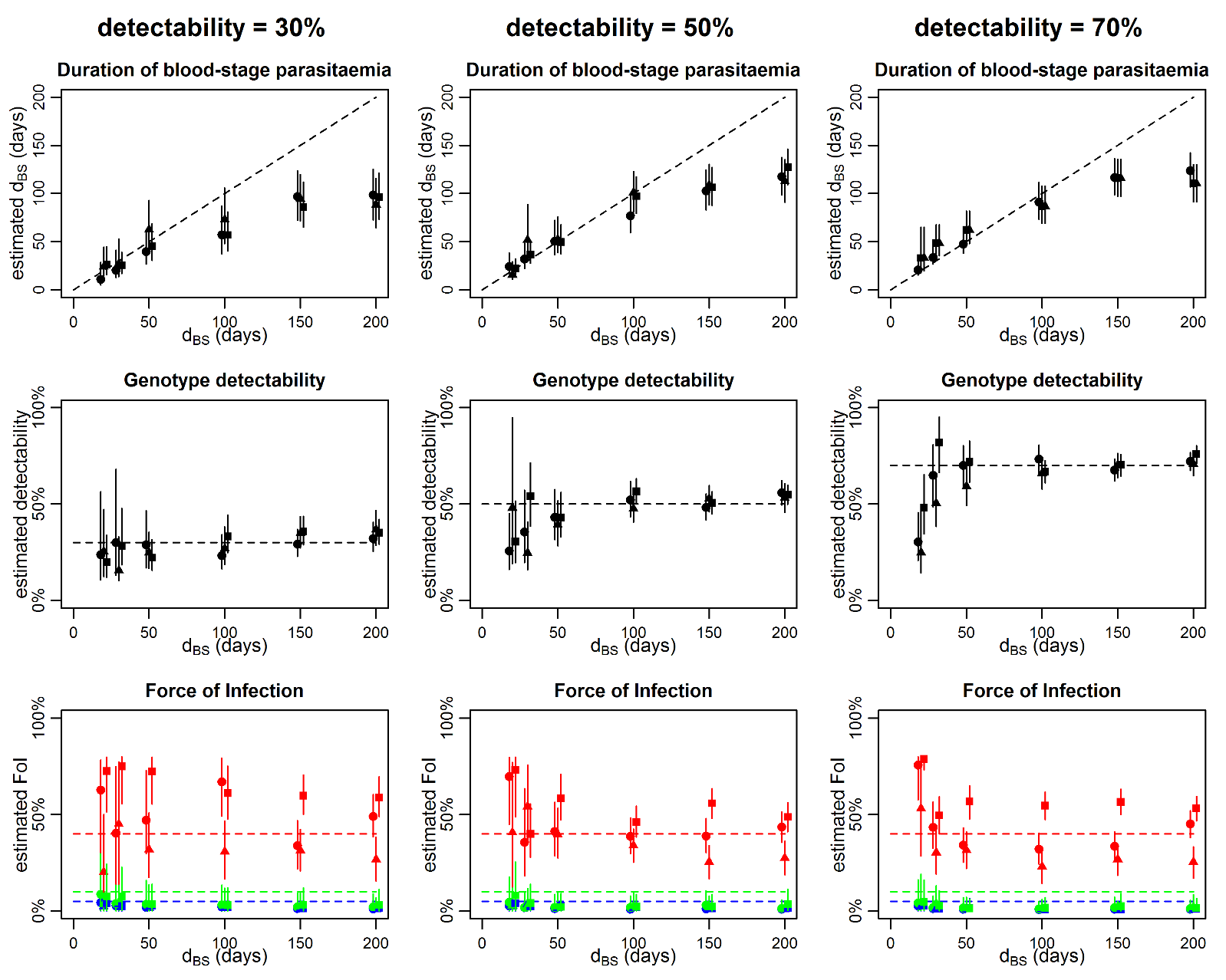


Figure S3.1: Validation of the *P. falciparum* model with exponentially distributed duration of blood-stage infection using simulated data. The points and vertical bars represent the median and 95% credible intervals of the estimated posterior distributions. For each combination of parameters, the model was fitted to simulated data assuming homogeneous (circles), heterogeneous (triangles), or seasonal (squares) exposure to infectious mosquito bites. Each column presents the results of simulations for a different value of genotype sensitivity. The dashed lines denote the values of the global parameters used for simulating the data.

The results of estimating the parameters describing the epidemiology of *P. falciparum* infections from simulated data assuming Weibull distributed duration of blood-stage infections are presented in Figure S3.2 (*k_WB_* = 0.5), Figure S3.3 (*k_WB_* = 1) and Figure S3.4 (*k_WB_* = 2). In general the method accurately estimates the parameter values for data generated according to both homogeneous and heterogeneous infection processes. There are some regions of parameter space where global parameters were not reliably estimated.

- Smaller values of the Weibull shape parameter (*k_WB_* ≤ 1) correspond to a greater degree of variation in duration of blood-stage infection between individuals, with many short infections and a smaller number of long infections. Parameters such as the average duration of blood-stage infection are more accurately estimated when there is less variation (e.g. when *k_WB_* = 2). This finding agrees with similar results presented by Bretscher *et al* using a different methodology. For smaller values of the Weibull shape parameter (e.g. *k_WB_* = 0.5), the increased variation makes the average duration of blood-stage infection more difficult to estimate, and we substantially under-estimate the duration of blood-stage infections for larger values (*d_BS_* ≥ 100 days).
- The force of infection tends to be over-estimated for shorter values of the duration of blood-stage infection.


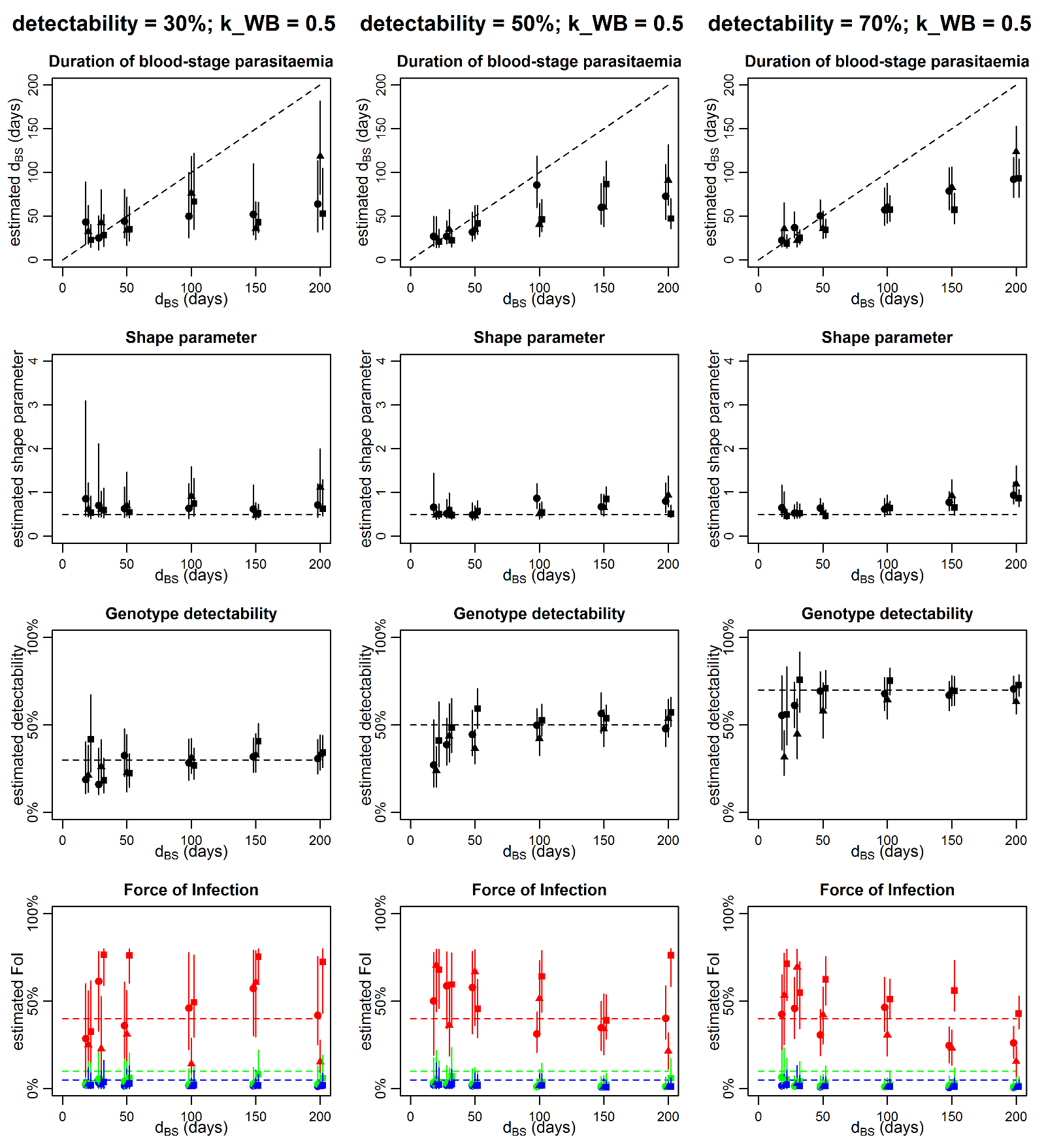


Figure S3.2: Validation of the *P. falciparum* model with Weibull distributed duration of blood-stage infection using simulated data with a shape parameter *k_WB_* = 0.5. The points and vertical bars represent the median and 95% credible intervals of the estimated posterior distributions. For each combination of parameters, the model was fitted to simulated data assuming homogeneous (circles), heterogeneous (triangles) or seasonal (squares) exposure to infectious mosquito bites. Each column presents the results of simulations for a different value of genotype sensitivity. The dashed lines denote the values of the global parameters used for simulating the data.


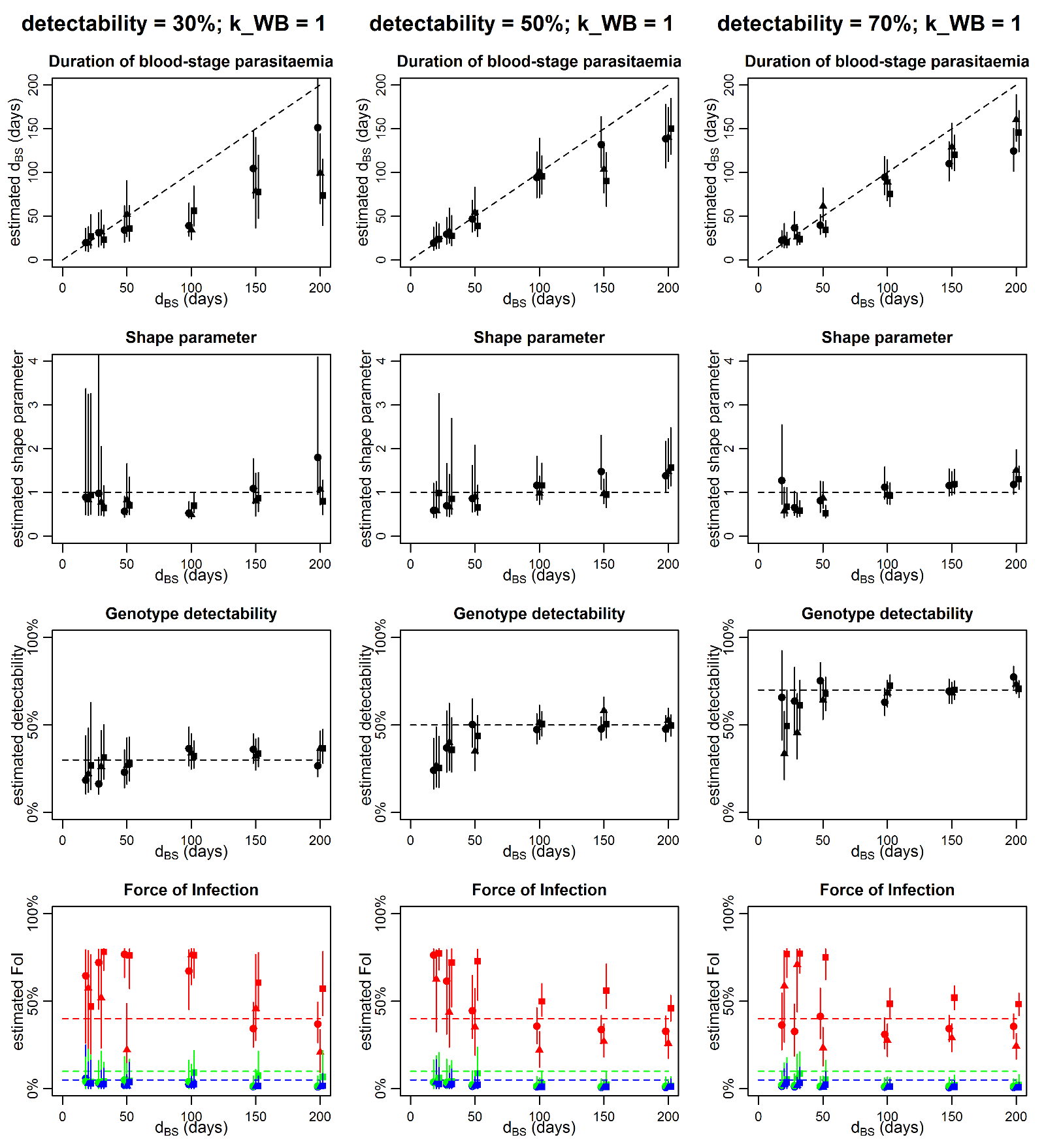


Figure S3.3: Validation of the *P. falciparum* model with Weibull distributed duration of blood-stage infection using simulated data with a shape parameter *k_WB_* = 1. The points and vertical bars represent the median and 95% credible intervals of the estimated posterior distributions. For each combination of parameters, the model was fitted to simulated data assuming homogeneous (circles), heterogeneous (triangles) or seasonal (squares) exposure to infectious mosquito bites. Each column presents the results of simulations for a different value of genotype sensitivity. The dashed lines denote the values of the global parameters used for simulating the data.


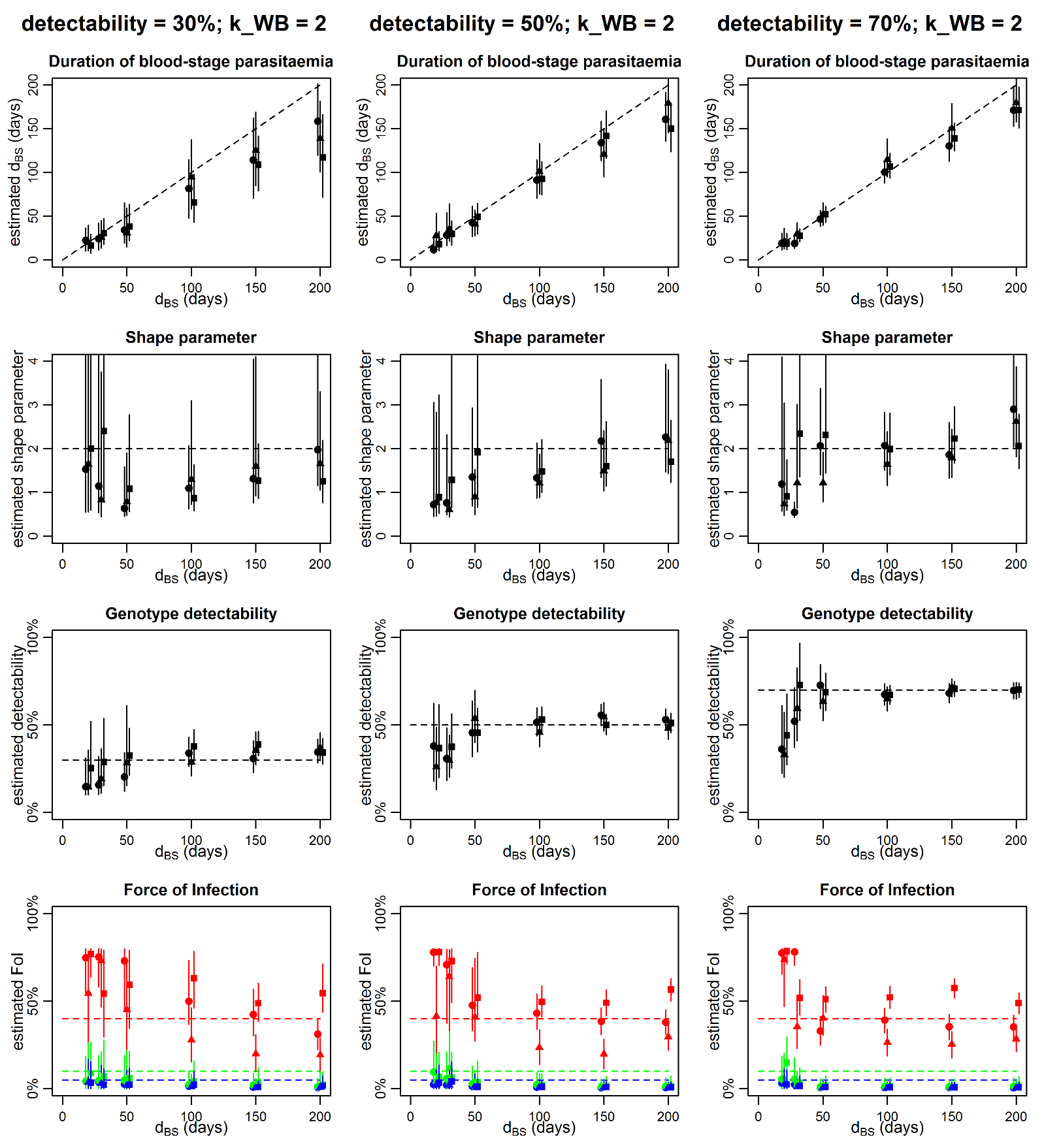


Figure S3.4: Validation of the *P. falciparum* model with Weibull distributed duration of blood-stage infection using simulated data with a shape parameter *k_WB_* = 2. The points and vertical bars represent the median and 95% credible intervals of the estimated posterior distributions. For each combination of parameters, the model was fitted to simulated data assuming homogeneous (circles), heterogeneous (triangles) or seasonal (squares) exposure to infectious mosquito bites. Each column presents the results of simulations for a different value of genotype sensitivity. The dashed lines denote the values of the global parameters used for simulating the data.

**3.2. Testing the *P. vivax* model on simulated data**

Hypothetical cohorts of individuals were simulated to investigate the ability of the method to accurately estimate the parameters describing the population-level epidemiology of blood-stage *P. vivax* infection. As for *P. falciparum*, the cohorts were assumed to have similar characteristics to the Papua New Guinean cohort, with each individual treated at the beginning of follow-up to clear any existing blood-stage infections. However, half of the cohort (250) was assumed to also be effectively treated with primaquine so that all liver-stage hypnozoites were cleared. Following a month of prophylactic protection, the stochastic acquisition (due to new infections or relapses) and clearance of blood-stage *P. vivax* infections were simulated for 8 months. We assumed samples were taken every 2 weeks for the first 3 months, and every month for the next 5 months. We assumed a cohort of 500 individuals, with testing for 3 different *P. vivax* genotypes.

We simulated data sets across the following range of parameter values: $d_{BS}\in\left\{20,30,50,100,150,200\right\}$ days, and$q\in\left\{30\%,50\%,70\%\right\}$. We included scenarios where the duration of blood-stage infections followed an exponential distribution, or where they followed a Weibull distribution with shape parameter$k_{WB}\in\left\{0.5,1,2\right\}$. *P. vivax* relapses were assumed to have a constant rate of relapse of *f* = 1/50 days^-1^, and a rate of clearance of liver-stage infection of γ_L_ = 1/300 days^-1^. We assumed genotype-specific forces of infection, whereby each genotype is expected to infect 40%, 10% and 5% of individuals in a year. For each parameter set, three data sets were simulated: (i) assuming a homogeneous force of infection; and (ii) assuming heterogeneity in exposure to mosquito bites described by a log-Normal distribution with standard deviation on a log-scale$\sigma^{2}=1.67$; and (iii) assuming seasonality in expsoure to mosquito bites with the force of infection of genotype *g* varying seasonally according to $\lambda^{g}\left(t\right)=\lambda^{g}\left(1+0.75\sin\left(\frac{2\pi t}{365}\right)\right)$ .

The results of estimating the parameters describing the epidemiology of *P. vivax* infections from simulated data assuming exponentially distributed duration of blood-stage infeections are presented in Figure S3.5. Whilst some parameters are accurately estimated, there is a very notable tendency for the duration of blood-stage infections to be under-estimated. This stems from a fundamental problem of lack of identifiability between competing processes that may explain observed blood-stage *P. vivax* infections. For example, a 01110 pattern with 3 samples positive for a given *P. vivax* genotype over a period of about 60 days can be explained by both of the following:

1. A single blood-stage infection lasting about 60 days.
2. Three separate blood-stage infections each lasting about 20 days. The existence of rapidly occuring relapses ensures that the second and third infections occur almost immediately after clearance of the preceding infection.

We therefore conclude that the method cannot reliably estimate the duration of blood-stage *P. vivax* infection because of a lack of identifiability between a blood-stage infection of long duration following a mosquito bite or a blood-stage infection of short duration rapidly followed by relapses.

**
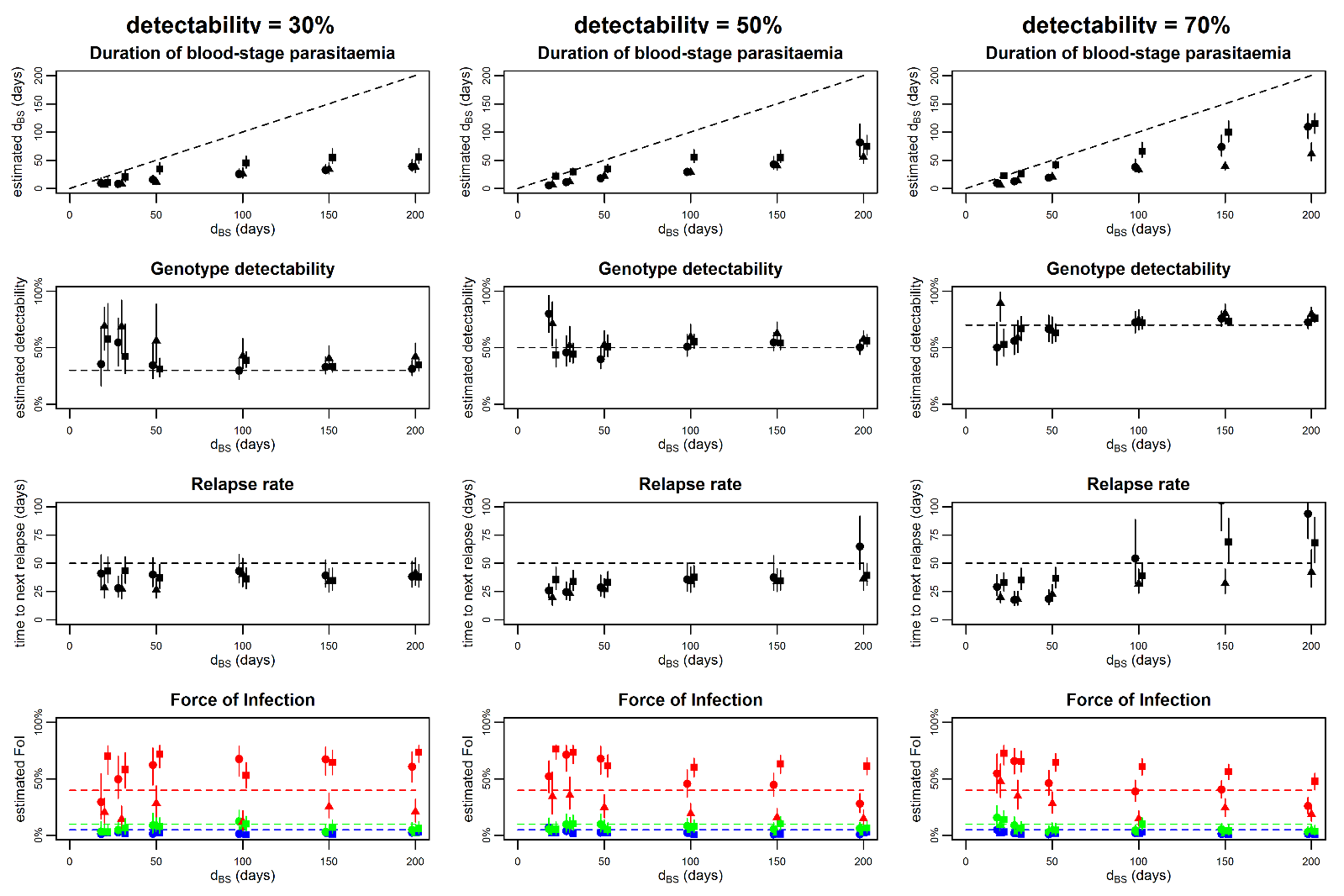
**

Figure S3.5: Validation of the *P. vivax* model with exponentially distributed duration of blood-stage infection using simulated data. The points and vertical bars represent the median and 95% credible intervals of the estimated posterior distributions. For each combination of parameters, the model was fitted to simulated data assuming homogeneous (circles), heterogeneous (triangles) or seasonal (squares) exposure to infectious mosquito bites. Each column presents the results of simulations for a different value of genotype sensitivity. The dashed lines denote the values of the global parameters used for simulating the data.

The results of estimating the parameters describing the epidemiology of *P. vivax* infections from simulated data assuming Weibull distributed duration of blood-stage infections are presented in Figure S3.6 (*k_WB_* = 0.5), Figure S3.7 (*k_WB_* = 1) and Figure S3.8 (*k_WB_* = 2). For the same reasons as discussed above, the method performs quite poorly at estimating the duration of *P. vivax* blood-stage infections.

**
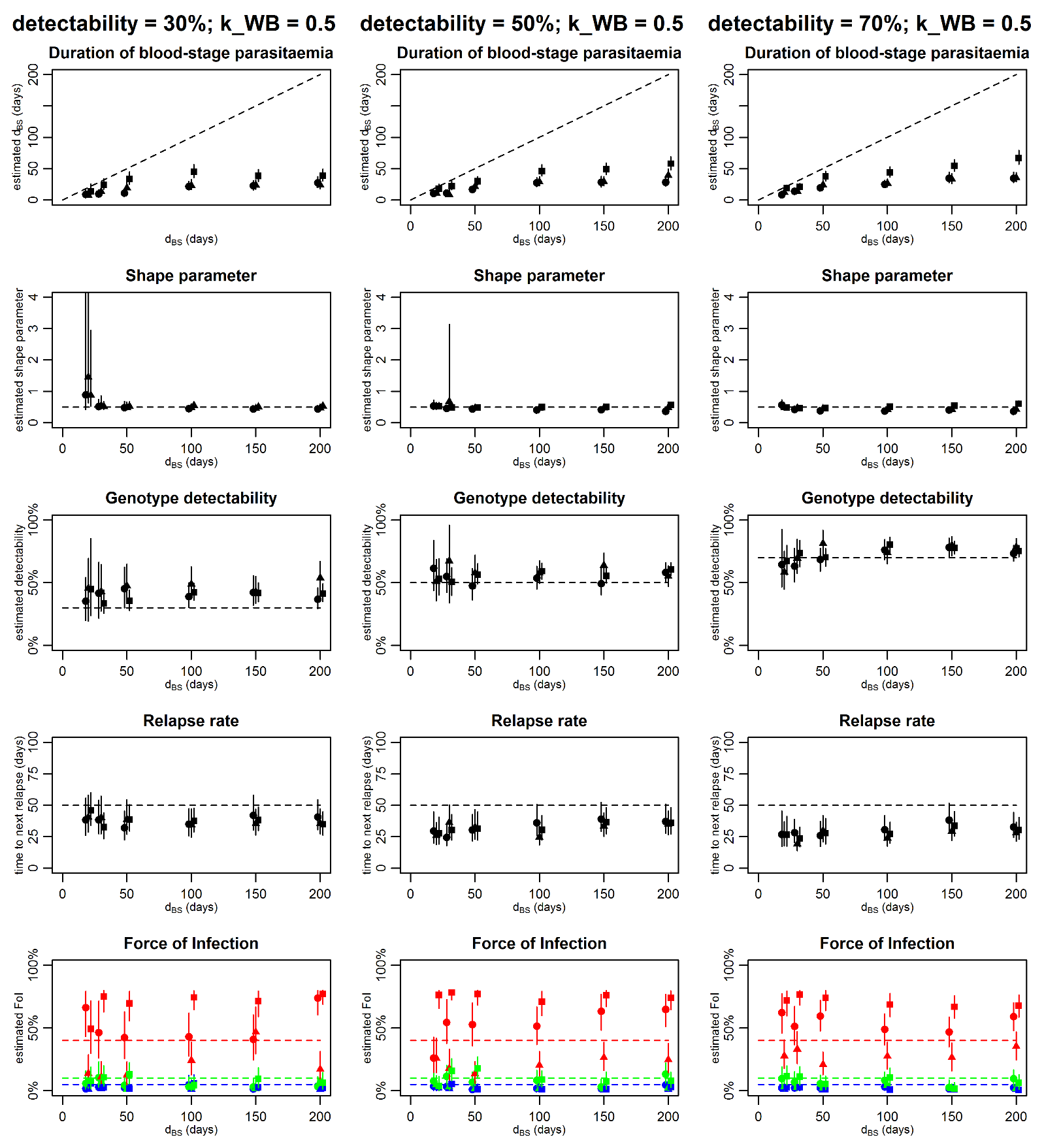
**

Figure S3.6: Validation of the *P. vivax* model with Weibull distributed duration of blood-stage infection using simulated data with a shape parameter *k_WB_* = 0.5. The points and vertical bars represent the median and 95% credible intervals of the estimated posterior distributions. For each combination of parameters, the model was fitted to simulated data assuming homogeneous (circles), heterogeneous (triangles) or seasonal (squares) exposure to infectious mosquito bites. Each column presents the results of simulations for a different value of genotype sensitivity. The dashed lines denote the values of the global parameters used for simulating the data.

**
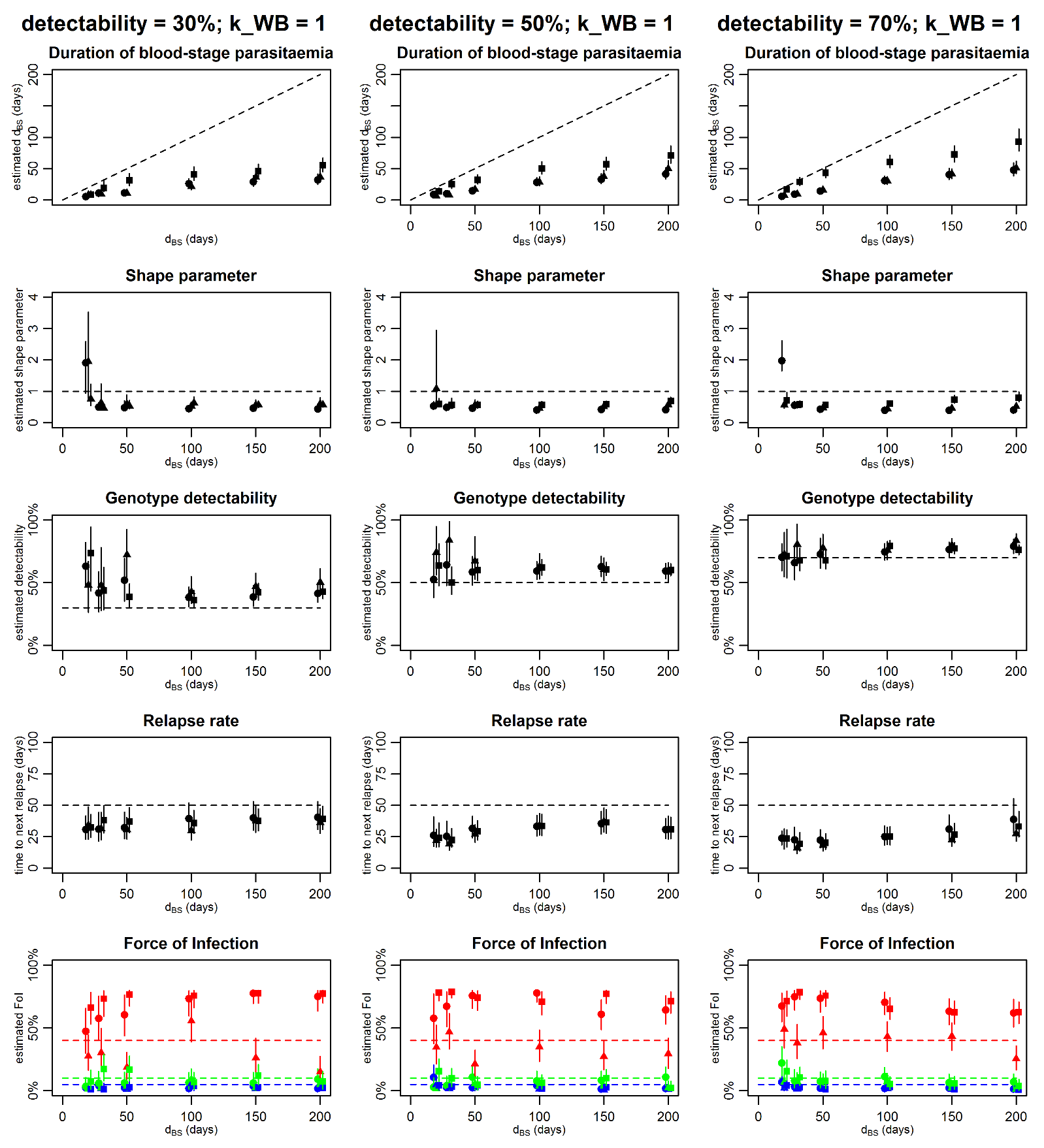
**

Figure S3.7: Validation of the *P. vivax* model with Weibull distributed duration of blood-stage infection using simulated data with a shape parameter *k_WB_* = 1. The points and vertical bars represent the median and 95% credible intervals of the estimated posterior distributions. For each combination of parameters, the model was fitted to simulated data assuming homogeneous (circles), heterogeneous (triangles) or seasonal (squares) exposure to infectious mosquito bites. Each column presents the results of simulations for a different value of genotype sensitivity. The dashed lines denote the values of the global parameters used for simulating the data.

**
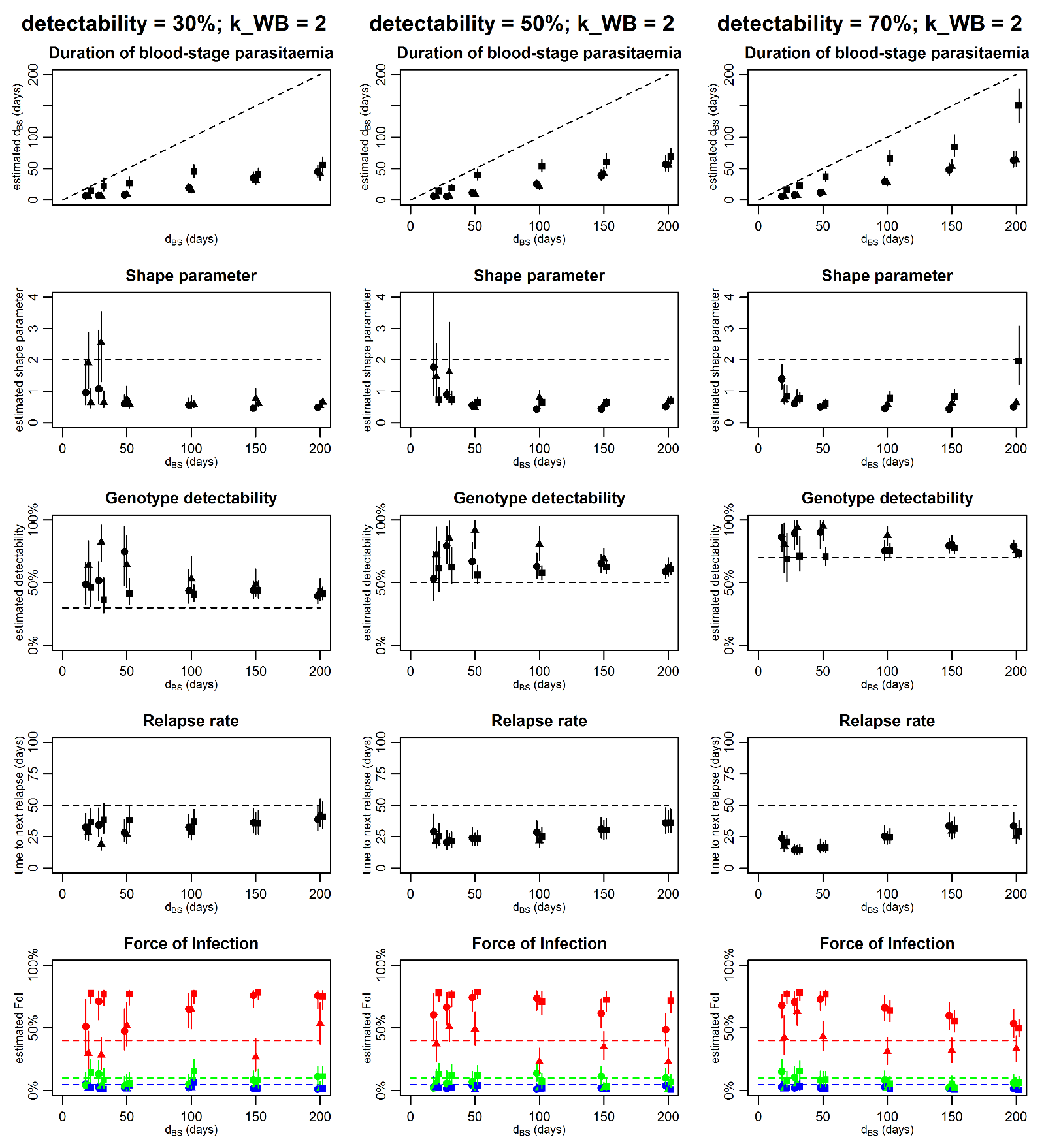
**

Figure S3.8: Validation of the *P. vivax* model with Weibull distributed duration of blood-stage infection using simulated data with a shape parameter *k_WB_* = 2. The points and vertical bars represent the median and 95% credible intervals of the estimated posterior distributions. For each combination of parameters, the model was fitted to simulated data assuming homogeneous (circles), heterogeneous (triangles) or seasonal (squares) exposure to infectious mosquito bites. Each column presents the results of simulations for a different value of genotype sensitivity. The dashed lines denote the values of the global parameters used for simulating the data.

**3.3. Sensitivity Analysis: Dependence of relapse probabilites on parameters**

Figure 4 presents estimates of the probability that *P. vivax* blood-stage parasites are attributable to relapses. These estimates will be dependent on the pattern of positive and negative samples and the estimated population-level parameters. In particular, it is possible that incorrectly estimated population-level parameters (e.g. *d_BS_ &* *f*) may contribute to errors in estimates of the probability that parasites detected in a sample originated from relapses.

To investigate this, we chose a fixed pattern for the presence or absence of a single-locus *P. vivax* genotype (Figure S3.9a) and examined how the estimated relapses probabilities changed as we varied the duration of blood-stage infection (*d_BS_*) and the time to next relapse (1/*f*). The results of this sensitivity analysis are presented in Figure S3.9b. For each of the three positive samples, the estimated probability showed very little variation as we varied *d_BS_*. Varying the time to next relapse did cause some variation in the estimated relapse probability. In particular, higher relapse frequencies led to slightly higher estimates of relapse probability.

**
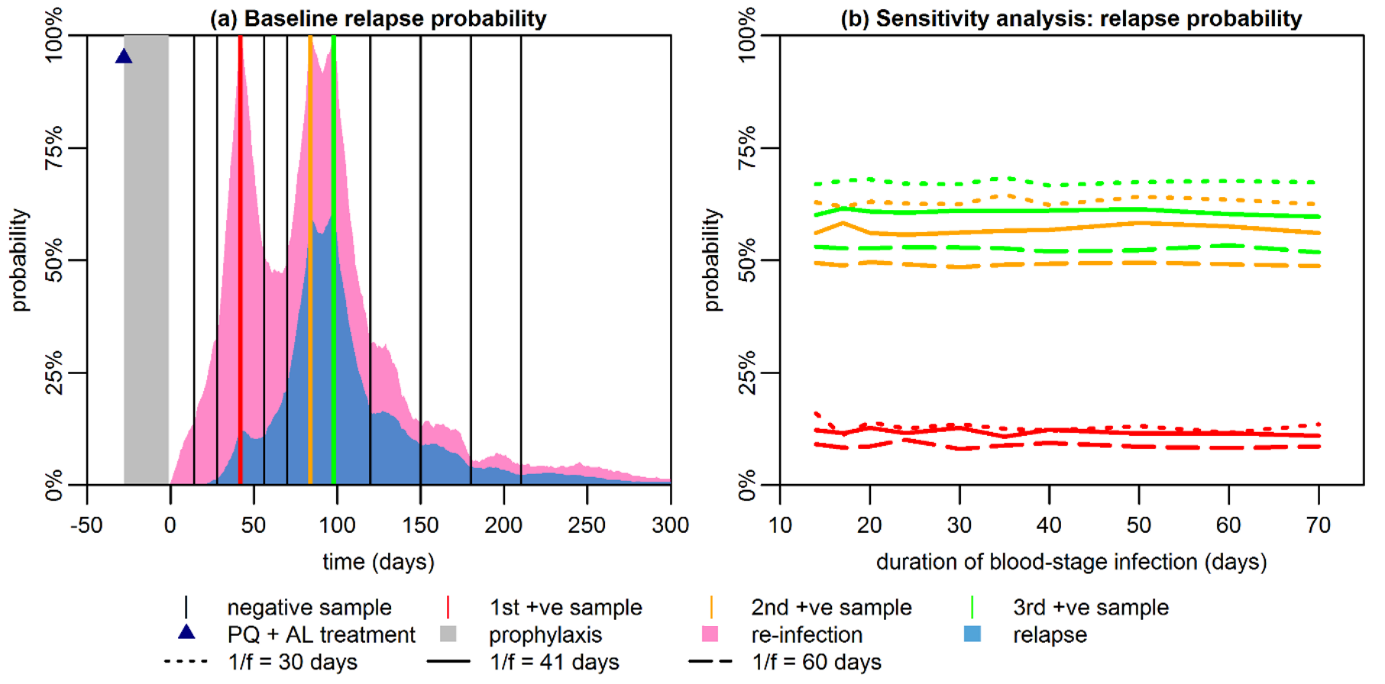
**

Figure S3.9: Dependence of estimated relapse probabilities on population-level parameters. (a) Estimated relapse probability for the baseline case with 1/*f* = 41 days and *d_BS_* = 24 days. (b) Change in estimated probability as the duration of blood-stage infection (*d_BS_*) and the time to next relapse (1/*f*) are varied.
