# Supplementary material for: Plasmodium vivax and Plasmodium falciparum infection dynamics: re-infections, recrudescences and relapses
Source: Malar J. 2018 Apr 17;17:170. doi: 10.1186/s12936-018-2318-1 (PMC5905131; doi:10.1186/s12936-018-2318-1)
Supplement: Supplementary file 4 — Additional file 4. Supplementary results. [file 12936_2018_2318_MOESM4_ESM.docx]

***Plasmodium vivax* and *Plasmodium falciparum* infection dynamics: re-infections, recrudescences and relapses**

*Michael White, Stephan Karl, Cristian Koepfli, Rhea Longley, Natalie E. Hofmann, Rahel Wampfler, Ingrid Felger, Tom Smith, Wang Nguitragool, Jetsumon Sattabongkot, Leanne Robinson, Azra Ghani, Ivo Mueller*

**Additional file 4: Supplementary Results**

**4.1. Parameter estimates: Maprik, Papua New Guinea**

The population-level parameter estimates for the model applied to *P. falciparum* and *P. vivax* data from Papua New Guinea are reported in Table 2. They are reported again in Table S4.1 with the inclusion of the genotype-specific force of infection for the 14 most common genotypes.

Table S4.1: Population-level and genotype-level parameter estimates for the *P. falciparum* and *P. vivax* models fitted to data from Maprik, Papua New Guinea. Parameters are presented as estimated posterior medians with 95% credible intervals.

| **description** | **parameter** | **prior** | ***P. falciparum*** | ***P. vivax*** |
| --- | --- | --- | --- | --- |
| blood-stage duration (days) | *d_BS_* | 60 (16, 132) | 36 (29, 44) | 24 (21, 28) |
| shape parameter | *κ* | 2 (0.54, 4.38) | 0.50 (0.45, 0.57) | 0.54 (0.49, 0.62) |
| detectability (*Pv*) | *q* | 48% (42%, 54%) | – | 44% (40%, 49%) |
| detectability (*Pf*) | *q* | 70% (64%, 76%) | 66% (60%, 71%) | – |
| time to relapse (days) | 1/*f* | 50 (19, 96) | – | 41 (35, 49) |
| liver-stage duration (days) | 1/*γ_L_* | 250 (195, 312) | – | 383 (313, 467) |
| genotype-specific FoI (year^-1^) | *λ^g^* |  |  |  |
| F359.1 / M262.9 |  | 0.17 (0.01, 0.92) | 0.602 (0.414 0.847) | 1.820 (1.368, 2.457) |
| F371.6 / M236.2 |  | 0.17 (0.01, 0.92) | 0.203 (0.062, 0.371) | 1.042 (0.734, 1.442) |
| D415.9 / M273.9 |  | 0.17 (0.01, 0.92) | 0.062 (0.003, 0.190) | 0.550 (0.362, 0.822) |
| D217.7 / M256.6 |  | 0.17 (0.01, 0.92) | 0.039 (0.002, 0.143) | 0.465 (0.298, 0.707) |
| F336.9 / M316.3 |  | 0.17 (0.01, 0.92) | 0.047 (0.002, 0.163) | 0.203 (0.108, 0.332) |
| D270.9 / M319.2 |  | 0.17 (0.01, 0.92) | 0.022 (0.001, 0.100) | 0.142 (0.070, 0.241) |
| F517.9 / M366.2 |  | 0.17 (0.01, 0.92) | 0.022 (0.001, 0.092) | 0.128 (0.055, 0.231) |
| D336.6 / M250.5 |  | 0.17 (0.01, 0.92) | 0.015 (0.001, 0.076) | 0.102 (0.039, 0.193) |
| D381.2 / M313.1 |  | 0.17 (0.01, 0.92) | 0.015 (0.001, 0.078) | 0.063 (0.013, 0.138) |
| D300.7 / M325.2 |  | 0.17 (0.01, 0.92) | 0.015 (0.001, 0.073) | 0.060 (0.012, 0.135) |
| D334.4 / M270.9 |  | 0.17 (0.01, 0.92) | 0.015 (0.001, 0.070) | 0.030 (0.002, 0.095) |
| D339.6/ M306.9 |  | 0.17 (0.01, 0.92) | 0.015 (0.001, 0.074) | 0.014 (0.001, 0.057) |
| D341.7 / M337.2 |  | 0.17 (0.01, 0.92) | 0.013 (0.000, 0.065) | 0.015 (0.001, 0.061) |
| D375.9 / M370.8 |  | 0.17 (0.01, 0.92) | 0.013 (0.001, 0.067) | 0.011 (0.000, 0.050) |

The model and data were investigated in more detail by fitting two variants. Firstly we allowed for age-specific variation in the duration of blood-stage infection for both *P. vivax* and *P. falciparum*. This was done by splitting participants from the Papua New Guinean cohort into three categories according to age (Table S4.2). There was not substantial variation in the duration of blood-stage infection for children of different age groups. However it should be noted that all children in the cohort had a reasonably narrow age range from 5 – 10 years.

Secondly, we restricted the data by removing alternate samples. Thus, for example, a pattern of 0001110010 would become 00101. The estimated parameters on the restricted dataset are presented in Table S4.2. There were no major differences between the parameters estimated from the full data set and those estimated from the restricted data set, suggesting the method is reasonably robust. One possible exception may be in the estimated duration of *P. falciparum* blood-stage infection: 36 (95% CrI: 29, 44) days for the full data set; 27 (95% CrI: 22, 34) days for the restricted data set. This difference may be due to a tendency for dropped samples to make infections look shorter, i.e. 001100 becomes 010. This is a phenomenon that will be investigated further.

Table S4.2: Population-level and genotype-level parameter estimates for the *P. falciparum* and *P. vivax* models fitted to data from Maprik, Papua New Guinea. The model was fitted to a reduced data set ‘half data’ generated by removing alternate samples from the longitudinal sampling. The model was also fitted with ‘variable age’ groups to the entire data set. Parameters are presented as estimated posterior medians with 95% credible intervals.

|  |  | ***P. falciparum*** | | ***P. vivax*** | |
| --- | --- | --- | --- | --- | --- |
| **description** | **parameter** | **half data** | **variable age** | **half data** | **variable age** |
| blood-stage duration (days) | *d_BS_* | 27 (22, 34) |  | 25 (21, 29) |  |
| blood-stage duration (age 4.8-6.7 years ) | *d_BS_* |  | 40 (30, 53) |  | 28 (22, 35) |
| blood-stage duration (age 6.7-8.5 years) | *d_BS_* | – | 40 (31, 53) | – | 27 (22, 35) |
| blood-stage duration (age 8.5-10.4 years) | *d_BS_* | – | 34 (25, 45) | – | 24 (20, 31) |
| shape parameter | *κ* | 0.50 (0.45, 0.59) | 0.52 (0.47, 0.59) | 0.64 (0.54, 0.83) | 0.52 (0.47, 0.59) |
| detectability | *q* | 71% (64%, 76%) | 65% (60%, 70%) | 54% (48%, 59%) | 43% (38%, 47%) |
| time to relapse (days) | 1/*f* | – | – | 32 (26, 41) | 40 (34, 47) |
| liver-stage duration (days) | 1/*γ_L_* | – | – | 331 (267, 409) | 378 (308, 463) |
| genotype-specific FoI | *λ^g^* |  |  |  |  |
| F359.1 / M262.9 |  | 0.878 (0.562, 1.361) | 0.573 (0.380, 0.823) | 1.321 (0.930, 1.872) | 1.847 (1.387, 2.445) |
| F371.6 / M236.2 |  | 0.265 (0.077, 0.495) | 0.190 (0.052, 0.347) | 0.796 (0.517, 1.209) | 1.062 (0.755, 1.470) |
| D415.9 / M273.9 |  | 0.061 (0.003, 0.211) | 0.065 (0.004, 0.190) | 0.379 (0.221, 0.611) | 0.563 (0.366, 0.829) |
| D217.7 / M256.6 |  | 0.075 (0.004, 0.241) | 0.036 (0.002, 0.138) | 0.334 (0.191, 0.550) | 0.473 (0.305, 0.719) |
| F336.9 / M316.3 |  | 0.046 (0.001, 0.182) | 0.041 (0.002, 0.151) | 0.112 (0.037, 0.219) | 0.206 (0.108, 0.341) |
| D270.9 / M319.2 |  | 0.026 (0.001, 0.122) | 0.020 (0.001, 0.092) | 0.131 (0.053, 0.251) | 0.142 (0.070, 0.247) |
| F517.9 / M366.2 |  | 0.027 (0.001, 0.127) | 0.020 (0.001, 0.092) | 0.079 (0.017, 0.172) | 0.130 (0.058, 0.235) |
| D336.6 / M250.5 |  | 0.022 (0.001, 0.102) | 0.014 (0.001, 0.072) | 0.104 (0.029, 0.220) | 0.110 (0.045, 0.205) |
| D381.2 / M313.1 |  | 0.025 (0.001, 0.118) | 0.015 (0.001, 0.072) | 0.029 (0.001, 0.101) | 0.065 (0.012, 0.149) |
| D300.7 / M325.2 |  | 0.022 (0.001, 0.107) | 0.013 (0.000, 0.068) | 0.036 (0.002, 0.116) | 0.060 (0.010, 0.139) |
| D334.4 / M270.9 |  | 0.022 (0.001, 0.102) | 0.014 (0.001, 0.070) | 0.014 (0.001, 0.072) | 0.031 (0.002, 0.096) |
| D339.6/ M306.9 |  | 0.019 (0.001, 0.097) | 0.014 (0.001, 0.070) | 0.013 (0.001, 0.069) | 0.014 (0.001, 0.059) |
| D341.7 / M337.2 |  | 0.026 (0.001, 0.116) | 0.013 (0.000, 0.068) | 0.017 (0.001, 0.072) | 0.015 (0.001, 0.059) |
| D375.9 / M370.8 |  | 0.020 (0.000, 0.095) | 0.013 (0.001, 0.063) | 0.015 (0.000, 0.069) | 0.010 (0.000, 0.051) |

**4.2. Parameter estimates: Kanchanaburi and Ratchaburi, Thailand**

Table S4.3: Population-level and genotype-level parameter estimates for the *P. falciparum* and *P. vivax* models in Thailand. Parameters are presented as estimated posterior medians with 95% credible intervals.

|  |  |  | ***P. falciparum**** | ***P. vivax*** |
| --- | --- | --- | --- | --- |
| **description** | **parameter** | **prior** | **posterior** | **posterior** |
| blood-stage duration (days) | *d_BS_* | 60 (16, 132) | 135 (94, 191) | 29 (27, 32) |
| shape parameter | *κ* | 2 (0.54, 4.38) | 1.15 (0.83, 1.99) | 0.40 (0.38, 0.42) |
| detectability (*P. falciparum*) | *q* | 70% (64%, 76%) | 67% (61%, 73%) | – |
| detectability (*P. vivax*) | *q* | 48% (42%, 54%) | – | 38% (35%, 41%) |
| time to relapse (days) | 1/*f* | 50 (19, 96) | – | 55 (40, 80) |
| liver-stage duration (days) | 1/*γ_L_* | 250 (195, 312) | – | 226 (181, 278) |
| genotype-specific FoI (year^-1^) | *λ^g^* |  |  |  |
| Pf.B335.11 / Pv.G262.71 |  | 0.17 (0.01, 0.92) | 0.0181 | 0.0139 (0.0017, 0.0614) |
| Pf.G185.81 / Pv.G256.73 |  | 0.17 (0.01, 0.92) | 0.0054 | 0.0087 (0.0010, 0.0585) |
| Pf.G397.43 / Pv.B189.04 |  | 0.17 (0.01, 0.92) | 0.0054 | 0.0031 (0.0001, 0.0183) |
| Pf.G409.91/ Pv.G307.14 |  | 0.17 (0.01, 0.92) | 0.0054 | 0.0045 (0.0001, 0.0223) |
| Pf.B371.18 / Pv.B204.7 |  | 0.17 (0.01, 0.92) | 0.0036 | 0.0032 (0.0001, 0.0169) |
| Pf.G434.11 / Pv.B231.81 |  | 0.17 (0.01, 0.92) | 0.0036 | 0.0027 (0.0001, 0.0166) |
| Pf.B261.17 / Pv.B216.25 |  | 0.17 (0.01, 0.92) | 0.0018 | 0.0043 (0.0002, 0.0204) |
| Pf.B297.73 / Pv.B196.79 |  | 0.17 (0.01, 0.92) | 0.0018 | 0.0036 (0.0001, 0.0191) |
| Pf.G208.95 / Pv.B224.22 |  | 0.17 (0.01, 0.92) | 0.0018 | 0.0048 (0.0002, 0.0237) |
| Pf.G255.15 / Pv.B247.29 |  | 0.17 (0.01, 0.92) | 0.0018 | 0.0029 (0.0001, 0.0162) |
| Pf.G336.63 / Pv.B212.61 |  | 0.17 (0.01, 0.92) | 0.0018 | 0.0031 (0.0001, 0.0183) |
| Pf.G341.26 / Pv.B255.15 |  | 0.17 (0.01, 0.92) | 0.0018 | 0.0033 (0.0001, 0.0185) |
| Pf.G345.18 / Pv.B258.87 |  | 0.17 (0.01, 0.92) | 0.0018 | 0.0034 (0.0001, 0.0162) |
| Pf.G347.09 / Pv.B177.14 |  | 0.17 (0.01, 0.92) | 0.0018 | 0.0029 (0.0001, 0.0153) |

*There were too few cases of *P. falciparum* in Thailand for the genotype-specific force of infection to be reliably estimated. Therefore we estimate it as the ratio of number of infections with that genotype divided by the person year at risk. The data augmentation process was then only applied to cases where individuals were positive for a given genotype.

**4.3. Individual-level infection dynamics**

In the main manuscript, infection dynamics were presented for 2/504 Papua New Guinean participants and 1/999 Thai participants. Here we provide additional examples of infection dynamics from another 4 Papua New Guinean and 2 Thai individuals.

**
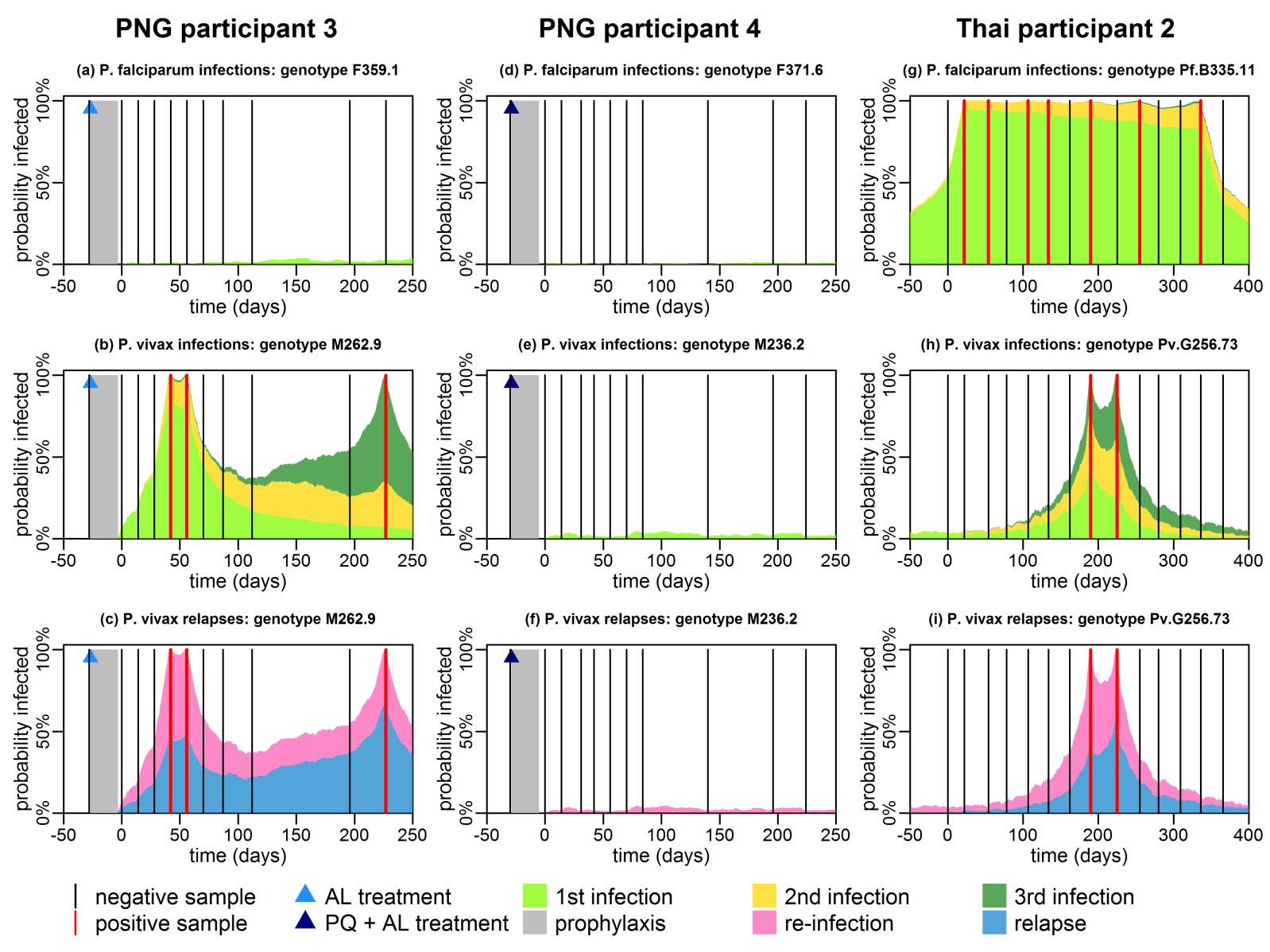
**

Figure S4.1: Infection dynamics of single *P. falciparum* and *P. vivax* genotypes in three individuals. For each genotype within each individual, the colours denote whether the parasites predicted to be present at a given time arose from that individual’s first, second or third infection. Each *P. falciparum* infection comes from a separate mosquito bite. Each *P. vivax* infection comes from either a new mosquito bite or a relapse. The bottom row depicts the probability that a new infection with a *P. vivax* genotype is due to re-infection from mosquito or relapse. The grey region denotes the period of prophylaxis when participants were treated with AL and chloroquine, and either primaquine or a placebo. During the period of follow-up AL treatment was administered by the investigators to some of the Papua New Guinean participants.

**
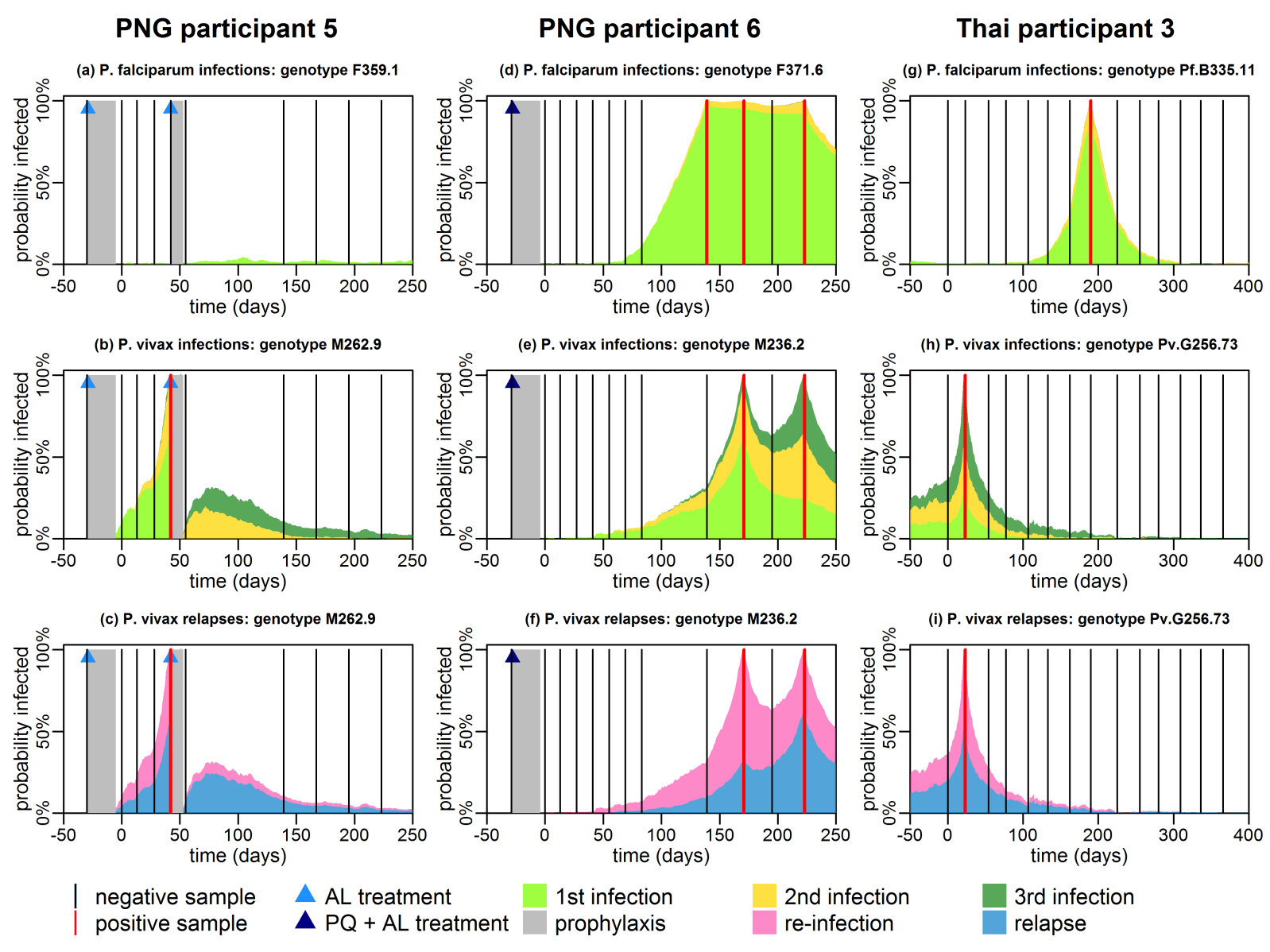
**

Figure S4.2: Infection dynamics of single *P. falciparum* and *P. vivax* genotypes in three individuals. For each genotype within each individual, the colours denote whether the parasites predicted to be present at a given time arose from that individual’s first, second or third infection. Each *P. falciparum* infection comes from a separate mosquito bite. Each *P. vivax* infection comes from either a new mosquito bite or a relapse. The bottom row depicts the probability that a new infection with a *P. vivax* genotype is due to re-infection from mosquito or relapse. The grey region denotes the period of prophylaxis when participants were treated with AL and chloroquine, and either primaquine or a placebo. During the period of follow-up AL treatment was administered by the investigators to some of the Papua New Guinean participants.

**
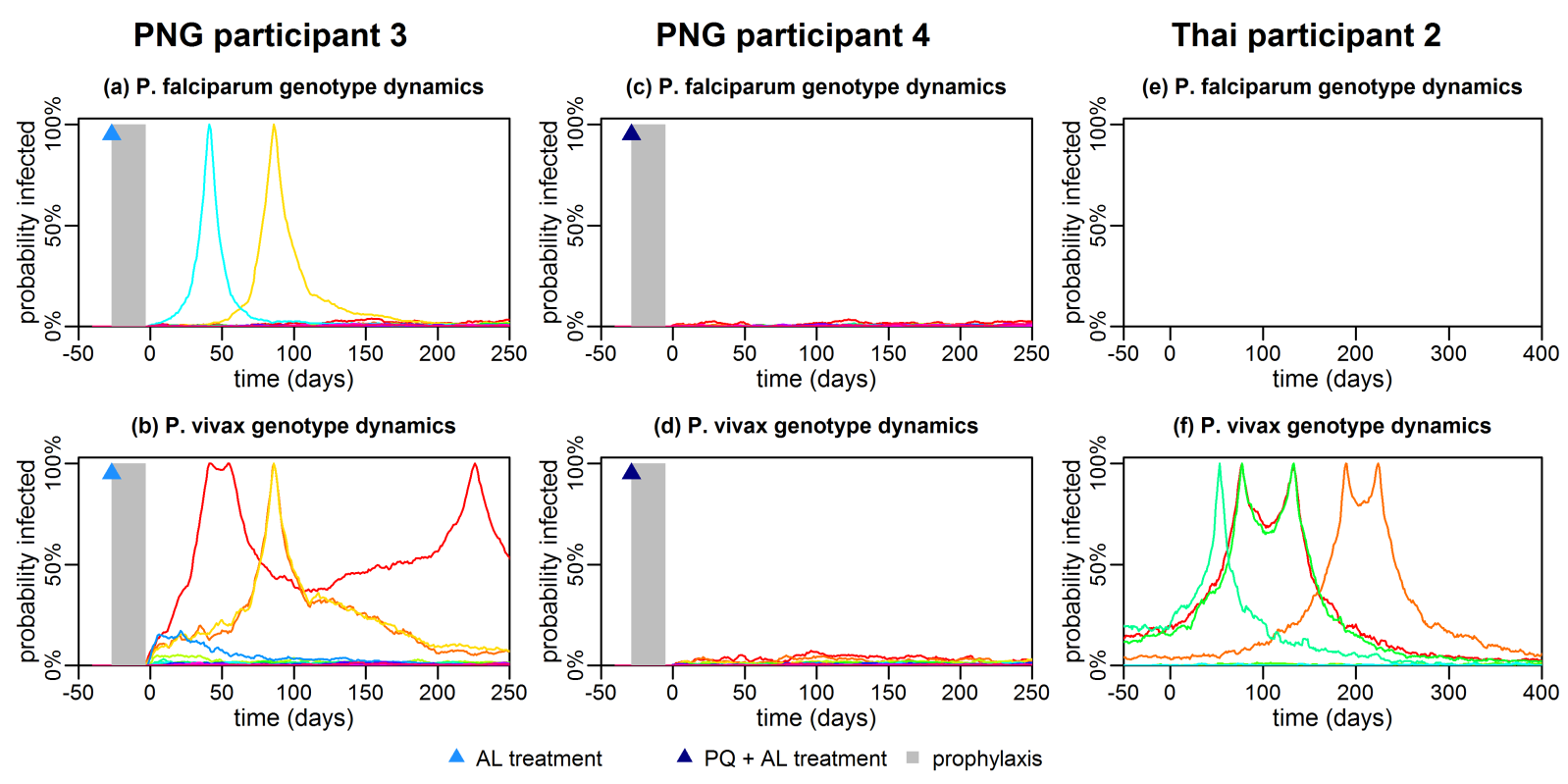
**

Figure S4.3: Infection dynamics of multiple *P. falciparum* and *P. vivax* genotypes in three individuals. Each genotype is represented by a different colour.

**
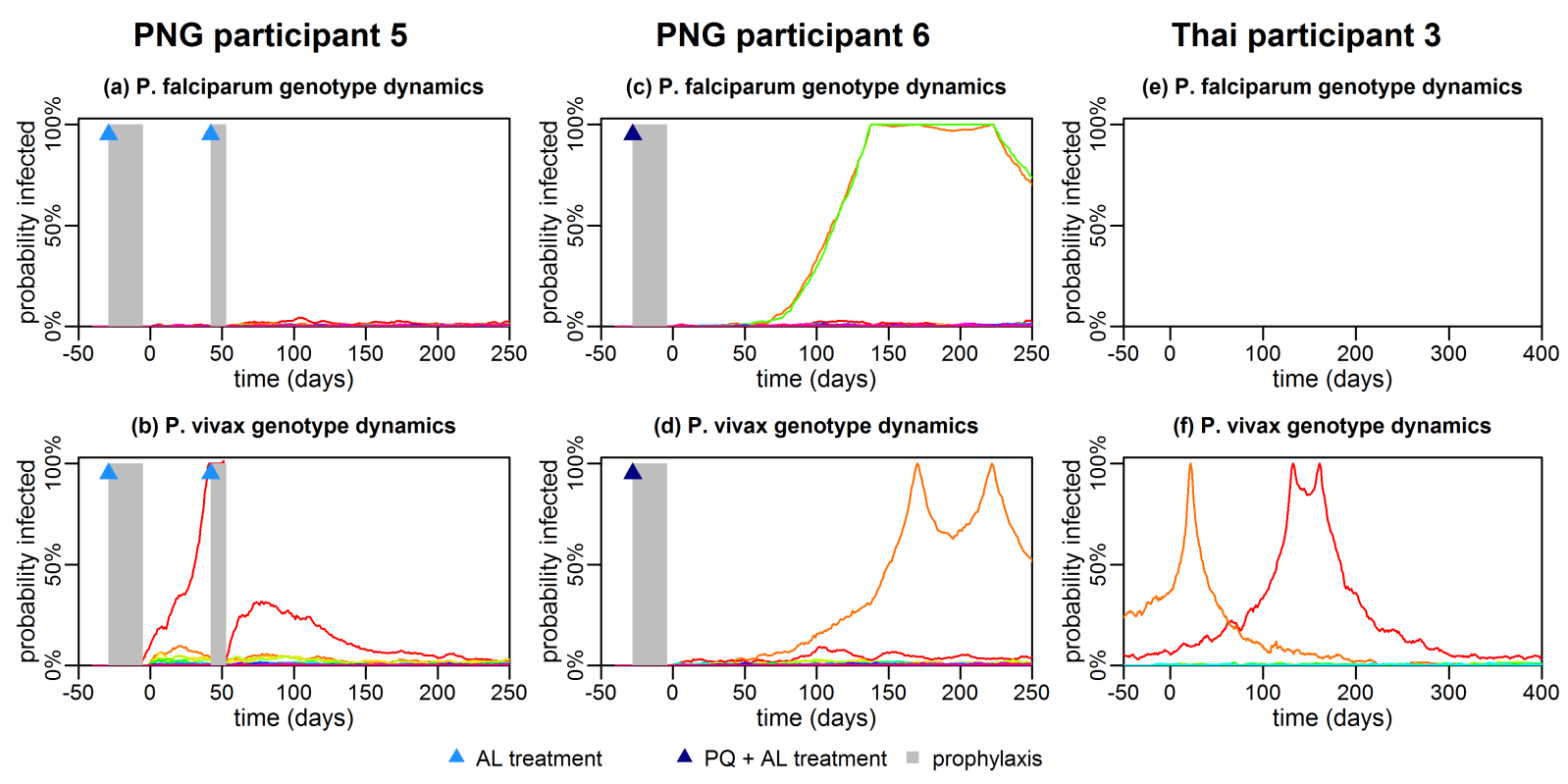
**

Figure S4.4: Infection dynamics of multiple *P. falciparum* and *P. vivax* genotypes in three individuals. Each genotype is represented by a different colour.

**4.4. Genotype prevalence**

The model predicted prevalence of each genotype is shown in Figure 6 of the main manuscript. Notably it accounts for the proportion of genotypes missed in samples due to imperfect detectability. For comparison, Figure S4.5 shows the estimated genotype prevalence based on the data. The difference between the genotype prevalence estimated from the data and predicted by the model is due to imperfect detectability.


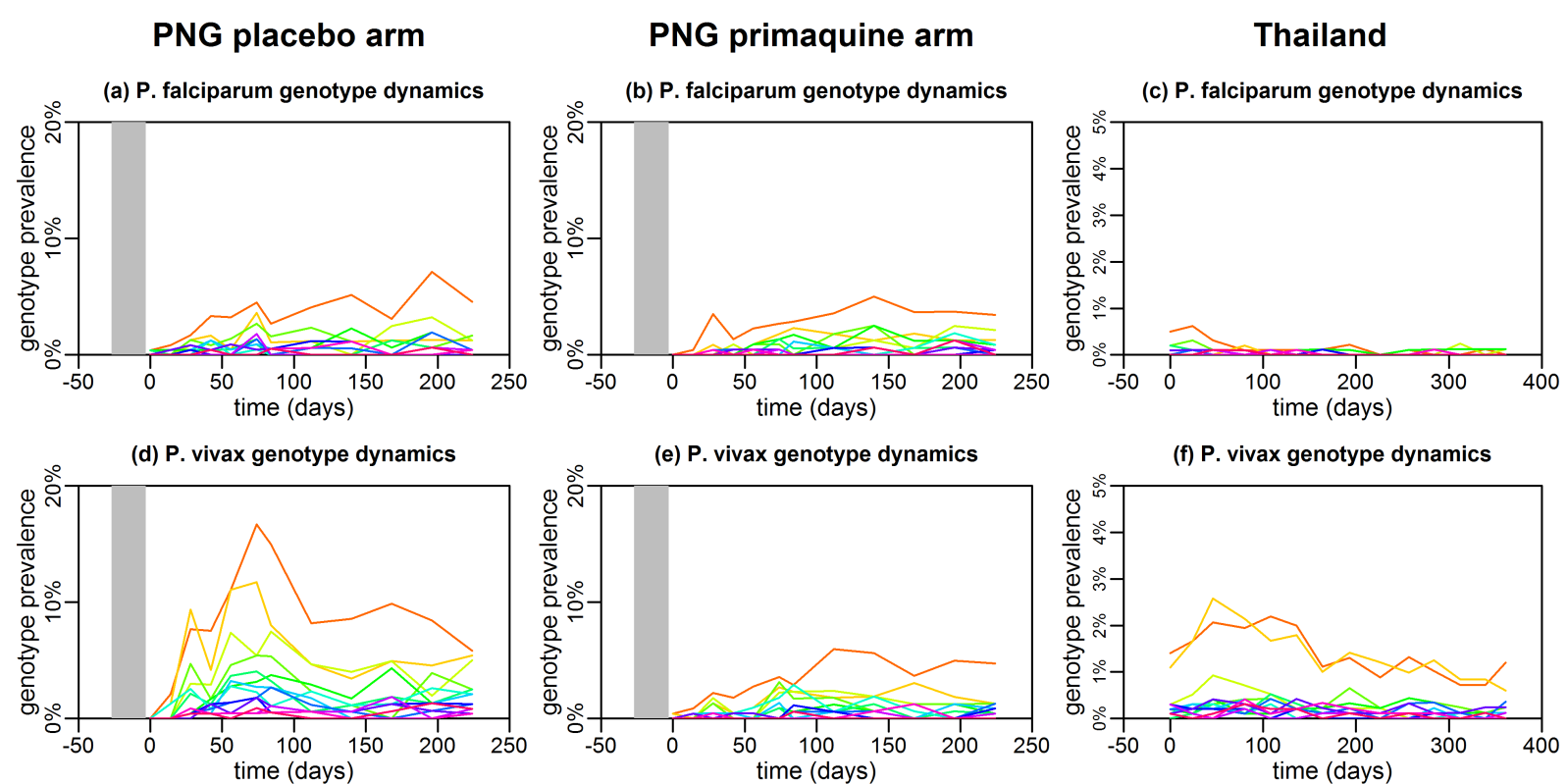


Figure S4.5: Multiplicity of infection (MoI) based on positive identified genotypes in each sample in the Thai cohort. Each colour represents a different genotype. The grey shaded region denotes the period of prophylactic protection.
